# Size Control in the Colloidal Synthesis of Plasmonic Magnesium Nanoparticles

**DOI:** 10.1021/acs.jpcc.1c07544

**Published:** 2021-12-28

**Authors:** Elizabeth R. Hopper, Thomas M. R. Wayman, Jérémie Asselin, Bruno Pinho, Christina Boukouvala, Laura Torrente-Murciano, Emilie Ringe

**Affiliations:** † Department of Materials Science and Metallurgy, University of Cambridge, 27 Charles Babbage Road, Cambridge CB3 0FS, United Kingdom; ‡ Department of Earth Sciences, University of Cambridge, Downing Street, Cambridge CB2 3EQ, United Kingdom; § Department of Chemical Engineering and Biotechnology, University of Cambridge, Philippa Fawcett Drive, Cambridge CB3 0AS, United Kingdom

## Abstract

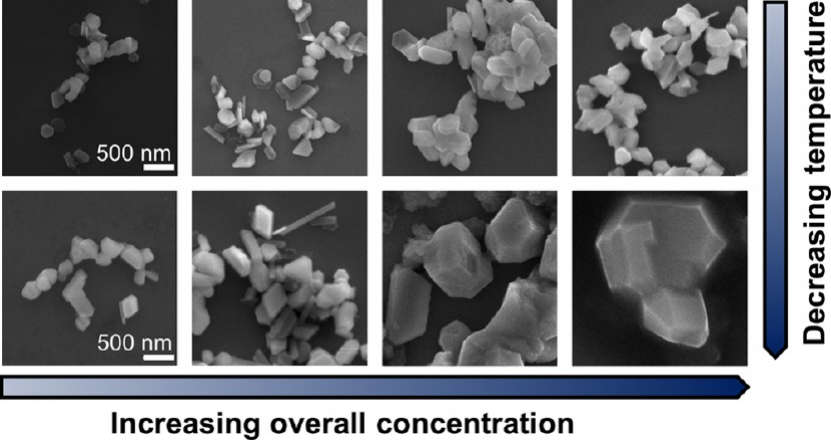

Nanoparticles of plasmonic materials can sustain oscillations of their free electron density, called localized surface plasmon resonances (LSPRs), giving them a broad range of potential applications. Mg is an earth-abundant plasmonic material attracting growing attention owing to its ability to sustain LSPRs across the ultraviolet, visible, and near-infrared wavelength range. Tuning the LSPR frequency of plasmonic nanoparticles requires precise control over their size and shape; for Mg, this control has previously been achieved using top-down fabrication or gas-phase methods, but these are slow and expensive. Here, we systematically probe the effects of reaction parameters on the nucleation and growth of Mg nanoparticles using a facile and inexpensive colloidal synthesis. Small NPs of 80 nm were synthesized using a low reaction time of 1 min and ∼100 nm NPs were synthesized by decreasing the overall reaction concentration, replacing the naphthalene electron carrier with biphenyl or using metal salt additives of FeCl_3_ or NiCl_2_ at longer reaction times of 17 h. Intermediate sizes up to 400 nm were further selected via the overall reaction concentration or using other metal salt additives with different reduction potentials. Significantly larger particles of over a micrometer were produced by reducing the reaction temperature and, thus, the nucleation rate. We showed that increasing the solvent coordination reduced Mg NP sizes, while scaling up the reaction reduced the mixing efficiency and produced larger NPs. Surprisingly, varying the relative amounts of Mg precursor and electron carrier had little impact on the final NP sizes. These results pave the way for the large-scale use of Mg as a low-cost and sustainable plasmonic material.

## Introduction

Nanomaterials, including nanoparticles (NPs), have attracted growing interest owing to their vastly different properties compared to bulk materials. The ability of plasmonic NPs to sustain oscillations of their free electron density, called localized surface plasmon resonances (LSPRs), gives them broad applicability in areas including chemical and biological sensing,^1,2^ surface-enhanced spectroscopies,^3−5^ photothermal cancer therapy,^6^ and photocatalysis.^7,8^ Recently, Mg has attracted growing interest as a plasmonic material due to its excellent plasmonic properties across the ultraviolet, visible, and near-infrared (UV–vis–NIR) wavelengths, as well as earth-abundance and low cost compared to the common plasmonic materials Au and Ag.^9−11^

The resonant LSP frequencies of plasmonic NPs are highly dependent on size and shape. Mg’s plasmonic properties have been confirmed by far-field optical scattering, electron energy loss spectroscopy measurements, and numerical results.^9,12^ Specifically, changing Mg NP size maneuvers the position of the dipole resonance across the UV–vis–NIR wavelengths since the dielectric function of Mg enables resonances across this entire range, unlike most other plasmonic metals. Thus, synthetic control over size and shape is vital, yet largely unexplored for Mg.

Recent studies of plasmonic Mg nanostructures have relied on top-down fabrication methods such as electron-beam lithography,^13−16^ hole-mask lithography,^15^ ion-beam milling,^17^ or mechanical milling.^18^ These methods are often expensive, time consuming, and can result in poor crystallinity that degrades the plasmonic response. Alternatively, gas-phase methods provide good size and shape control^19−22^ but with low yields and extensive aggregation.^23^ In contrast, colloidal NP syntheses are inexpensive, facile, scalable, and have the potential for substantial size and shape control, as has been demonstrated extensively for Au and Ag.^24−29^

Mg NPs can be synthesized via the inert atmosphere reduction of an organometallic precursor such as di-*n*-butylmagnesium with an electron donor, for instance, lithium naphthalenide (LiNapht), as in the work of Rieke et al., Biggins et al., and recent syntheses in hydrogen storage research.^10−12,30−37^ Using this method, we previously reported the production of ∼300 nm Mg NPs with a variety of shapes, including hexagonal platelets and folded rods, dictated by Mg’s hexagonal close-packed crystal structure and twinning on various crystal planes.^10,12^ These NPs form a self-limiting oxide layer of less than 10–20 nm that protects the metallic Mg core, rendering the NP stable when dispersed in organic solvents such as ethanol and isopropanol (IPA) or dried in air.^9,10,12^

Here, we report a systematic study into the effects of colloidal synthesis parameters on the resulting Mg NP sizes. By controlling the nucleation and growth rates, Mg NP sizes between 80 nm and over a micrometer were obtained selectively. Small NPs of ∼100 nm were obtained at reaction times of several minutes; beyond this time, NPs continued to grow to a final size 400 nm. The final NP sizes after 17 h of reaction were reduced to 100 nm by decreasing the overall reaction concentration, by replacing the naphthalene electron carrier with biphenyl or using metal salt additives of FeCl_3_ or NiCl_2_, which form nuclei onto which Mg NPs grow. Intermediate sizes were further selected via the overall reaction concentration or using other metal salt additives with different reduction potentials, changing the concentration of nuclei formed. Final NP sizes were also significantly increased by reducing the reaction temperature to 0 °C and varying the overall concentration, in this case, by tuning the nucleation rate. We further show that increasing the solvent coordination leads to smaller NPs, while scaling up the reaction (increasing reaction volume and flask size) produces larger NPs due to the reduced mixing efficiency. Surprisingly, varying the relative amounts of Mg precursor and electron carrier had little impact on the final NP sizes. This synthetic control over Mg NP size lays the foundation for facile and inexpensive production of Mg NPs with tuneable plasmonic properties.

## Methods

### Materials

Lithium pellets (99%), naphthalene, biphenyl, phenanthrene, anthracene, 1.0 M di-*n*-butylmagnesium (MgBu_2_) in heptane, anhydrous tetrahydrofuran (THF), 1,2-dimethoxyethane (glyme), 2-methoxyethyl ether (diglyme), 1,4-dioxane, dibutyl ether, FeCl_3_ (99.9%), NiCl_2_·6H_2_O (99.9%), FeCl_2_ (98%), VCl_2_ (85%), AlCl_3_ (98.5%), and anhydrous isopropanol (IPA) were purchased from Sigma-Aldrich and used as supplied. All glassware was washed with aqua regia (1:3 HNO_3_/HCl) and flame-dried under vacuum before use. (Caution: aqua regia solutions are dangerous and should be used with extreme care; these solutions should never be stored in closed containers.)

### Synthesis

Mg NPs were synthesized by the reduction of an organometallic precursor, as previously reported.^10,12^ For a standard reaction, 0.028 g of lithium (4.05 mmol), 0.530 g of naphthalene (4.05 mmol), and 5 mL of anhydrous THF were added to a 25 mL Schlenk flask under an Ar atmosphere and sonicated for 1 h, producing a dark green lithium naphthalenide (LiNapht) solution. Under stirring, 5.75 mL of THF was injected followed by 1.75 mL of MgBu_2_ in heptane (1.0 M, 1.75 mmol). When an additive was used, 0.09 mmol of metal chloride salt (14.3 mg of FeCl_3_, 20.9 mg of NiCl_2_·6H_2_O, 11.2 mg of FeCl_2_, 12.6 mg of VCl_2_, or 11.7 mg of AlCl_3_) was weighed into a separate dried flask under Ar and 5.75 mL of THF added. The flask was sonicated for 1 h to dissolve the salt, and the resultant solution was injected into the LiNapht simultaneously with MgBu_2_ in place of the second THF injection.

The reaction was stirred for 17 h at room temperature (20 °C) before quenching LiNapht and byproducts by addition of 6.25 mL of anhydrous IPA, leaving a gray solution. The solid gray product was recovered by centrifugation and then residual Li, naphthalene, and organic byproducts removed by repeated centrifugation and redispersion steps in anhydrous THF twice and anhydrous IPA twice, before redispersing in anhydrous IPA.

The synthesis was modified as described in the text: by changing the reaction time before quenching, reaction scale (increasing all quantities proportionally with the increase in flask size), amounts of all reagents at constant reaction volume, reaction temperature, electron carrier (replacing naphthalene with equal moles of biphenyl, phenanthrene, or anthracene), the addition of metal salt additives (1:20 molar ratio with MgBu_2_), changing the solvent (replacing THF with glyme or diglyme), concentration of MgBu_2_ (changing the volume of MgBu_2_ injected), or concentration of naphthalene (changing the amount of naphthalene added to the flask).

### Characterization

Samples were drop-cast onto Si wafers for SEM imaging, performed on a Quanta-650F field emission gun SEM, operated at 5 kV, and equipped with an Everhart–Thornley detector for secondary electron imaging. HAADF-STEM images, STEM-EDS line scans, and STEM-EELS were acquired at 200 kV on a FEI Osiris STEM equipped with a Bruker Super-X quadruple EDS detector and a Gatan Enfinium ER 977 electron spectrometer, for NPs drop-cast on a Cu-supported lacey ultrathin carbon membrane. For each synthesis, at least 50 hexagonal platelets and 50 rods with clearly visible edges were measured separately. NPs were classified as hexagonal platelets if they had six sides of approximately equal length and rod shaped if one dimension was elongated. The size of hexagonal platelets was defined as the distance between opposite corners; for rods, as the longest length. Although samples often appeared aggregated, NPs could be seen sufficiently clearly (with five or more corners visible) to make measurements. Few NPs lay flat on the support, potentially skewing the size distribution to smaller sizes; however, as the degree of aggregation in each sample was very similar, we expect that this will not affect the trends seen.

To assess NP size variation, Welch’s *t*-test and Welch’s ANOVA test followed by Games-Howell pairwise comparisons post hoc tests were performed using IBM SPSS Statistics software for pairs and sets of samples, respectively, using a 95% confidence interval. The tests assume that the distributions are normal (appropriate for sample size *N* > 30), that the data were obtained from a random sample, and that the individual observations are independent (true when the sample size *N* < 10% of the population).

XRD data were collected using a Bruker D8 Advanced diffractometer equipped with a Cu Kα X-ray tube and a Vantec position-sensitive detector in Bragg–Brentano geometry over a 20–80° range with a resolution of 0.2°/step. Sample suspensions were spread on top of a silicon low background sample holder and left to dry. Silicon powder was sprinkled on top of the dry specimen as an internal standard. A LaB_6_ 660b NIST standard^38^ was used to model the instrumental contribution to peak broadening using fundamental parameters approach^39^ with software Topas Academic V6.^40^ Lattice parameters, scaling factors, and no other structural parameters were refined for each phase in the Rietveld refinements of the data presented here. A Chebyshev function with five parameters was used to fit the background. Si internal standard lattice parameter was used to correct for sample displacement. The sample contribution to peak broadening was assumed to be isotropic and related to crystallite size (CS) only, related to the Lorentzian full width at half-maximum (FWHM) Γ_L_ as in the Scherrer eqs 1 and 2(40)
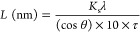
1
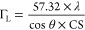
2where *L* is the mean size of the ordered (crystalline) domains, *K*_s_ is a shape factor constant in the range (typically 0.9), λ is the X-ray wavelength, and τ is the peak width in radians at FWHM. Estimated standard deviations from Rietveld calculations have no bearing on the precision or accuracy, being merely related to the mathematical fit of the model.^41^ For a typical laboratory X-ray diffraction instrument, the Scherrer analysis provides sensitivity to CS in the 1–100 nm range,^42^ the upper limit being set by the instrumental broadening. The smaller the crystal size, the less the Scherrer CS size value is affected by how the instrumental broadening is defined.

Inductively coupled plasma mass spectrometry (ICP-MS) was performed using a PerkinElmer NexION 2000-S mass spectrometer. Samples were digested in an aqueous matrix with 10% v/v of ultrapure nitric acid (max 10 ppt metal traces) for at least 10 min before analysis. Extinction spectra were measured using a Thermo Scientific Evolution 220 spectrophotometer. Fourier transform infrared (FT-IR) spectra of dried Mg NP samples were taken on a Nicolet iS5 FT-IR spectrometer.

Dark-field optical scattering spectra were obtained on Mg NPs drop-cast onto a glass coverslip and left to dry in air. Scattering spectra were obtained on an optical microscope equipped with a halogen lamp, dark-field condenser (numerical aperture, NA, 0.85–0.95), 100× oil immersion objective (variable NA set to <0.8), Princeton Instruments Isoplane spectrometer (50 grooves/mm grating), and ProEM 1024 × 1024 pixels electron multiplied charge-coupled device (EMCCD) described elsewhere.^43^ The exposure time was set to 1 s with four frames accumulated per position. Color videos were recorded using a Thorlabs Kiralux 5.0 MP Monochrome CMOS camera, model number CS505CU. NP tracking analysis (NTA) was performed at 403 nm on a Malvern NanoSight NS300.

### Numerical Methods

Extinction spectra of hexagonal platelets were calculated using the discrete dipole approximation in DDSCAT,^44,45^ using the frequency-dependent refractive index of metallic Mg from Palik,^46^ and a surrounding environment of a refractive index of 1.3776 corresponding to IPA.^47^ Shapes were generated using the open-source Crystal Creator software,^48^ and the aspect ratio of the hexagonal platelet was kept constant with thickness 0.1 times the tip-to-tip distance. All interdipole distances were 2.0 nm making the number of dipoles between ∼40 000 (for tip-to-tip length 190 nm) and 320 000 (for tip-to-tip length 370 nm).

## Results and Discussion

**Figure 1:**
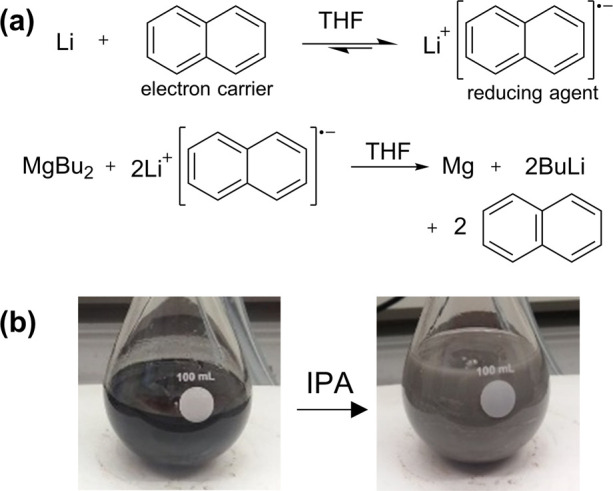
Synthesis of Mg NPs. (a) Reaction scheme for Mg NP synthesis: formation of LiNapht from the reaction of Li and naphthalene, followed by injection of MgBu_2_ resulting in Mg NP formation. (b) Reaction mixture before (left) and after (right) quenching with IPA.

Colloidal Mg NPs were synthesized by the room-temperature reduction of an organometallic precursor, di-*n*-butylmagnesium (MgBu_2_), by an aromatic electron carrier anion, naphthalenide (Figure 1a). First, the reducing agent, a radical anion salt lithium naphthalenide (LiNapht), was generated by reducing the electron carrier naphthalene with metallic Li, by sonicating for 1 h in tetrahydrofuran (THF), after which it was assumed that LiNapht formation was complete. Then, the organometallic precursor, MgBu_2_, was injected into the solution under magnetic stirring. As the reduction of MgBu_2_ to Mg^0^ requires two electrons, a molar ratio of 2.3:2.3:1 Li/naphthalene/MgBu_2_ was typically used.

The reaction between the organic radical anion and the organometallic precursor produces Mg^0^, after which NP formation proceeds via a nucleation and growth mechanism. The reaction is then quenched with IPA to deactivate the pyrophoric LiNapht and BuLi (Figure 1b). After purification by centrifugation and redispersion steps in anhydrous THF twice and anhydrous IPA twice, NPs were characterized by scanning electron microscopy (SEM) or high-angle annular dark-field scanning transmission electron microscopy (HAADF-STEM). NPs were confirmed to be metallic Mg by X-ray diffraction (XRD, Figure S1) and STEM electron energy loss spectroscopy (STEM-EELS, Figure S2). No stabilizing ligands were seen in the Fourier transform infrared (FT-IR) spectra of dried NPs (Figure S3); the broad peak below 850 cm^–1^ corresponds to the stretching vibration of the surface Mg–O–Mg and the reduced transmittance below 1500 cm^–1^ may be attributed to surface oxide or adsorbed naphthalene.^49^ The peaks observed around 3000 cm^–1^ for both MgO and naphthalene likely arise from surface hydroxyl groups that are not, or less, present on Mg NPs. Although the absence of ligands means that NPs are not colloidally stable, they can be easily redispersed by sonication (Figure S4); the resulting dispersion contains single NPs, as shown by NP tracking analysis (NTA, Figure S5), which measures comparable NP sizes to those of single NPs measured by SEM. When the dispersion is drop-cast onto a glass slide, a significant fraction of the aggregates seen are created upon drying (see the Supporting Video).

The reaction yield, measured by inductively coupled plasma mass spectrometry (ICP-MS) after sample purification (during which some sample is inevitably lost), was typically between 10 and 50% (Figure S6). The shapes of NPs for all of the performed reactions consist of a mixture of single-crystal hexagonal platelets and several singly twinned rodlike shapes as previously reported.^12^ The rod shapes include tents, chairs, tacos, and kite shapes arising from twinning on the (101̅1), (101̅2), (101̅3), and (112̅1) planes, respectively. In all reported syntheses, these shapes were produced in approximately constant proportions of around 45% hexagonal platelets and 55% rodlike shapes as measured by SEM or HAADF-STEM.

The sizes of hexagonal platelets (NPs with six approximately equal angles and side lengths) and rods (NPs with one elongated dimension) were compared separately for each synthesis. Mean NP sizes and standard deviations are reported in the SI for each reaction. The reproducibility of NP sizes was confirmed by repeating a synthesis at a concentration of MgBu_2_ ([MgBu_2_]) of 0.14 M, with reagent ratios Li/Napht/MgBu_2_ = 2.3:2.3:1 and with a reaction volume of 12.5 mL in a 25 mL flask at room temperature four times: the mean sizes were statistically similar at the 95% confidence level as measured by Welch’s ANOVA test followed by Games-Howell pairwise comparison (mean sizes, standard deviations, and *p*-values reported in Figures S7 and S8, and Tables S1 and S2). Similarly, the average aspect ratios of rods (length/width) were constant at the 95% confidence level, being between 1.7 and 2.4 (Figure S9, Table S3).

**Figure 2:**
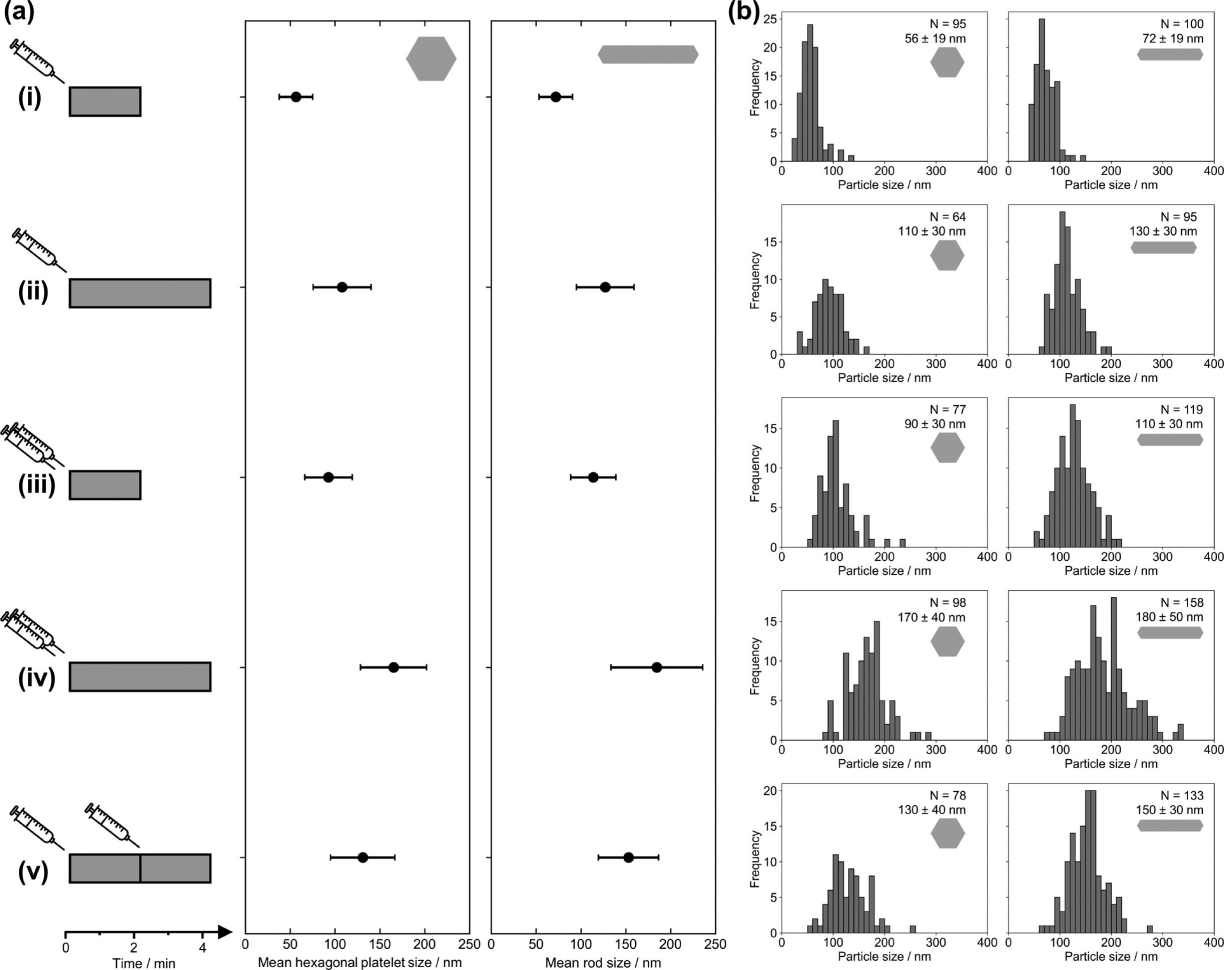
Effect of MgBu_2_ concentration on the Mg NP size for reactions with [LiNapht] = 0.32 M at room temperature. (a) Sizes of hexagonal platelet and rod-shaped Mg NPs after MgBu_2_ precursor injection (illustrated by a syringe symbol) and quenching at varying times (depicted by the gray bar): reactions with the injection of (i) 0.8 mL of MgBu_2_ quenched after 2 min; (ii) 0.8 mL of MgBu_2_ quenched after 4 min; (iii) 1.6 mL of MgBu_2_ quenched after 2 min; (iv) 1.6 mL of MgBu_2_ quenched after 4 min; and (v) 0.8 mL of MgBu_2_ followed by further injection of 0.8 mL of MgBu_2_ after 2 min and quenched after a further 2 min. (b) Corresponding size distributions of hexagonal platelet and rod-shaped NPs from measuring *N* NPs from SEM images.

The nucleation and growth mechanism was confirmed experimentally using sequential additions of MgBu_2_ within the first 4 min of the synthesis (mean sizes, standard deviations, and *p*-values reported in Figures 2 and S10, and Tables S4 and S5). When 0.8 mL of MgBu_2_ was injected to give [MgBu_2_] = 0.065 M in the reaction, increasing the reaction time from 2 min (i) to 4 min (ii) led to larger NPs, with a broader size distribution, suggesting that the nucleation and growth steps overlap. The same result was observed for an initial MgBu_2_ injection of 1.6 mL ([MgBu_2_] = 0.122 M, (iii) and (iv)). The nucleation and growth rates increased with increasing MgBu_2_ concentration, leading to larger NPs. Finally, two sequential additions of 0.8 mL of MgBu_2_ initially and after 2 min of reaction, to give a total [MgBu_2_] = 0.122 M (v), led to slightly larger NPs than a single addition with half concentration (ii) but smaller NPs than a single injection with the same final [MgBu_2_] (iv). The narrower, unimodal distribution suggests that heterogeneous nucleation on the surface of already formed nuclei is favored against homogeneous nucleation.

For all syntheses reported in this paper, the size polydispersities of both hexagonal platelets and rods measured separately varied between 20 and 50% with no obvious trends. The polydispersity arises mainly due to nucleation occurring over an extended period of time that overlaps with NP growth as mentioned above; decoupling of Mg NP nucleation and growth is expected to narrow the size distribution considerably as previously shown in other colloidal syntheses;^29,50^ however, complete decoupling of both stages has not yet been achieved for Mg NP colloidal synthesis.

**Figure 3:**
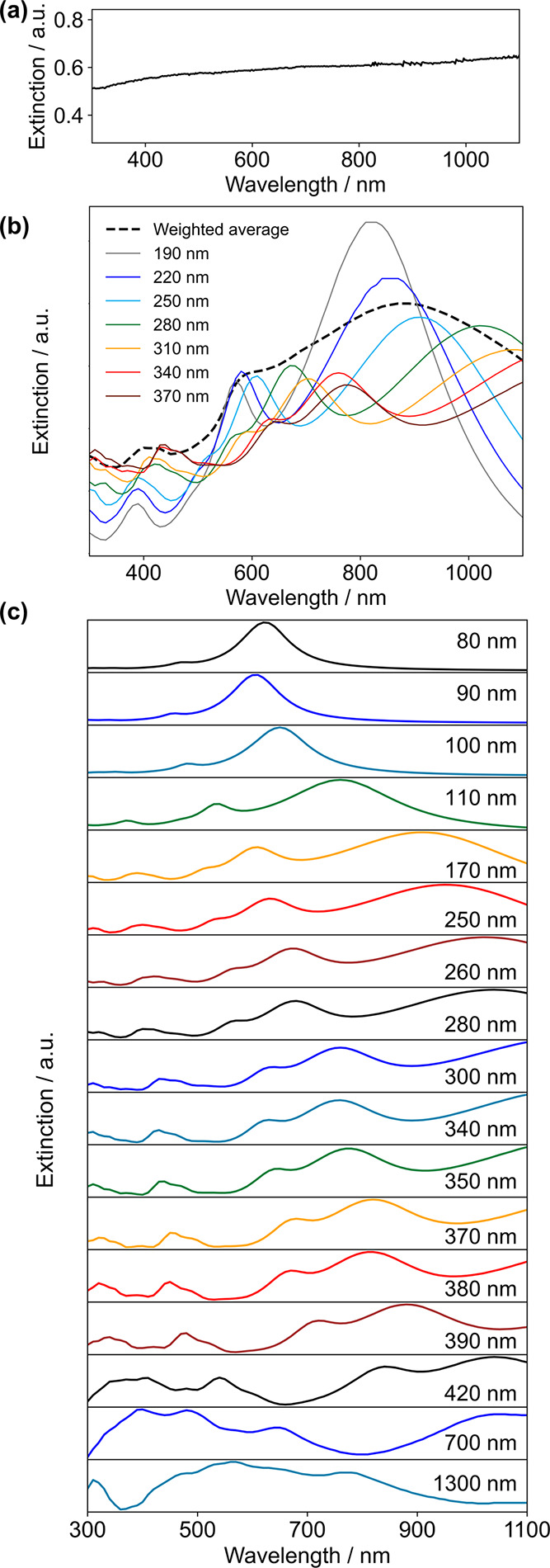
Optical behavior of Mg NPs. (a) Experimental extinction spectrum of Mg NPs from a synthesis with [MgBu_2_] = 0.14 M, with reagent ratios Li/Napht/MgBu_2_ = 2.3:2.3:1, and with a reaction volume of 12.5 mL in a 25 mL flask, dispersed in IPA (average NP size = 300 ± 60 nm). (b) Numerical (discrete dipole approximation in DDSCAT)^44,45^ extinction spectra of hexagonal platelets of sizes found in the synthesis in (a), with platelet thicknesses ∼0.1 times the tip-to-tip length. The black dashed line shows a weighted average using the proportions of each size from the experimental size distribution (Figure S7 reaction 1). (c) Numerical extinction spectra of hexagonal platelets with each of the mean NP sizes reported in this paper.

The broad polydispersity obscures potential differences in the extinction spectra of samples with different sizes since the LSPRs of NPs display multiple modes and even small size variations can lead to large LSPR shifts. As a result, while individual NP spectra show a large shift in LSPR frequencies with NP size (as observed in the dark-field scattering spectra of single Mg hexagonal platelets, Figures S11 and S12), the experimental extinction spectra show no diagnostic difference between samples with different mean sizes (Figures 3a and S13). The broadness of the spectra is consistent with numerical results: a weighted average of seven extinction spectra modelled using the discrete dipole approximation for hexagonal platelets with representative sizes and constant aspect ratio produced a broad spectrum comparable to those seen experimentally (Figure 3b). Additional broadening is caused by the variety of NP shapes present and some aggregation in the sample (since stabilizing ligands are not used). The surface oxide layer, which has previously been shown to be less than 10–20 nm, is expected to cause little broadening of the spectra of individual NPs (modelled in Figure S14) but to introduce further polydispersity in the size of the Mg core for NPs of the same measured size and differences in the dielectric environment. Extinction spectra, nevertheless, are acutely dependent on NP size, as shown for the mean NP sizes reported in this paper (Figure 3c). For these calculations, shapes were generated using the Crystal Creator software,^48^ the frequency-dependent refractive index of metallic Mg was taken from Palik,^46^ and the surrounding environment refractive index was 1.3776, corresponding to IPA.^47^ The interdipole distance was held constant at 0.8 nm for sizes 80–250 nm, 2 nm for sizes of 250–420 nm, and 5 nm for sizes of 700 and 1300 nm; the results are the same using an interdipole distance of 0.8 and 2 nm for a 250 nm NP and using 2 and 5 nm for a 550 nm NP (Figure S15). These results demonstrate the tunability of the Mg plasmon with size, for instance, the dipole resonance moves from 580 nm for 80 nm NPs to over 1100 nm above NP sizes of 300 nm. Furthermore, higher-order modes populate the visible region for all sizes.

### Reaction Time

**Figure 4:**
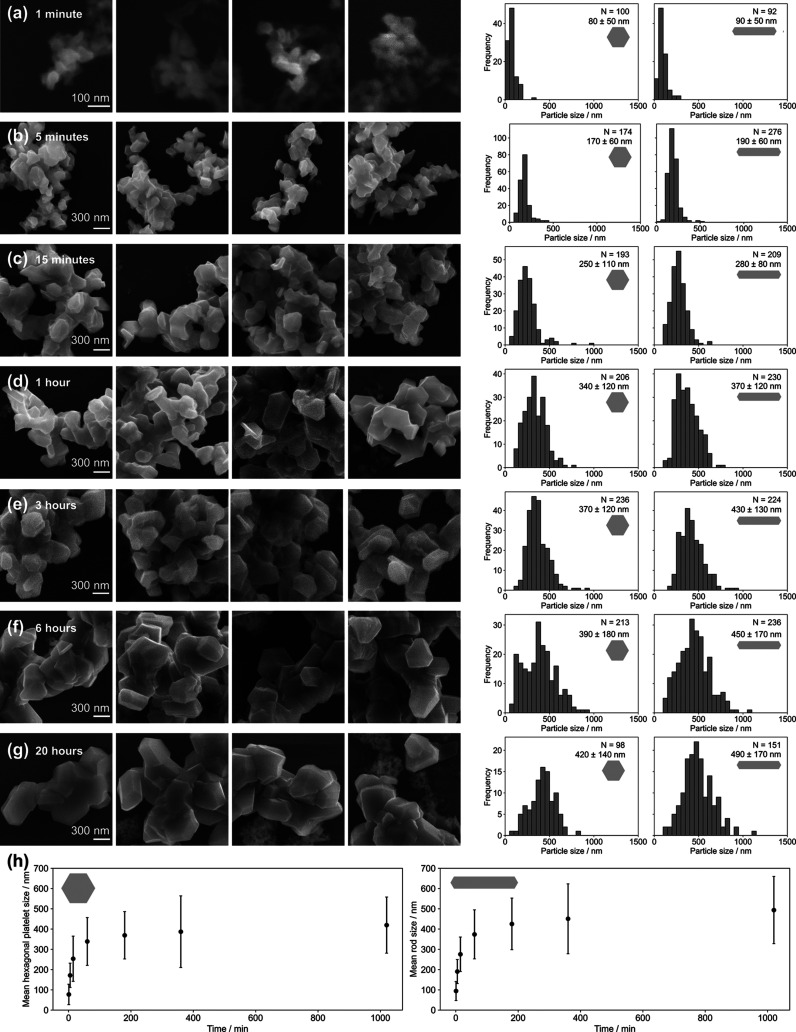
Effect of reaction time on Mg NP synthesis, with Li/naphthalene/MgBu_2_ constant at 2.3:2.3:1 and [MgBu_2_] = 0.14 M at room temperature. (a–g) Representative SEM images and size distributions from reactions quenched after 1 min, 5 min, 15 min, 1 h, 3 h, 6 h, and 20 h. The scale bar is the same for (b–g). (h) Effect of reaction time on the mean sizes of (left) hexagonal platelet and (right) rod-shaped NPs; error bars report standard deviation.

Quenching identical reactions after different reaction times showed that after only 1 min, 80 ± 50 nm hexagonal platelets had already formed. NPs continued to grow rapidly for an hour to form 340 ± 120 nm hexagonal platelets, after which the growth rate decreased. The change in hexagonal platelet size was statistically significant up to 3 h, after which they were 370 ± 120 nm (Figure 4; Tables S6 and S7); after a total reaction time of 20 h, hexagonal platelets reached 420 ± 140 nm. The sizes of rod-shaped NPs follow the same trend, being 90 ± 50 nm after 1 min, 370 ± 120 nm after 1 h, 430 ± 130 nm after 3 h, and 490 ± 170 nm after 20 h. The aspect ratios of rods remained constant at the 95% confidence level, being between 1.9 and 2.0 after 1 min (Figure S16 and Table S8).

While STEM energy-dispersive spectroscopy (STEM-EDS) and the extinction spectrum (Figure S13a) of NPs formed after 1 min showed evidence of significant oxidation of NPs, the morphologies were as expected for metallic Mg, implying that metallic Mg NPs formed before oxidizing. Thus, NP sizes could still be measured. The syntheses discussed hereafter were quenched after 17 h, allowing the final sizes of each system to be reached and compared. As a result, the effects of growth rate are not important, and the overall growth is considered instead, which is determined by the amount of precursors available and coordination of other species to the NP surfaces.

### Reaction Scale-Up

Scaling up the reaction volume in a larger flask decreases the mixing efficiency of magnetic stirring, resulting in localized concentration profiles in the reaction flask after MgBu_2_ injection. As a result, the rate of nucleation decreases, forming fewer nuclei. As NP growth was allowed to occur over 17 h, scaling up the reaction resulted in larger NPs as more Mg precursor was available for growth. Increasing the reaction volume from 12.5 mL (in a 25 mL flask) to 25 mL (in a 50 mL flask) and 50 mL (in a 100 mL flask) with precursor concentrations remaining constant led to an increase in both hexagonal platelet sizes, which were 280 ± 70, 340 ± 110, and 440 ± 100 nm, respectively, and rod-shaped NP sizes, which were 340 ± 110, 400 ± 200, and 490 ± 120 nm (Figures S17 and S18; Tables S9 and S10). The standard deviations showed no trend, being 25, 33, and 22% for 12.5, 25, and 50 mL reactions, respectively.

### Overall Reaction Concentration

To understand the effect of the overall concentration of all precursors in the reaction, we modified the amounts of all reagents, keeping their ratios and the reaction volume constant. Increasing the reaction concentration increases the rate of nucleation, producing more nuclei while simultaneously increasing the amount of Mg available to grow the nuclei.

**Figure 5:**
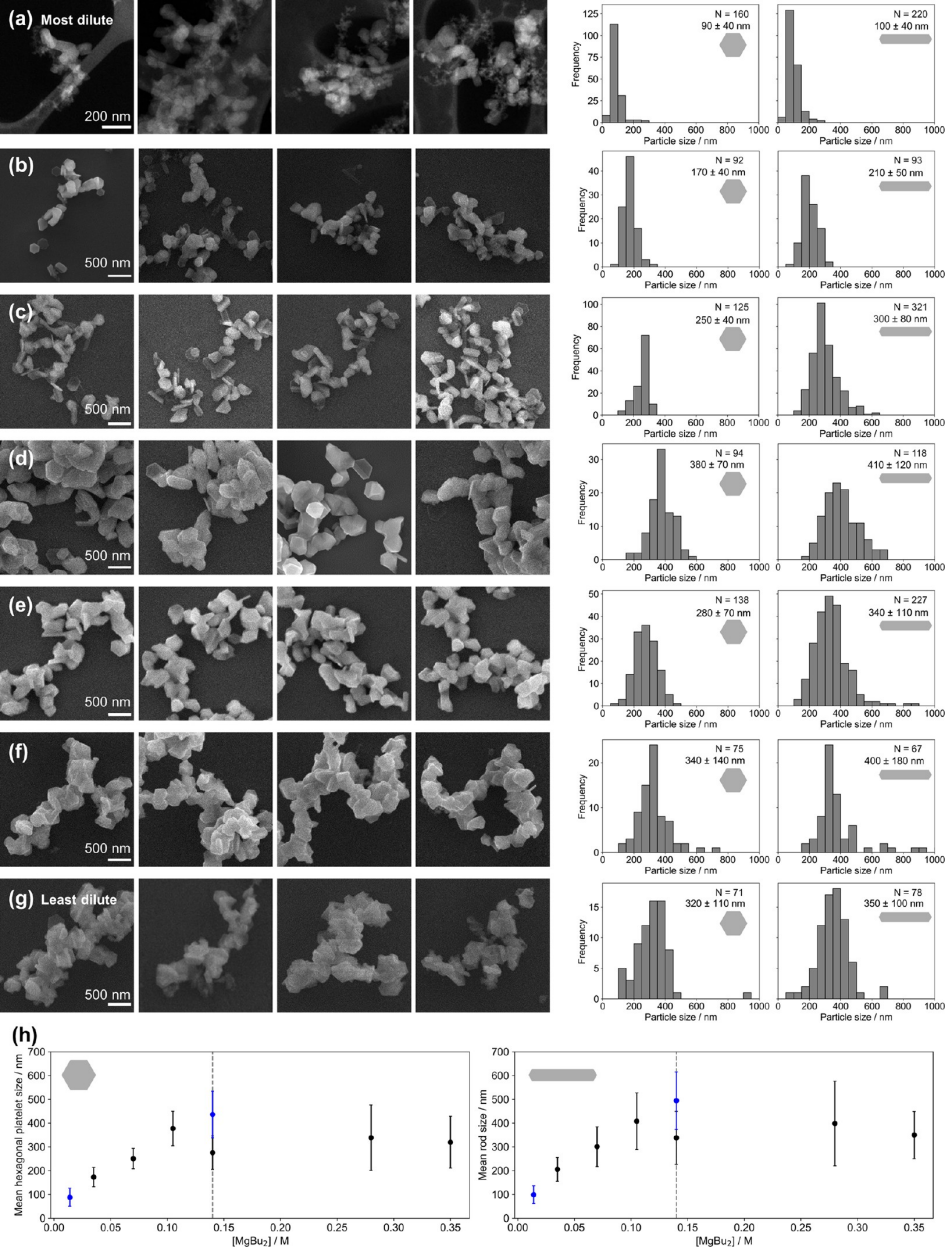
Effect of overall reaction concentrations, keeping the same molar ratio of Li/naphthalene/MgBu_2_ of 2.3:2.3:1 at room temperature. (a) Representative HAADF-STEM images and size distribution from a reaction at [MgBu_2_] = 0.014 M with reaction volume 50 mL. Representative SEM images and size distributions from reactions with volume 12.5 mL at [MgBu_2_] = (b) 0.035 M, (c) 0.070 M, (d) 0.105 M, (e) 0.140 M, (f) 0.280 M, and (g) 0.350 M. The scale bars are the same for (b–g). (h) Effect of reaction concentration quantified by [MgBu_2_] with reaction volume = 12.5 mL in black and 50 mL in blue, on the mean sizes of (left) hexagonal platelet and (right) rod-shaped NPs; error bars report standard deviation and the dashed line marks the standard reaction concentration (0.14 M MgBu_2_).

We found that the effects on the NP growth, which occurs over an extended time and so is not limited by rate, were greater than effects on the nucleation rate, leading to larger NPs. Increasing the overall reaction concentration by varying [MgBu_2_], [Li] and [naphthalene] in constant proportions (1:2.3:2.3 in 12.5 mL of reaction volume) produced an increase in Mg NP size from 90 ± 40 nm for hexagonal platelets and 100 ± 40 nm for rods in the most dilute reaction (0.014 M MgBu_2_) to 380 ± 70 nm hexagonal platelets and 410 ± 120 nm rods at 0.1 M MgBu_2_ (Figure 5). Above this concentration, the trend plateaued and NP sizes became statistically the same (Tables S11 and S12). NP thickness followed the same trend, being 24 ± 9 nm for the smallest NPs and increasing to 73 ± 18 nm at 0.1 M MgBu_2_ before remaining roughly constant (Figure S19a), giving an essentially constant hexagonal platelet aspect ratio of 4.2 ± 0.7 as expected for similar growth conditions.

As the amount of all precursors (overall concentration) increases, overall growth increases, thereby forming NPs with larger sizes. However, at concentrations above ∼0.1 M MgBu_2_, the resulting NP size plateaued at ∼400 nm, likely because nucleation takes place over a longer period of time. It is important to highlight that larger NP sizes are achieved at lower temperatures and, therefore, the size is not thermodynamically limited to ∼400 nm. The increased growth is then offset by the increased number of nuclei, causing the plateau in NP size observed. This explanation is supported by the concentration of nuclei formed in the reaction (Figure S19b), which was estimated using the reaction yield (measured by ICP-MS, Figure S6b) and the average hexagonal platelet size (Figures 5 and S19a); NP concentration remained approximately constant initially and then increased for the highest concentrations.

At the extremes of reaction concentration, experimental challenges arose. In very dilute reactions, minuscule amounts of product were formed, so the most dilute reaction in Figure 5a (MgBu_2_ = [0.014 M]) was performed in a larger (50 mL) reaction volume and compared with a standard concentration reaction ([MgBu_2_] = 0.14 M) at the same volume (Figure 5h). NPs from an equivalent reaction with a 12.5 mL of volume are expected to be smaller than those produced in the 50 mL reaction, in line with the trends already reported with less efficient mixing when scaling up the reaction volume. As the concentration increased above 0.3 M MgBu_2_, quenching the reaction produced significant heat and formed a gel-like byproduct, likely THF decomposed by BuLi, from which Mg NPs could eventually be removed; above 0.35 M MgBu_2_, the gel was too thick to recover any NPs.

### Temperature

**Figure 6:**
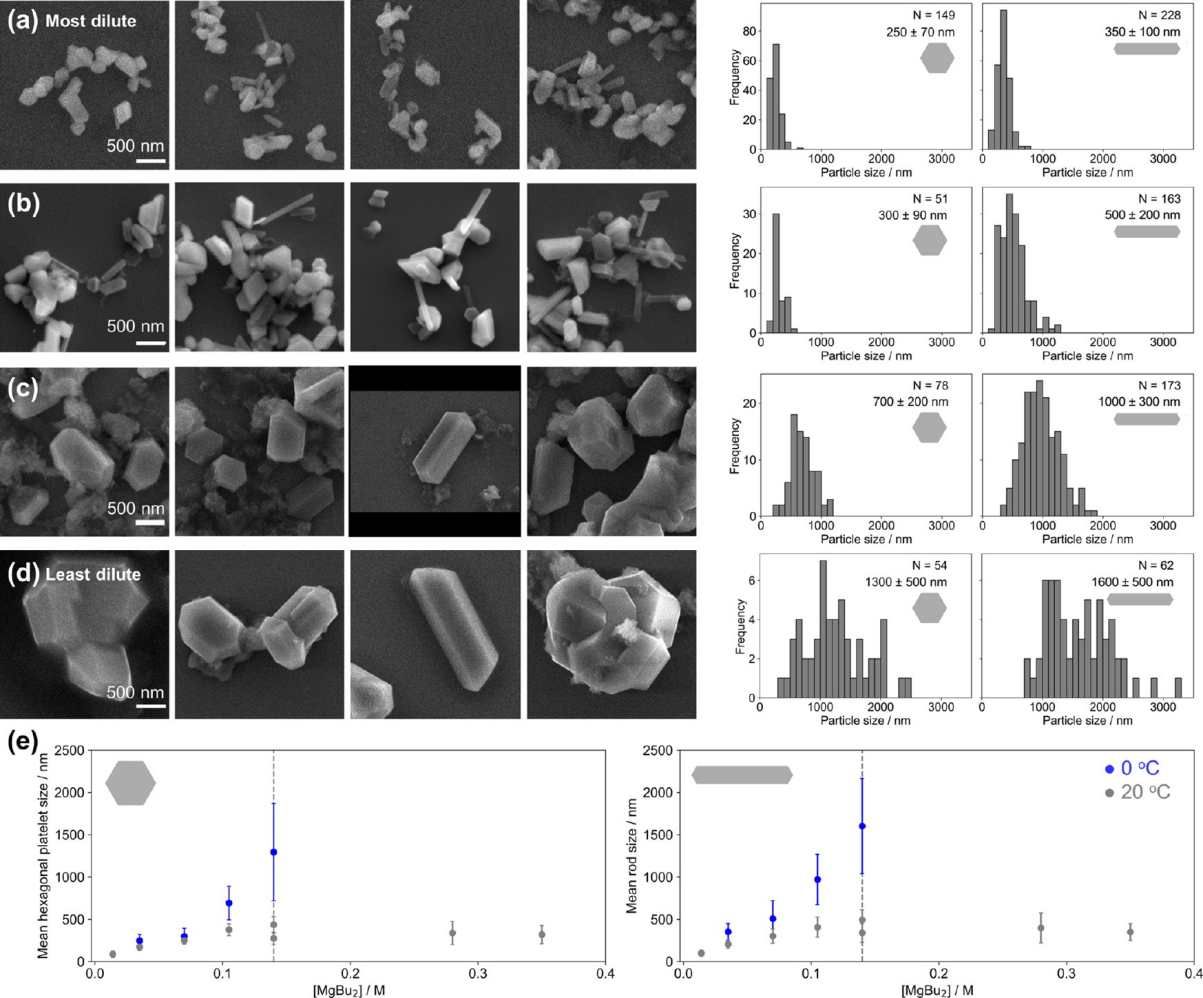
Mg NPs and particles synthesized at different overall reaction concentrations, keeping the same reagent ratios of 2.3:2.3:1 Li/electron carrier/MgBu_2_, at 0 °C. Representative SEM images, on the same scale, and size distributions from reactions at [MgBu_2_] = (a) 0.035 M, (b) 0.070 M, (c) 0.105 M and (d) 0.140 M. (e) Effect of overall reaction concentration quantified by [MgBu_2_] at both room temperature (gray) and 0 °C (blue) on the mean sizes of (left) hexagonal platelets and (right) rod-shaped particles; error bars report standard deviation.

Temperature affects reaction rates exponentially and, therefore, NP nucleation and growth rates.^51−54^ Decreasing the reaction temperature decreases the reduction rate, reducing the nucleation rate and, therefore, producing fewer nuclei. Although the growth rate also decreased, the reaction was again run for long enough for the particles to complete their growth with the remaining Mg^0^, resulting in larger particle sizes. In reactions performed at 0 °C, particles reached the significantly larger sizes of 1300 ± 500 nm for hexagonal platelets and 1300 ± 600 nm for rod-shaped particles at standard reaction concentration ([MgBu_2_] = 0.140 M, Figure 6d). The particles were thicker than those from room temperature syntheses, with additional {101̅1} facets appearing. Intertwinning, as found in minerals like quartz,^55^ was also frequently observed.

Combining temperature-driven control with the effects of overall reaction concentration increased the range of obtainable NP sizes (Figure 6; Tables S13 and S14). The temperature effect is most pronounced for concentrated reactions; in the most dilute reactions, the reduction of nucleation rate is less pronounced since nucleation is already slow, producing 250 ± 70 nm hexagonal platelets compared with 170 ± 40 nm at the same concentration at room temperature and 350 ± 100 nm rods compared with 200 ± 50 nm. However, further increases in NP sizes were not possible. At 0.14 M MgBu_2_, significantly less product was obtained (Figure S1c), and above this concentration, a gel, likely THF decomposed by BuLi, formed upon quenching the reaction from which NPs could not be recovered.

Surprisingly, increasing the reaction temperature to 40 °C had little effect on NP size, at both 0.140 M (Figures S20 and S21; Tables S15 and S16) and 0.035 M MgBu_2_ (Figures S20 and S22; Tables S17 and S18) with LiNapht/MgBu_2_ constant at 2.3:1. Further increases in temperature are limited by THF’s boiling point of 65 °C.^47^

### Electron Carrier

**Figure 7:**
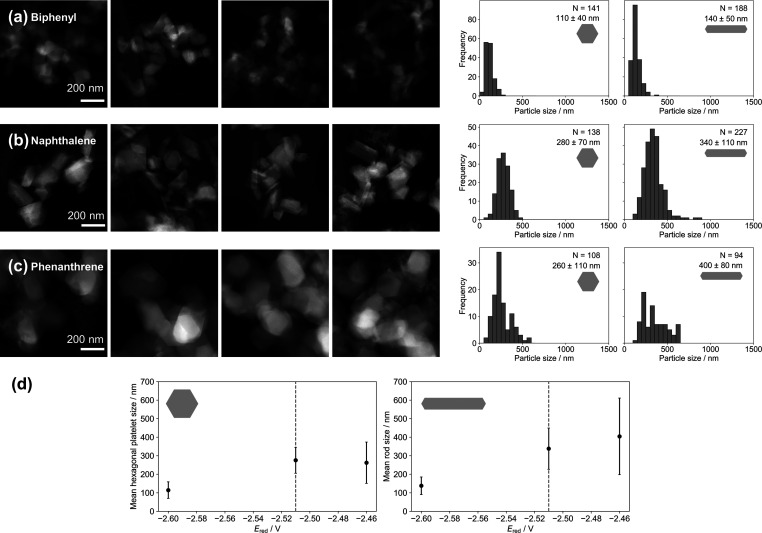
Effect of different electron carriers with varying reduction potentials on Mg NP size. Representative HAADF-STEM images and size distributions from reactions using (a) biphenyl, (b) naphthalene, and (c) phenanthrene, with Li/electron carrier/MgBu_2_ constant at 2.3:2.3:1, [MgBu_2_] = 0.14 M at room temperature. (d) Effect of the reduction potential of the electron carrier on the mean sizes of (left) hexagonal platelets and (right) rod-shaped NPs; error bars report standard deviation.

The reduction potential of the naphthalenide anion (*E*_red_ = −2.51 V)^56,57^ is, and must be, more negative than that of Mg^2+^ (*E*_red_ = −2.37 V);^47^ however, other electron carriers meeting this criterion can be used. Changing the electron carrier significantly alters the reduction potential, resulting in large changes in NP size. The reduction potential of organic radical anions depends on their energy levels. Here, we investigated the effects of replacing naphthalene as an electron carrier (with *E*_red_ = −2.51 V for its radical anion in THF) with biphenyl (*E*_red_ = −2.60 V), phenanthrene (*E*_red_ = −2.46 V), and anthracene (*E*_red_ = −1.96 V).^56,57^ As the reduction potential becomes more negative, the nucleation rate increases, producing more nuclei that grow with the remaining Mg (over an extended time period) to smaller NPs. Using biphenyl produced significantly smaller hexagonal platelets of 110 ± 40 nm and rods of 140 ± 50 nm, while phenanthrene produced similarly sized hexagonal platelets of 260 ± 110 nm and rods of 400 ± 200 nm, compared with naphthalene (280 ± 70 nm hexagonal platelets and 340 ± 110 nm rods) (Figure 7; Tables S19 and S20). The trend in NP sizes is in agreement with Norberg et al., who produced Mg NPs by the reduction of magnesocene (MgCp_2_) with biphenyl, naphthalene, and phenanthrene, although they varied the concentration of MgCp_2_ simultaneously.^34^ We further note that the reduction potential of anthracene is more positive than that of Mg^2+^ so faceted Mg NPs did not form, although Mg-containing nanostructures, not necessarily metallic, were observed by SEM.

Although Mg NP sizes were controllable via the reaction temperature and overall concentration when using naphthalene, these parameters did not affect NP size when using biphenyl as the electron carrier (Figures S23–S26; Tables S11–S24). The large negative reduction potential of the biphenyl anion causes a rapid reduction of MgBu_2_ that is impacted less by further, smaller changes to the nucleation and growth rates. Indeed, in other metal NP syntheses, seed-mediated approaches have been shown to control size using weaker reducing agents for the growth stage after seed (i.e., nuclei) formation, to avoid secondary nucleation.^58,59^

### Additives

**Figure 8:**
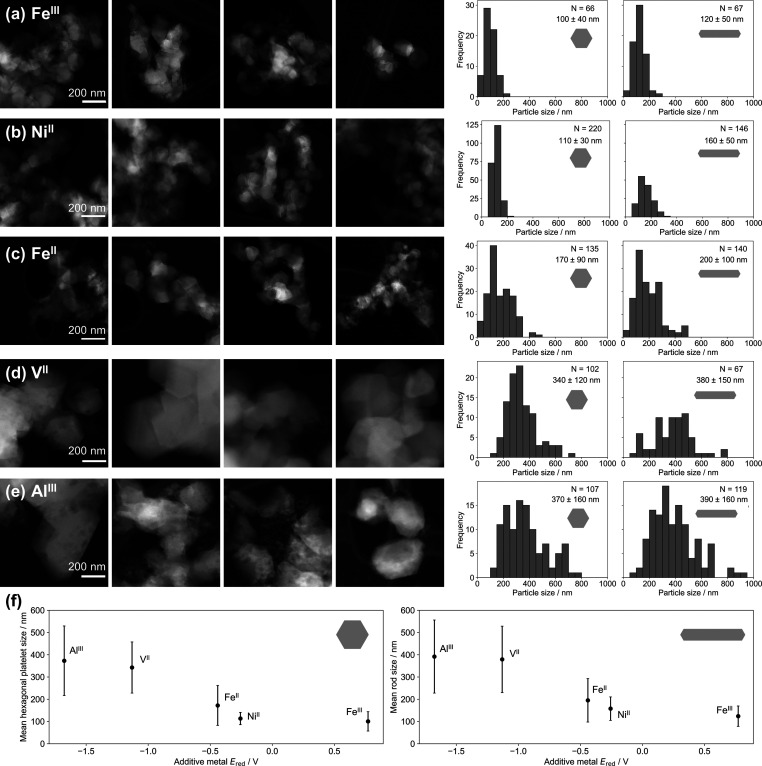
Mg NP size control with metal salt additives of different reduction potentials. Representative HAADF-STEM images and size distributions from reactions with Li/naphthalene/MgBu_2_/metal salt additive constant at 2.3:2.3:1:0.05, [MgBu_2_] = 0.14 M at room temperature in the presence of (a) FeCl_3_, (b) NiCl_2_, (c) FeCl_2_, (d) VCl_2_, and (e) AlCl_3_. (f) Effect of additive reduction potential on the mean sizes of (left) hexagonal platelets and (right) rod-shaped NPs; error bars report standard deviation.

Salt additives are frequently used to control metal NP size and morphology through a variety of mechanisms. For example, in the syntheses of Pd NPs, the addition of Fe^III^ introduces a competing redox reaction. Fe^III^ is reduced to Fe^II^ while Pd^0^ is oxidized back to Pd^II^, thus reducing the overall reduction rate of Pd^II^, leading to a decrease in both nucleation density and growth rate.^60^ Furthermore, ionic additives may preferentially bind to certain facets of the growing crystal, slowing growth on these facets and allowing control over morphology.^61^ In addition, metal salt additives have been shown to affect NP size in Mg syntheses.^62−65^ Therefore, it was hypothesized that the reduction potential of metal ion additives would affect final Mg NP size. We investigated the effects of chloride salts of Fe^III^, Ni^II^, Fe^II^, V^II^, and Al^III^ (*E*_red_ = 0.771, −0.257, −0.44, −1.13, and −1.676 V, respectively)^47^ in a 1:20 molar ratio with Mg and found that the size of Mg NPs decreased as the reduction potential of the additive metal ion increased (Figure 8; Tables S25−S27). Small hexagonal platelet Mg NPs of 100 ± 40 nm and rods of 120 ± 50 nm were formed in the presence of FeCl_3_ (the additive with the most positive reduction potential), while with AlCl_3_, the additive with the most negative reduction potential, large NPs with 370 ± 160 nm hexagonal platelets and 390 ± 160 nm rods were produced, similar to those from an equivalent synthesis in the absence of additives (350 ± 110 nm hexagonal platelets and 400 ± 140 nm rods). With Ni^II^, Fe^II^, and V^II^ (*E*_red_ = −0.257, −0.44, and −1.13 V, respectively), intermediate NP sizes were produced, of 110 ± 30, 170 ± 90, and 340 ± 120 nm for hexagonal platelets, respectively, and 160 ± 50, 200 ± 100, and 380 ± 150 nm for rods.

While control over NP formation using additives is typically attributed to a reduced NP growth rate via additive metal adsorption on the NP surface, reduction retardation^60,66−69^ or a reduction in nucleation density, a different mechanism is at play here: the metal salts affect the nucleation of Mg NPs rather than their growth. Injecting the FeCl_3_ or AlCl_3_ additives 5 min after MgBu_2_ injection, i.e., after nucleation but while NPs are still growing, produced no significant change in NP size compared to Mg NP syntheses without additives (Figure S27), ruling out effects on growth. Furthermore, there is no trend between the yield of Mg NPs and the metal additive reduction potential (Figure S6e), implying that the difference in size is not due to differences in the amount of growth that has occurred by the time of quenching.

Furthermore, although the reduction of additive salts is a competing reaction to the reduction of MgBu_2_, using some of the reducing agents, we show in the next section that the amount of reducing agent present does not affect Mg NP sizes, so this parallel reduction does not explain the trend. Further, the amount of metal additive salt is only 1/20th of that of MgBu_2_. Instead, we hypothesize that metal salts with more positive reduction potentials than Mg are preferentially reduced by LiNapht, creating small seeds early in the reaction. These seeds provide sites for heterogeneous nucleation of Mg that, due to the lattice mismatch, grows on the side of the seed and do not form an encapsulated, core–shell structure. Subsequent quenching and purification oxidizes and/or removes small seeds; the resultant Mg NPs showed no evidence of other metals, their oxides, or Mg alloys in XRD (Figure S28) signatures or STEM-EDS (Figures S29 and S30). Elemental analysis using ICP-MS revealed that the Fe and Al contents were the same or less than in samples produced without additives. However, in the cases of Fe^III^ and Ni^II^, a few isolated oxidized Fe or Ni NPs were observed by HAADF-STEM.

The trend in Mg NP sizes produced using additives generally agrees with the sizes reported by Liu et al.^62−65^ However, we observe none of the homogeneous decoration claimed by these authors (Figures S28–S30), and a closer look at the data shown in refs (62−65) reveals, consistent with our findings, no peaks other than from metallic Mg in the XRD results, nor strong signal in the STEM-EDS for other metals.

### Metal Precursor/Reducing Agent Ratio

Although many NP syntheses can be controlled by the ratio of the metal precursor to the reducing agent,^51,53,70^ we, somewhat surprisingly, found that the reagent ratios had little effect on Mg NP size, as shown from the results obtained when varying only [MgBu_2_] or only [naphthalene] (Figures S31–S40). We hypothesize that the effects on the nucleation rate and overall growth cancel each other out within the concentration ranges studied, resulting in no significant change in NP size both high and low reaction concentrations (Tables S28–S35).

Increasing [MgBu_2_] at constant [LiNapht] increases the nucleation rate, producing more nuclei. The increase in MgBu_2_ offsets both the increased number of NPs to grow, as more nuclei indeed require more precursors to reach an equivalent size, and the increased consumption of Mg^0^ by the increased nucleation. As a result, NP size remained unchanged overall (Figures S31–S34; Tables S28–S31). The range over which varying [MgBu_2_] could be tested was also limited: when [MgBu_2_] was too high (above MgBu_2_/LiNapht = 0.7 at [LiNapht] = 0.32 M and above MgBu_2_/LiNapht = 1.0 at [LiNapht] = 0.08 M), quenching the reaction yielded a gel from which NPs could not be extracted, and when [MgBu_2_] was too low (below MgBu_2_/LiNapht = 0.03 at [LiNapht] = 0.32 M and below MgBu_2_/LiNapht = 0.2 at [LiNapht] = 0.08 M), insufficient product was formed to be recovered.

The reducing agent, i.e., the naphthalenide anion, must be formed by reducing naphthalene with Li. When [Li] < [naphthalene], the amount of reducing agent formed is limited by the Li available, leaving LiNapht/MgBu_2_ constant at 2.3:1 and having no impact on the rates of nucleation or growth. The excess naphthalene remains in solution and binds to NP surfaces, rendering NP purification challenging.

When [naphthalene] < [Li], decreasing [naphthalene] decreases the amount of reducing agent formed. The reduction rate decreased, forming fewer nuclei, but less MgBu_2_ could be reduced, resulting in less overall growth. As a result, NP sizes remained approximately constant both at high and low overall concentrations of [MgBu_2_] = 0.140 and 0.035 M (Figures S35–S40; Tables S32–S35). The observation is in agreement with Locatelli et al., who report no change in Mg NP formation with varying [naphthalene].^35^

### Solvent

Solvents can coordinate to surfaces and affect size for NPs including those of Au and Al.^71−73^ For Mg NP synthesis, the solvent must be aprotic and low polarity to avoid unwanted reactions with the reducing agent: protic or highly polar solvents react with LiNapht, whereas nonpolar solvents cannot stabilize the complex by binding to the alkali metal cation. These requirements leave a limited choice of solvents including low-polarity ether solvents such as THF. We replaced THF with 1,2-dimethoxyethane (glyme), 2-methoxyethyl ether (diglyme), 1,4-dioxane, and dibutyl ether. When the solvent polarity was too low, as in the case of 1,4-dioxane and dibutyl ether, LiNapht could not be stabilized by the solvent and did not form.

With polar enough solvents, increasing the number of oxygen atoms in the solvent molecule increases the coordination with the NP surface. The rates of nucleation and growth are not expected to change. Using glyme, which has two ether groups, produced slighter smaller Mg NPs (210 ± 60 nm hexagonal platelets and 260 ± 70 nm rods) compared to THF (280 ± 70 nm hexagonal platelets and 340 ± 110 nm rods), which has one ether group (Figures S41 and S42; Tables S36 and S37). However, the increased solvent coordination makes NP purification challenging, so this route is not recommended for Mg NP size control. In fact, diglyme, which has three ether groups, could not be removed sufficiently from the surface of NPs by further centrifugation steps using THF, IPA, or ethanol to observe distinct NPs by SEM.

Solvent mixtures can also lead to size control by producing intermediate capping interactions. For Mg, solvent mixtures of glyme or diglyme with THF allowed further size tuning. A 50:50 glyme/THF mixture produced NP sizes between those with only THF or glyme (270 ± 70 nm hexagonal platelets and 310 ± 90 nm rods; Figures S41 and S42; Tables S36 and S37). Using a 50:50 diglyme/THF mixture, solvent removal became easier than with diglyme alone, and significantly smaller NPs were formed (140 ± 70 nm hexagonal platelets and 160 ± 70 nm rods, Figures S41 and S42; Tables S36 and S37). We expect that other mixture ratios will allow continuous size tuning according to this trend, with, as observed, a higher proportion of solvent containing multiple ether groups producing smaller NPs.

## Conclusions

Mg NPs, an earth-abundant and biocompatible alternative to the more expensive plasmonic metals Au and Ag, were synthesized using a facile, one-pot colloidal synthesis in which MgBu_2_ was reduced by an organic electron carrier anion formed by reduction with Li. The reaction was shown to follow a nucleation and growth mechanism, and mean NP sizes were successfully tuned between 80 and 1300 nm by varying the reaction time, overall reaction concentration, temperature, electron carrier, and using metal salt additives to control the NPs’ nucleation and growth. However, the approaches reported here do not allow the separation of the nucleation and growth stages (as is done for seeded growth); thus, the size polydispersities remained broad. The observation of many single nanoparticles as well as the analysis of aggregation patterns, including the observation of rather open aggregates (Figures 7, 8, S39, and S43, for instance) and the lack of systematic orientation relationships within the aggregates, is consistent with single-particle nucleation and growth followed by aggregation in solution or during drying. Some heterogeneous nucleation on already formed particles cannot be ruled out, yet we do not believe that it is a dominant mechanism.

Small NPs of 80 nm were formed by quenching the reaction 1 min after MgBu_2_ injection. If left to react, the NPs continued to grow over 3 h to form 400 nm NPs. Final NP sizes were reduced to ∼100 nm by decreasing the overall reaction concentration, by replacing the naphthalene electron carrier with biphenyl or using the metal salt additives of FeCl_3_ or NiCl_2_. The overall reaction concentration was further used to select particle sizes between 90 and 400 nm at room temperature or between 250 and 1300 nm for reactions at 0 °C. Metal salt additives of FeCl_2,_ VCl_2_, and AlCl_3_ also allowed the selection of Mg NP sizes between 170 and 390 nm.

Although the molar ratio between the metal precursor and the reducing agent is commonly used to control metal NP sizes, it surprisingly had no effect on our Mg NP syntheses. Changing the ether used as a reaction solvent to increase coordination with the NP surfaces decreased the NP sizes; however, this method caused difficulties in purifying NPs and is not recommended. The reaction mixing efficiency was decreased by increasing the reaction scale, resulting in slower nucleation rates that produced larger NPs. Future scale-up of this synthesis will, therefore, require careful consideration of reagent mixing.

Control over NP sizes is critical to manipulating their plasmonic properties; the results presented here provide a synthetic framework for Mg-based nanotechnologies and their many potential applications in photocatalysis, sensing, and medical fields.

## Supporting Information Available

Reaction yield results, reaction reproducibility results, additional SEM and STEM images, size distributions, NP size values, statistical analyses, XRD, and extinction spectra (experimental and modelled) (PDF)
Video of Mg NPs in IPA drying on a glass slide viewed in a dark-field optical microscope in real time (AVI)

## Supplementary Material

jp1c07544_si_001.pdf

jp1c07544_si_002.avi
