# Supplementary material for: Size Control in the Colloidal Synthesis of Plasmonic Magnesium Nanoparticles
Source: J Phys Chem C Nanomater Interfaces. 2021 Dec 28;126(1):563–77. doi: 10.1021/acs.jpcc.1c07544 (PMC8762659; doi:10.1021/acs.jpcc.1c07544)
Supplement: Supplementary file 1 — jp1c07544_si_001.pdf [file jp1c07544_si_001.pdf]

Supplementary Information for

Size Control in the Colloidal Synthesis of Plasmonic Magnesium Nanoparticles

Elizabeth R. Hopper,[1,2,3] Thomas M. R. Wayman,[1,2], Jérémie Asselin,[1,2] Bruno Pinho,[3] Christina Boukouvala,[1,2] Laura Torrente-Murciano[3] & Emilie Ringe[1,2*]

1. Department of Materials Science and Metallurgy, University of Cambridge, 27 Charles Babbage Road, Cambridge, United Kingdom, CB3 0FS
2. Department of Earth Sciences, University of Cambridge, Downing Street, Cambridge, United Kingdom, CB2 3EQ
3. Department of Chemical Engineering and Biotechnology, University of Cambridge, Philippa Fawcett Drive, Cambridge, United Kingdom, CB3 0AS

* Corresponding author: er407@cam.ac.uk; +44 (0)1223 334330 (ph.)

Characterization of Mg NPs

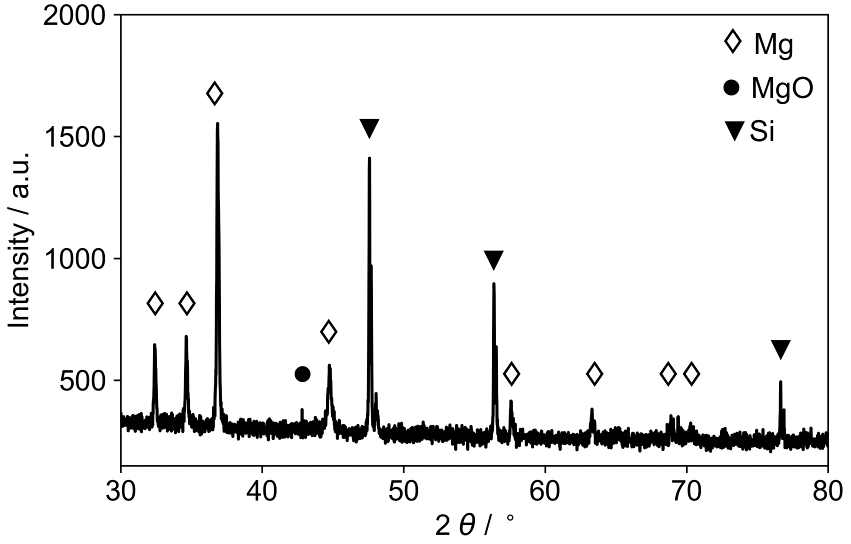

Figure S1. Composition of Mg NPs from a synthesis with [$MgBu_2$] = 0.14 M, with reagent ratios Li:Napht:$MgBu_2$ = 2.3:2.3:1 and with a reaction volume of 12.5 mL in a 25 mL flask at room temperature, measured by XRD.

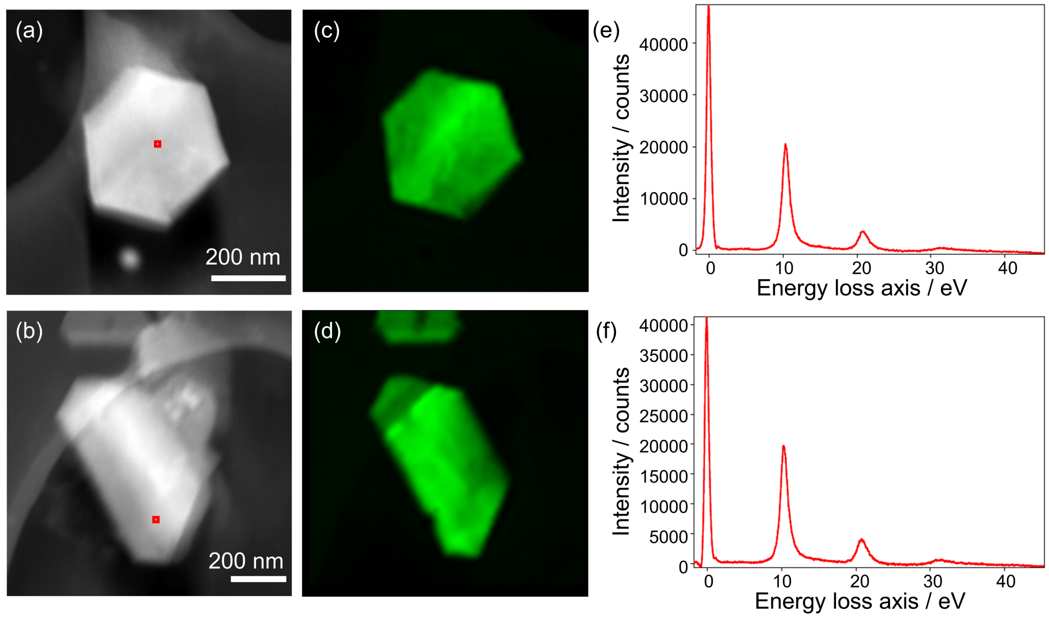

Figure S2. Metallic character of two Mg NPs from a synthesis with [$MgBu_2$] = 0.14 M, with reagent ratios Li:Napht:$MgBu_2$ = 2.3:2.3:1 and with a reaction volume of 12.5 mL in a 25 mL flask at room temperature confirmed by STEM-EELS. (a)-(b) HAADF-STEM images, (c)-(d) maps of the bulk plasmon of Mg at 10.6 eV and (e)-(f) the EELS spectra at the points marked in the HAADF-STEM images with a red box.

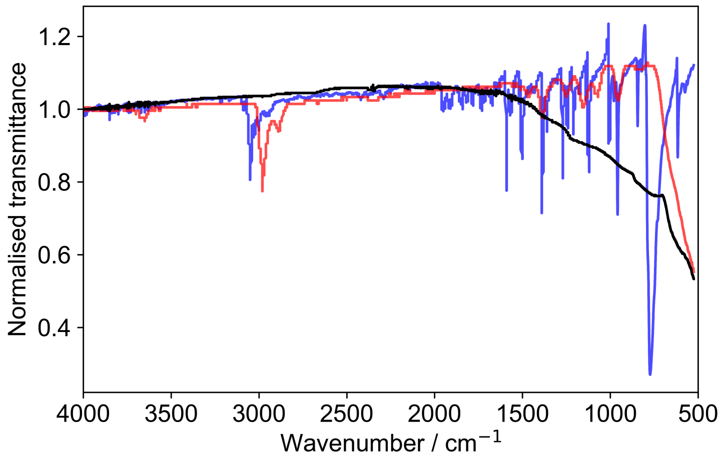

Figure S3. FT-IR spectra of Mg NPs from a synthesis with [$MgBu_2$] = 0.14 M, with reagent ratios Li:Napht:$MgBu_2$ = 2.3:2.3:1 and with a reaction volume of 12.5 mL in a 25 mL flask at room temperature (black), MgO (red) and naphthalene (blue), normalised at 4000 cm^{-1}.

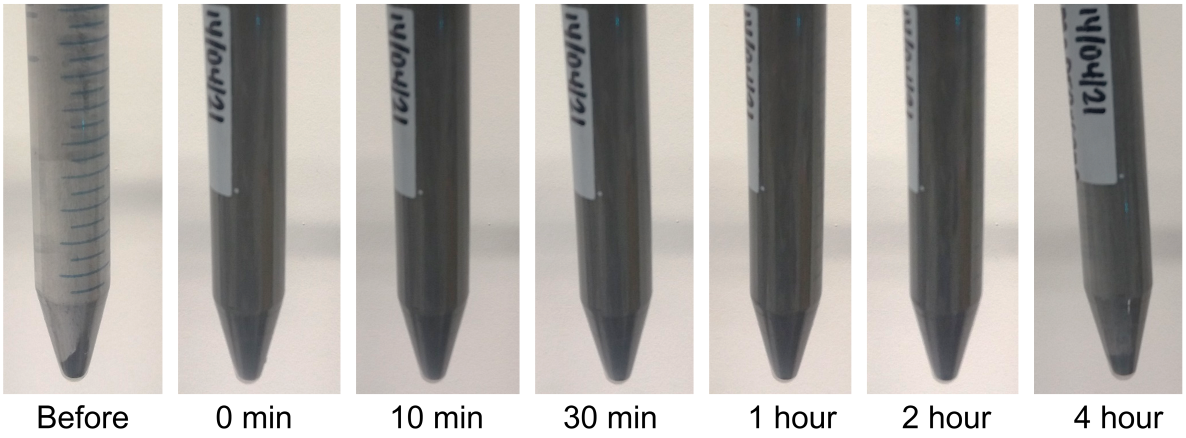

Figure S4. Mg NPs in IPA from a synthesis with [$MgBu_2$] = 0.14 M, with reagent ratios Li:Napht:$MgBu_2$ = 2.3:2.3:1 and with a reaction volume of 12.5 mL in a 25 mL flask at room temperature, before redispersion by sonication (having been left to settle overnight), immediately after sonication and 10 minutes, 30 minutes, one hour, two hours and four hours after sonication. NPs remained suspended in solution for over four hours after sonication, with some product beginning to settle at the bottom of the tube after two hours.

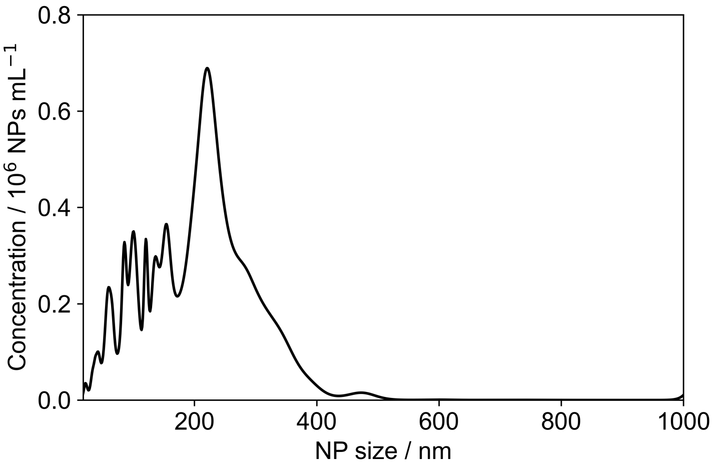

Figure S5. NTA results for a diluted suspension of Mg NPs in IPA from a synthesis with [$MgBu_2$] = 0.14 M, with reagent ratios Li:Napht:$MgBu_2$ = 2.3:2.3:1 and with a reaction volume of 12.5 mL in a 25 mL flask at room temperature. The mean size was 210 nm with a standard deviation of 90 nm and the mode was 220 nm.

Reaction Yields

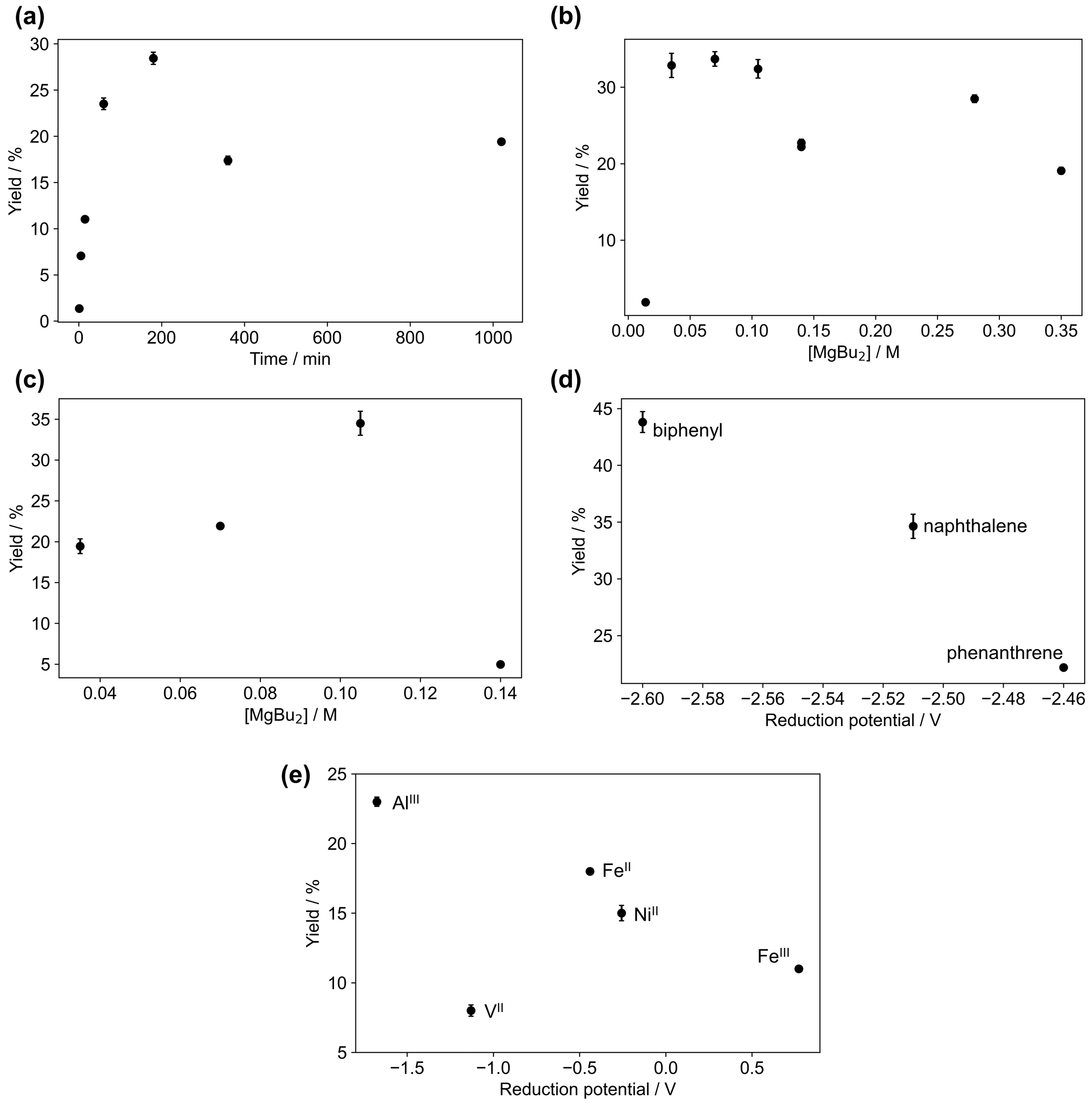

Figure S6. Reaction yield after NP purification with varying (a) reaction time, (b) overall reaction concentration with the same reagent ratios of 2.3:2.3:1 Li:naphthalene:$MgBu_2$, labelled by [$MgBu_2$], (c) overall concentration at low temperature with the same reagent ratios of 2.3:2.3:1 Li:naphthalene:$MgBu_2$, labelled by [$MgBu_2$], (d) reduction potential of the electron carrier and (e) reduction potential of the salt additives. Error bars report standard deviations over five ICP-MS measurements.

Reaction Reproducibility

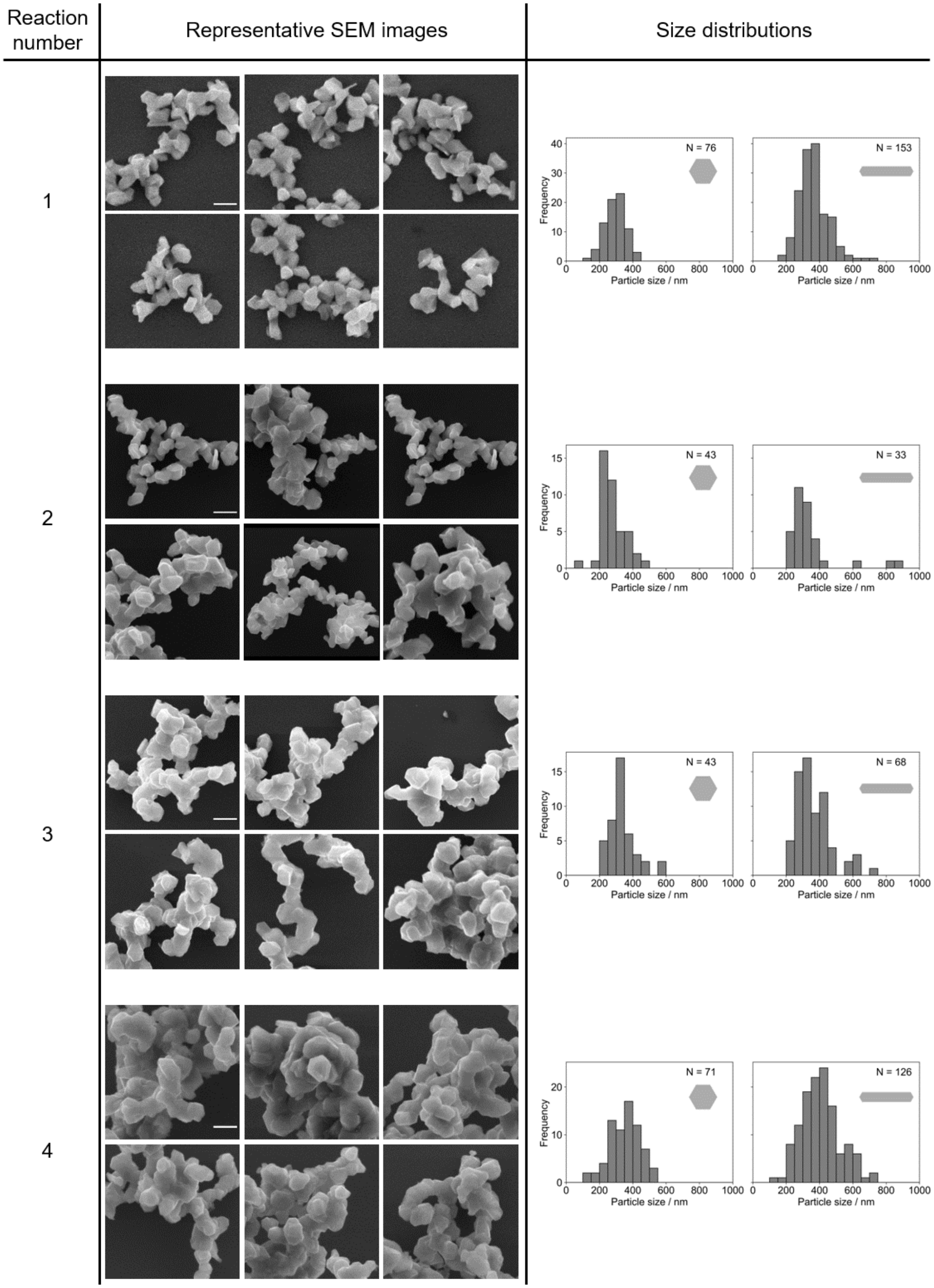

Figure S7. Representative SEM and size distributions of four separate reactions with 2.3:2.3:1 Li:naphthalene:$MgBu_2$, [$MgBu_2$] = 0.14 M and a reaction volume of 12.5 mL in a 25 mL flask at room temperature. Scale bars, 500 nm.

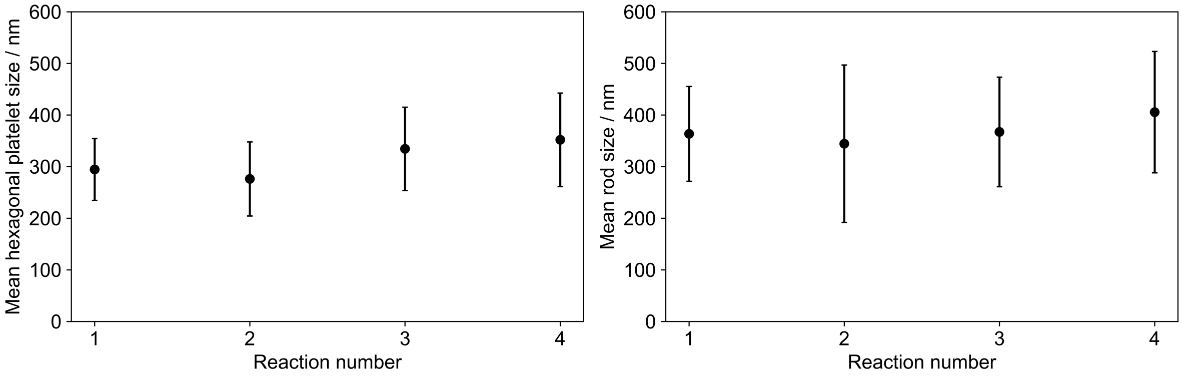

Figure S8. Comparison of hexagonal platelet and rod-shaped NP sizes for the four reactions in Figure S7.

Table S1. Hexagonal platelet sizes (2nd row) for the reactions in Figures S7 and S8 and p-values from Games-Howell pairwise testing that sizes of hexagonal platelets are the same for each pair of reactions. Values are in bold where the two mean sizes are statistically the same.

	Reaction 1	Reaction 2	Reaction 3	Reaction 4
	300 ± 60 nm (20%)	280 ± 70 nm (26%)	330 ± 80 nm (24%)	350 ± 90 nm (26%)
Reaction 1		**0.49**	0.03	0.00
Reaction 2	**0.49**		**0.49**	0.00
Reaction 3	0.03	**0.49**		**0.71**
Reaction 4	0.00	0.00	**0.71**	

Table S2. Rod-shaped NP sizes (2nd row) for the reactions in Figures S7 and S8 and p-values from Games-Howell pairwise testing that sizes of rod-shaped NPs are the same for each pair of reactions. Values are in bold where the two mean sizes are statistically the same.

	Reaction 1	Reaction 2	Reaction 3	Reaction 4
	360 ± 90 nm (25%)	340 ± 150 nm (44%)	370 ± 110 nm (29%)	410 ± 120 nm (29%)
Reaction 1		**0.90**	**0.99**	0.01
Reaction 2	**0.90**		**0.90**	**0.16**
Reaction 3	**0.99**	**0.90**		**0.10**
Reaction 4	0.01	**0.16**	**0.10**	

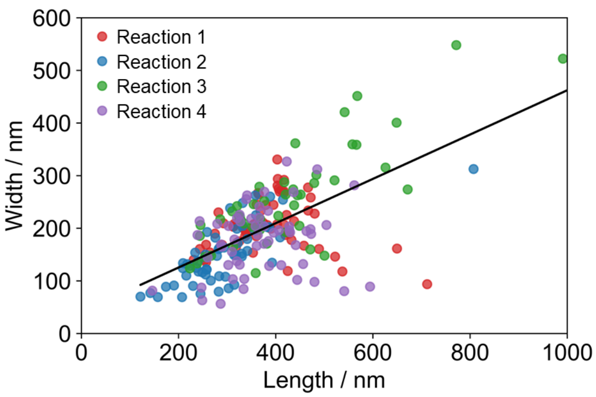

Figure S9. Aspect ratio distributions of rods for the reactions in Figure S7. Average aspect ratios for each reaction are reported in Table S3 and average lengths are reported in Table S2.

Table S3. Aspect ratios (length/width) of rod-shaped NP sizes (2nd row) for the reactions in Figures S7 and S8 (errors report standard deviations) and p-values from Games-Howell pairwise testing that aspect ratios are the same for each pair of reactions. Values are in bold where the two mean sizes are statistically the same.

	Reaction 1	Reaction 2	Reaction 3	Reaction 4
	1.9 ± 1.0	2.1 ± 0.5	1.7 ± 0.5	2.4 ± 1.2
Reaction 1		**0.99**	**0.99**	**0.22**
Reaction 2	**0.99**		**0.06**	**0.30**
Reaction 3	**0.31**	**0.06**		0.00
Reaction 4	**0.22**	**0.30**	0.00	

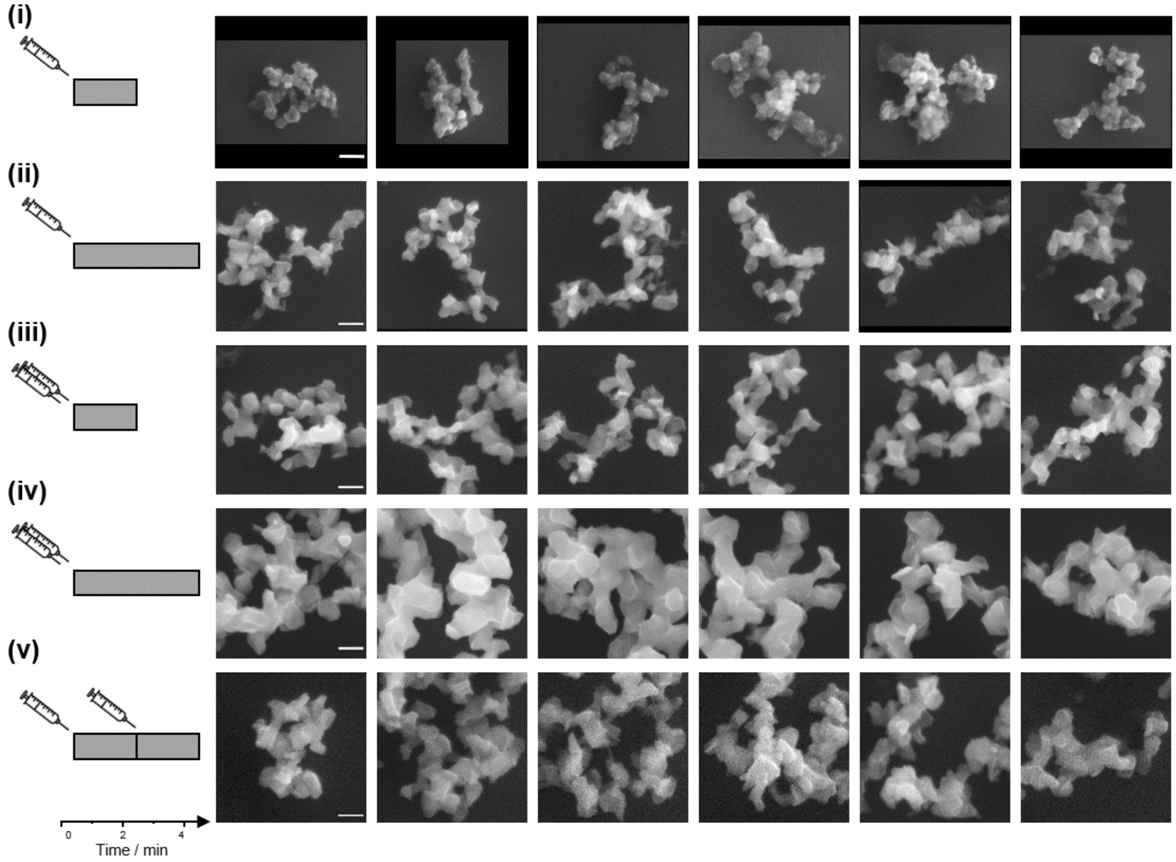

Figure S10. Representative SEM images of NPs after precursor injection and quenching at varying times, from the reactions reported in Figure 2 and following the same addition pattern as described in Figure 2a, *i.e.*, reactions with injection of (i) 0.8 mL $MgBu_2$ quenched after two minutes, (ii) 0.8 mL $MgBu_2$ quenched after four minutes, (iii) 1.6 mL $MgBu_2$ quenched after two minutes, (iv) 1.6 mL $MgBu_2$ quenched after four minutes and (v) 0.8 mL $MgBu_2$ followed by further injection of 0.8 mL $MgBu_2$ after two minutes and quenched after a further two minutes.

Table S4. Hexagonal platelet sizes (2nd row) for reactions in Figure 2 (labelled by location in Figures 2, S10) and p-values from Games-Howell pairwise testing that sizes of hexagonal platelets are the same for each pair of reactions. No two mean sizes were statistically the same.

	i	ii	iii	iv	v
	56 ± 19 nm (33%)	110 ± 30 nm (30%)	90 ± 30 nm (29%)	170 ± 40 nm (22%)	130 ± 40 nm (27%)
i		0.00	0.00	0.00	0.00
ii	0.00		0.00	0.00	0.00
iii	0.00	0.00		0.02	0.00
iv	0.00	0.00	0.02		0.00
v	0.00	0.00	0.00	0.00	

Table S5. Rod-shaped NP sizes (2nd row) for reactions in Figure 2 (labelled by location in Figures 2, S10) and p-values from Games-Howell pairwise testing that sizes of rod-shaped NPs are the same for each pair of reactions. No two mean sizes were statistically the same.

	i	ii	iii	iv	v
	72 ± 19 nm (26%)	130 ± 30 nm (25%)	110 ± 30 nm (22%)	180 ± 50 nm (28%)	150 ± 30 nm (22%)
i		0.00	0.00	0.00	0.00
ii	0.00		0.00	0.00	0.00
iii	0.00	0.00		0.01	0.00
iv	0.00	0.00	0.01		0.00
v	0.00	0.00	0.00	0.00	

Extinction Spectra

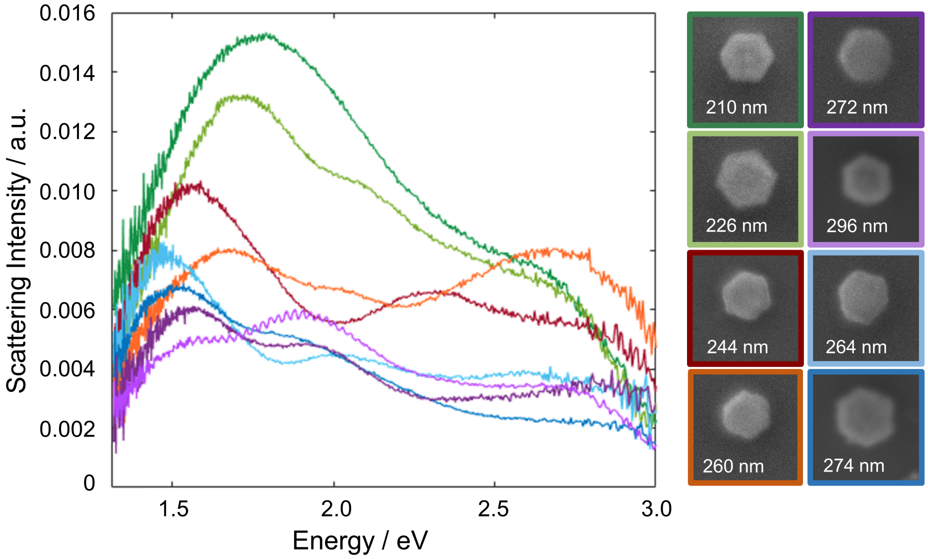

Figure S11. Scattering spectra and corresponding SEM images for Mg hexagonal platelets with varying size. SEM image frame colors match the corresponding scattering profile and the hexagonal platelet size (tip-to-tip) is reported on each image in white.

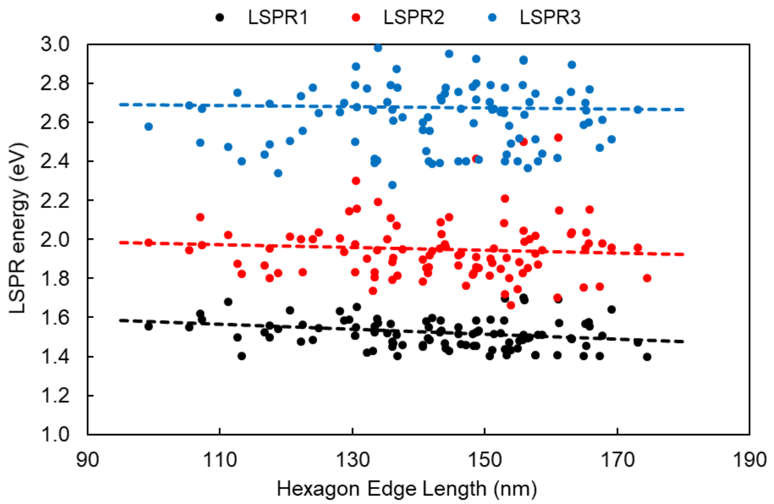

Figure S12. Size dependence of the three detected LSPRs of Mg hexagonal platelets. The fitted lines are based on the coefficients of linear regression analysis. The slopes (95% confidence interval) for LSPR 1, 2 and 3 are −0.0013 eV nm^{-1} (−0.0022 eV nm^{-1} to −0.0004 eV nm^{-1}), −0.0007 eV nm^{-1} (−0.0024 eV nm^{-1} to 0.0011 eV nm^{-1}) and −0.0003 eV nm^{-1} (−0.0035 eV nm^{-1} to 0.0022 eV nm^{-1}).

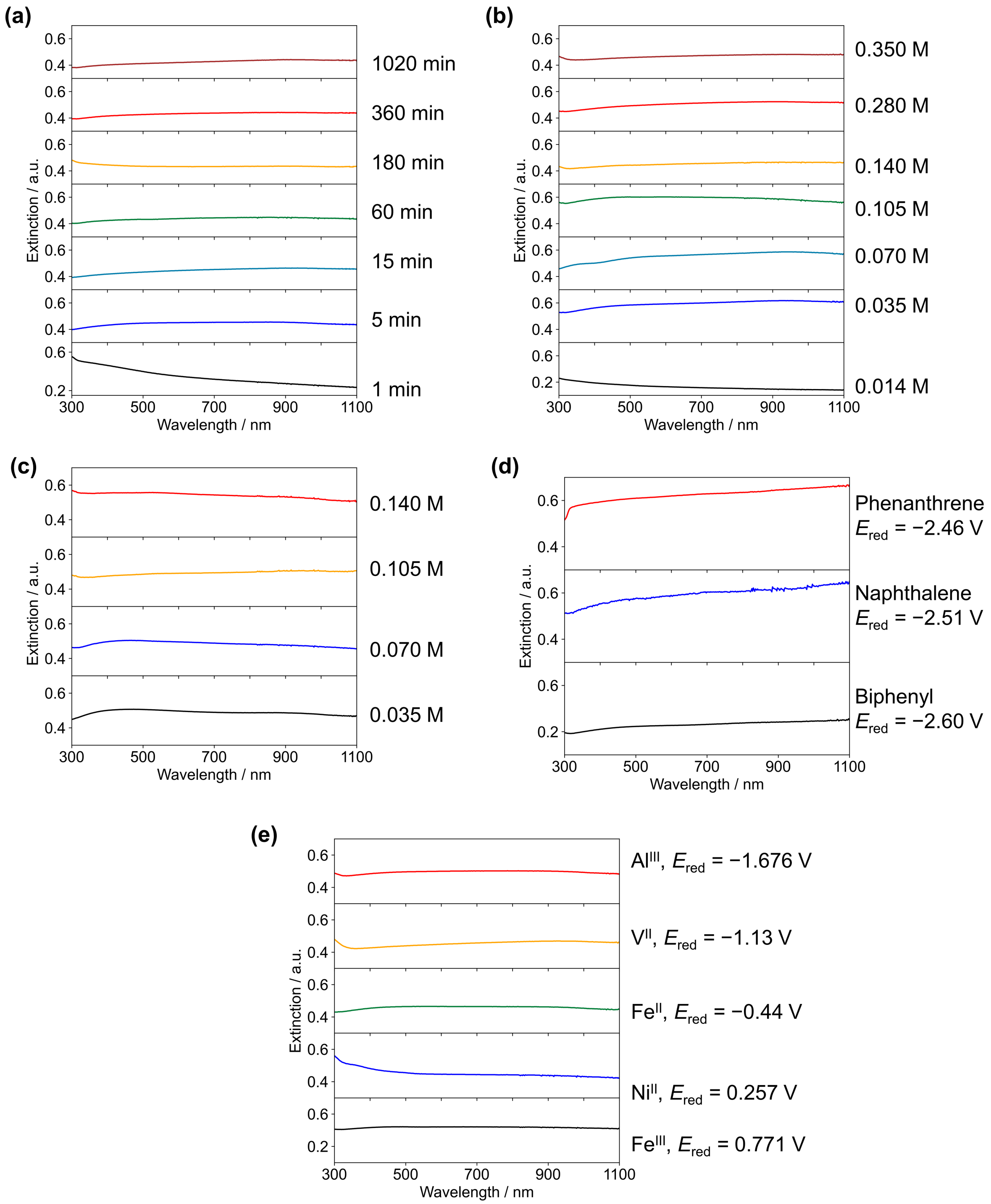

Figure S13. Extinction spectra from samples with varying (a) reaction time, (b) overall reaction concentration, where the legend is [$MgBu_2$], (c) overall concentration at low temperature, where the legend is [$MgBu_2$], (d) electron carrier and (e) salt additives. Samples were diluted to give approximately equal maximum extinctions.

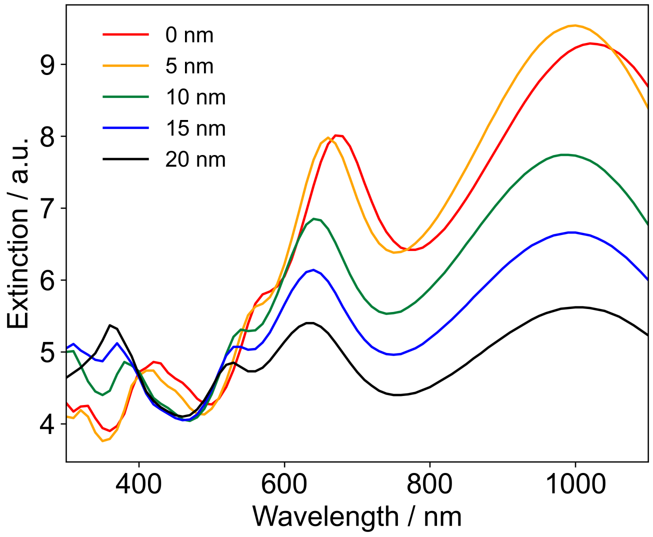

Figure S14. Numerical (discrete dipole approximation in DDSCAT)[1,2] extinction spectra of hexagonal platelets with a total tip-to-tip length of 280 nm, including an oxide layer of 0 nm (red), 5 nm (yellow), 10 nm (green), 15 nm (blue) and 20 nm (black). Shapes were generated using the Crystal Creator software with platelet thicknesses ~0.1 times the tip-to-tip length and an interdipole distance of 2 nm;[3] the frequency-dependent refractive index of metallic Mg was taken from Palik[4] and the surrounding environment refractive index was 1.3776 corresponding to IPA.[5] The shift in peak position with increasing oxide thickness corresponds to a combination of red-shift due to the refractive index of the dielectric environment and blue-shift due to the decreasing size of the Mg core.

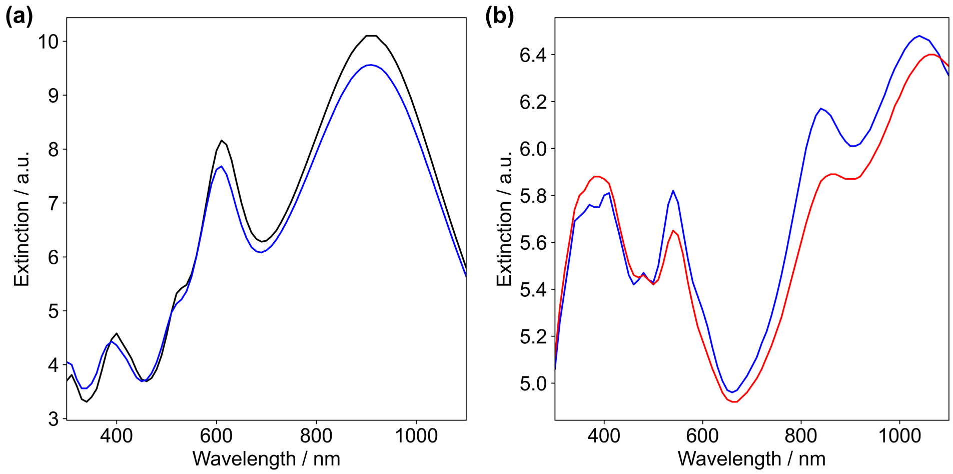

Figure S15. Numerical (discrete dipole approximation in DDSCAT)[1,2] extinction spectra of hexagonal platelets of (a) 250 nm, using interdipole distances 0.8 nm (black) and 2 nm (blue), and (b) 550 nm, using interdipole distances of 2 nm (blue) and 5 nm (red), showing that the peak position does not change significantly upon changing the interdipole distance. Shapes were generated using the Crystal Creator software with platelet thicknesses ~0.1 times the tip-to-tip length;[3] the frequency-dependent refractive index of metallic Mg was taken from Palik[4] and the surrounding environment refractive index was 1.3776 corresponding to IPA.[5]

Reaction Time

Table S6. Hexagonal platelet sizes (2nd row) for reactions lasting varying times (Figure 4) and p-values from Games-Howell pairwise testing that sizes of hexagonal platelets are the same for each pair of reactions. Values are in bold where the two mean sizes are statistically the same.

	1 minute	5 minutes	15 minutes	1 hour	3 hours	6 hours	20 hours
	80 ± 50 nm (65%)	170 ± 60 nm (35%)	250 ± 110 nm (44%)	340 ± 120 nm (35%)	370 ± 120 nm (32%)	390 ± 180 nm (46%)	420 ± 140 nm (33%)
1 minute		0.00	0.00	0.00	0.00	0.00	0.00
5 minutes	0.00		0.00	0.00	0.00	0.00	0.00
15 minutes	0.00	0.00		0.00	0.00	0.00	0.00
1 hour	0.00	0.00	0.00		**0.09**	0.02	0.00
3 hours	0.00	0.00	0.00	**0.09**		**0.88**	0.03
6 hours	0.00	0.00	0.00	0.02	**0.88**		**0.57**
20 hours	0.00	0.00	0.00	0.00	0.03	**0.57**	

Table S7. Rod-shaped NP sizes (2nd row) for reactions lasting varying times (Figure 4) and p-values from Games-Howell pairwise testing that sizes of rod-shaped NPs are the same for each pair of reactions. Values are in bold where the two mean sizes are statistically the same.

	1 minute	5 minutes	15 minutes	1 hour	3 hours	6 hours	20 hours
	90 ± 50 nm (50%)	190 ± 60 nm (31%)	280 ± 80 nm (31%)	370 ± 120 nm (32%)	430 ± 130 nm (30%)	450 ± 170 nm (38%)	490 ± 170 nm (34%)
1 minute		0.00	0.00	0.00	0.00	0.00	0.00
5 minutes	0.00		0.00	0.00	0.00	0.00	0.00
15 minutes	0.00	0.00		0.00	0.00	0.00	0.00
1 hour	0.00	0.00	0.00		0.00	0.00	0.00
3 hours	0.00	0.00	0.00	0.00		**0.54**	0.00
6 hours	0.00	0.00	0.00	0.00	**0.54**		**0.18**
20 hours	0.00	0.00	0.00	0.00	0.00	**0.18**	

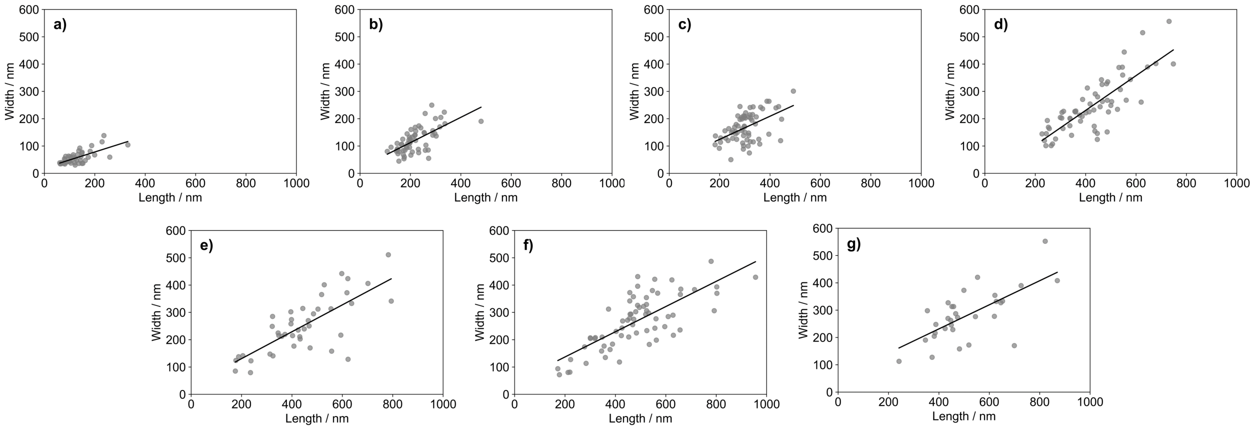

Figure S16. Aspect ratios of rod-shaped NP sizes (2nd row) for reactions lasting varying times (Figure 4): (a) one minute, (b) five minutes, (c) 15 minutes, (d) one hour, (e) three hours, (f) six hours and (g) 20 hours. The fitted lines are based on the coefficients of linear regression analysis. The slopes (95% confidence interval) for (a)-(g) are 0.29 (0.19 to 0.39), 0.46 (0.32 to 0.60), 0.42 (0.24 to 0.60), 0.64 (0.52 to 0.75), 0.49 (0.35 to 0.63), 0.46 (0.36 to 0.56) and 0.44 (0.26 to 0.62), respectively.

Table S8. Aspect ratios (length/width) of rod-shaped NP sizes (2nd row) for reactions lasting varying times (Figure 4, errors report standard deviations) and p-values from Games-Howell pairwise testing that aspect ratios are the same for each pair of reactions. Values are in bold where the two mean sizes are statistically the same.

	1 minute	5 minutes	15 minutes	1 hour	3 hours	6 hours	20 hours
	2.4 ± 0.8	2.0 ± 0.7	2.0 ± 0.7	1.8 ± 0.5	1.9 ± 0.7	1.9 ± 0.5	1.9 ± 0.6
1 minute		**0.12**	**0.13**	0.01	**0.07**	0.03	**0.13**
5 minutes	**0.12**		**1.00**	**0.92**	**1.00**	**1.00**	**1.00**
15 minutes	**0.13**	**1.00**		**0.91**	**1.00**	**1.00**	**1.00**
1 hour	0.01	**0.92**	**0.91**		**1.00**	**0.98**	**0.99**
3 hours	**0.07**	**1.00**	**1.00**	**1.00**		**1.00**	**1.00**
6 hours	0.03	**1.00**	**1.00**	**0.98**	**1.00**		**1.00**
20 hours	**0.13**	**1.00**	**1.00**	**0.99**	**1.00**	**1.00**	

Reaction Scale-Up

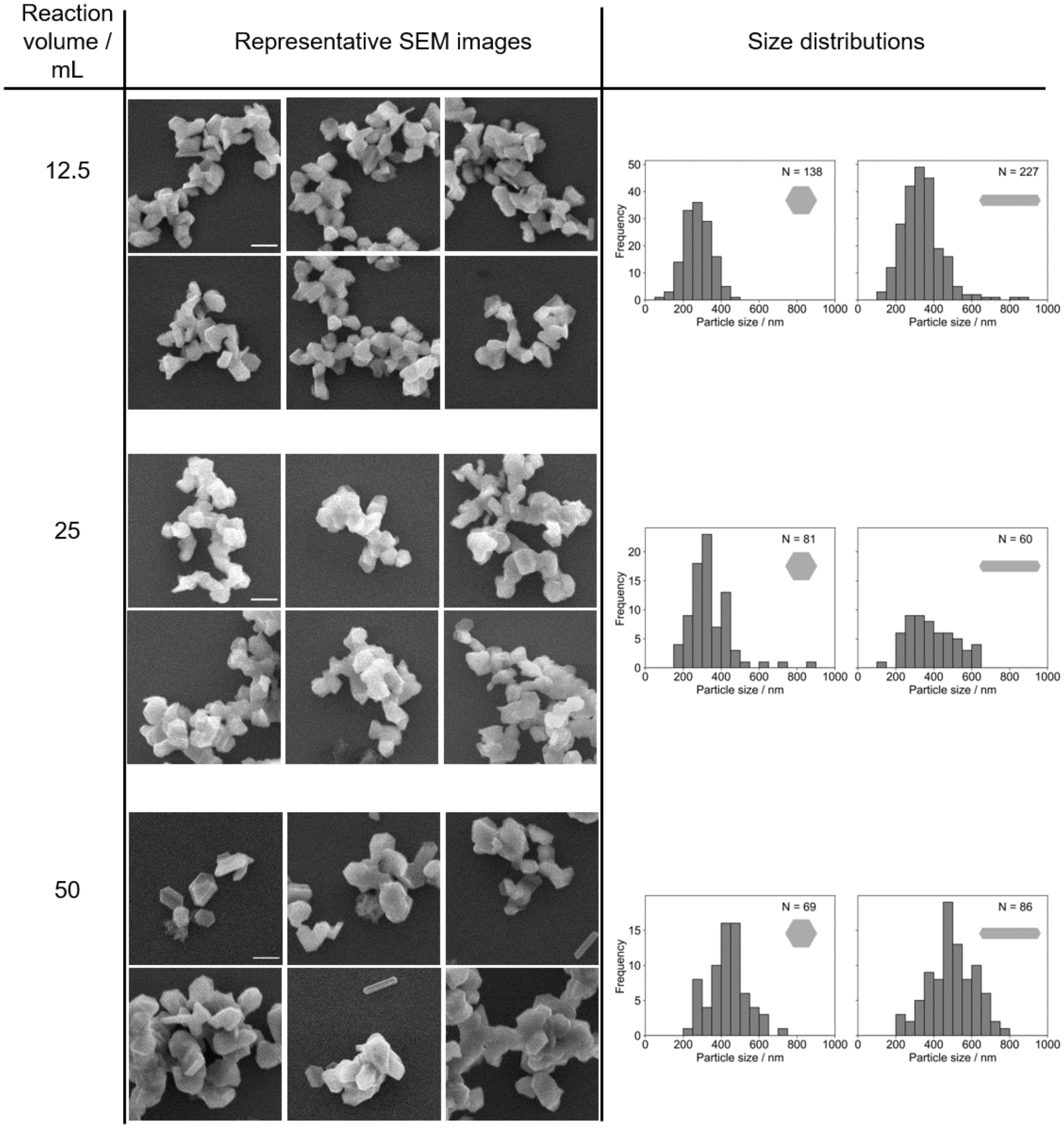

Figure S17. Representative SEM and size distributions of NPs from reaction volumes of 12.5 mL, 25 mL and 50 mL in 25 mL, 50 mL and 100 mL flasks respectively, with reactant quantities scaled proportionally. Scale bars, 500 nm.

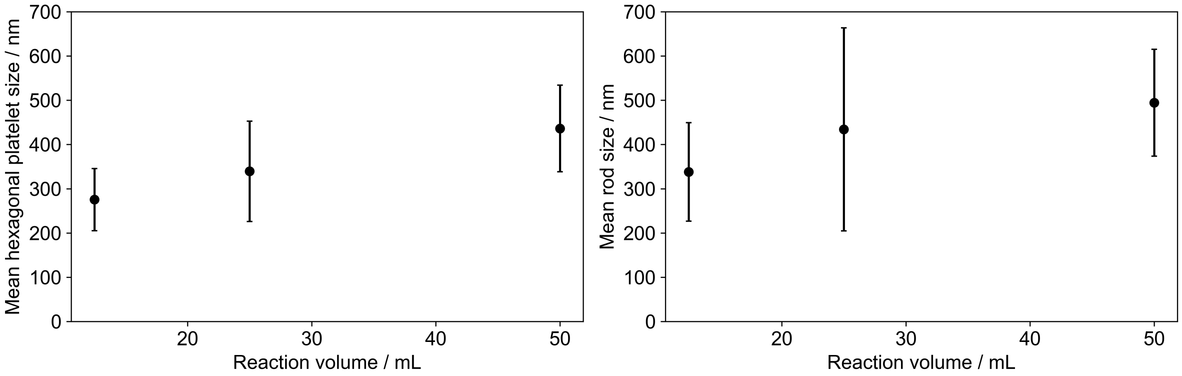

Figure S18. Comparison of hexagonal platelets and rod-shaped NP sizes for the reactions on different scales in Figure S17.

Table S9. Hexagonal platelet sizes (2nd row) for reactions on different scales with different reaction volumes (Figures S17, S18) and p-values from Games-Howell pairwise testing that sizes of hexagonal platelets are the same for each pair of reactions. No two mean sizes were statistically the same.

Reaction volume / mL	12.5	25	50
	280 ± 70 nm (25%)	340 ± 110 (33%)	440 ± 100 nm (22%)
12.5		0.00	0.00
25	0.00		0.00
50	0.00	0.00	

Table S10. Rod-shaped NP sizes (2nd row) for reactions on different scales with different reaction volumes (Figures S17, S18) and p-values from Games-Howell pairwise testing that sizes of rod-shaped NPs are the same for each pair of reactions. Values are in bold where the two mean sizes are statistically the same.

Reaction volume / mL	12.5	25	50
	340 ± 110 nm (33%)	400 ± 200 (53%)	490 ± 120 nm (24%)
12.5		0.01	0.00
25	0.01		**0.16**
50	0.00	**0.16**	

Overall Reaction Concentration

Table S11. Hexagonal platelet sizes (2nd row) for reactions at varying overall concentration (Figures 5 and S17) and p-values from Games-Howell pairwise testing that sizes of hexagonal platelets are the same for each pair of reactions. Reaction volumes, 12.5 mL or 50 mL†. Values are in bold where the two mean sizes are statistically the same.

$[MgBu_2]$ / M	0.014†	0.035	0.070	0.105	0.140†	0.140	0.280	0.350
	90 ± 40 nm (43%)	170 ± 40 nm (23%)	250 ± 40 nm (17%)	380 ± 70 nm (19%)	440 ± 100 nm (22%)	280 ± 70 nm (25%)	340 ± 140 nm (41%)	320 ± 110 nm (34%)
0.014†		0.00	0.00	0.00	0.00	0.00	0.00	0.00
0.035	0.00		0.00	0.00	0.00	0.00	0.00	0.00
0.070	0.00	0.00		0.00	0.00	0.01	0.00	0.00
0.105	0.00	0.00	0.00		0.00	0.00	**0.36**	0.00
0.140†	0.00	0.00	0.00	0.00		0.00	0.00	0.00
0.140	0.00	0.00	0.01	0.00	0.00		0.01	**0.05**
0.280	0.00	0.00	0.00	**0.36**	0.00	0.01		**0.98**
0.350	0.00	0.00	0.00	0.00	0.00	**0.05**	**0.98**	

Table S12. Rod-shaped NP sizes (2nd row) for reactions at varying overall concentration (Figures 5 and S17) and p-values from Games-Howell pairwise testing that sizes of rod-shaped NPs are the same for each pair of reactions. Reaction volumes, 12.5 mL or 50 mL†. Values are in bold where the two mean sizes are statistically the same.

$[MgBu_2]$ / M	0.014†	0.035	0.070	0.105	0.140†	0.140	0.280	0.350
	100 ± 40 nm (38%)	210 ± 50 nm (24%)	300 ± 80 nm (28%)	410 ± 120 nm (29%)	490 ±120 nm (24%)	340 ± 110 nm (33%)	400 ± 180 nm (45%)	350 ± 100 nm (28%)
0.014†		0.00	0.00	0.00	0.00	0.00	0.00	0.00
0.035	0.00		0.00	0.00	0.00	0.00	0.00	0.00
0.070	0.00	0.00		0.00	0.00	0.00	0.00	0.00
0.105	0.00	0.00	0.00		0.00	0.00	**1.00**	0.01
0.140†	0.00	0.00	0.00	0.00		0.00	0.01	0.00
0.140	0.00	0.00	0.00	0.00	0.00		**0.17**	**0.99**
0.280	0.00	0.00	0.00	**1.00**	0.01	**0.17**		**0.51**
0.350	0.00	0.00	0.00	0.01	0.00	**0.99**	**0.51**	

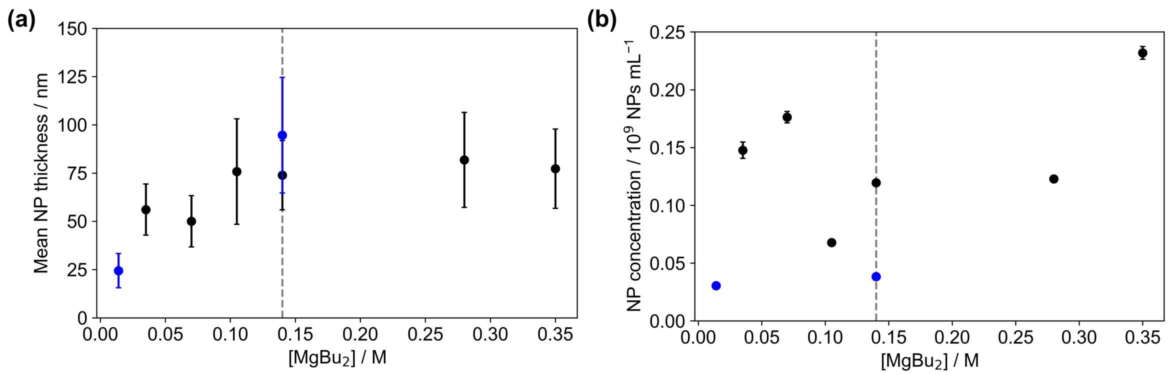

Figure S19. (a) Effect of overall reaction concentration, quantified by [$MgBu_2$], on NP thickness, with reaction volumes of 12.5 mL in black and 50 mL in blue, on NP thickness measured by SEM. (b) NP concentration with varying overall reaction concentration, calculated from the Mg content measured by ICP-MS and the average hexagonal platelet tip-to-tip length and thickness. The dashed lines mark the standard reaction concentration (0.14 M $MgBu_2$).

Temperature

Table S13. Hexagonal platelet sizes (2nd row) for reactions at varying overall concentration at 0 °C (Figure 6) and p-values from Games-Howell pairwise testing that sizes of hexagonal platelets are the same for each pair of reactions. Values are in bold where the two mean sizes are statistically the same.

[$MgBu_2$] / M	0.035	0.070	0.105	0.140
	250 ± 70 nm (30%)	300 ± 90 nm (31%)	700 ± 200 nm (29%)	1300 ± 500 nm (41%)
0.035		0.00	0.00	0.00
0.070	0.00		0.00	0.00
0.105	0.00	0.00		0.00
0.140	0.00	0.00	0.00	

Table S14. Rod-shaped NP sizes (2nd row) for reactions at varying overall concentration at 0 °C (Figure 6) and p-values from Games-Howell pairwise testing that sizes of rod-shaped NPs are the same for each pair of reactions. Values are in bold where the two mean sizes are statistically the same.

[$MgBu_2$] / M	0.035	0.070	0.105	0.140
	350 ± 100 nm (28%)	500 ± 200 nm (41%)	1000 ± 300 nm (31%)	1600 ± 500 nm (33%)
0.035		0.00	0.00	0.00
0.070	0.00		0.00	0.00
0.105	0.00	0.00		0.00
0.140	0.00	0.00	0.00	

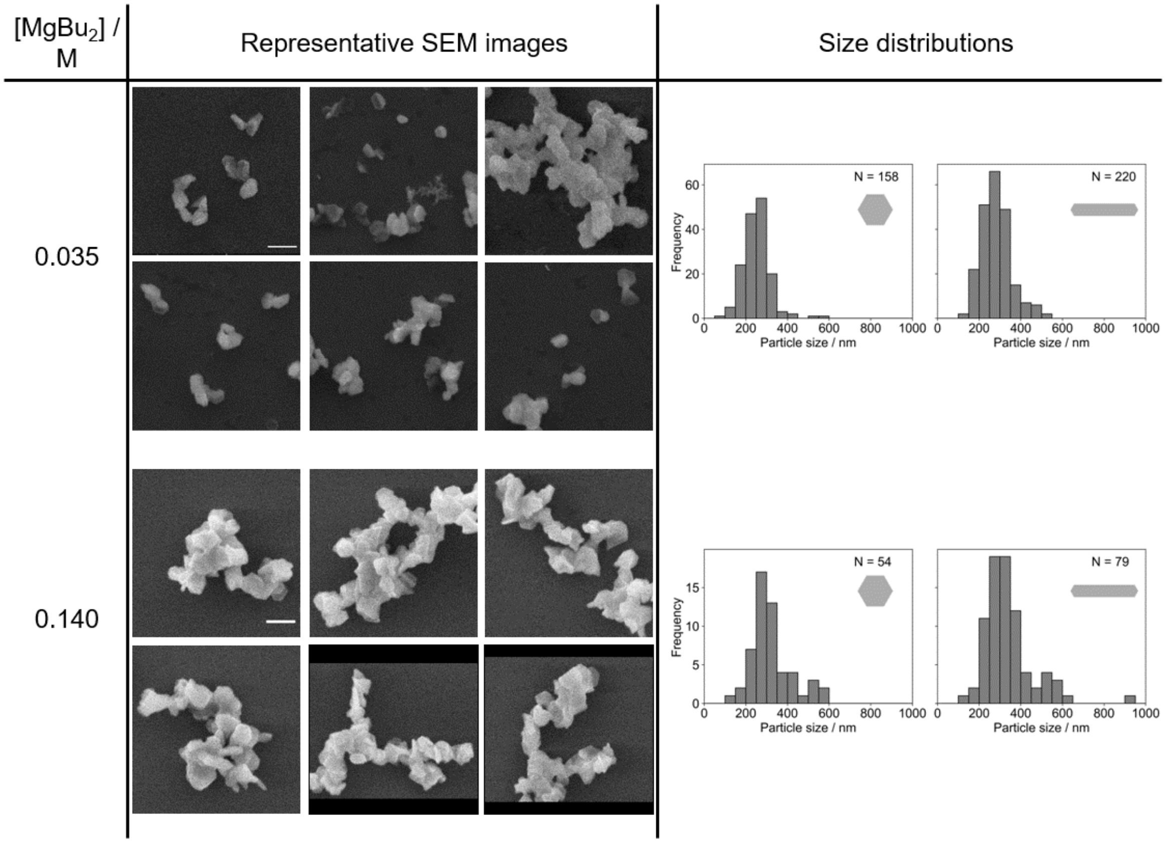

Figure S20. Representative SEM and size distributions of reactions at 40 °C with varying [$MgBu_2$]. Scale bars, 500 nm.

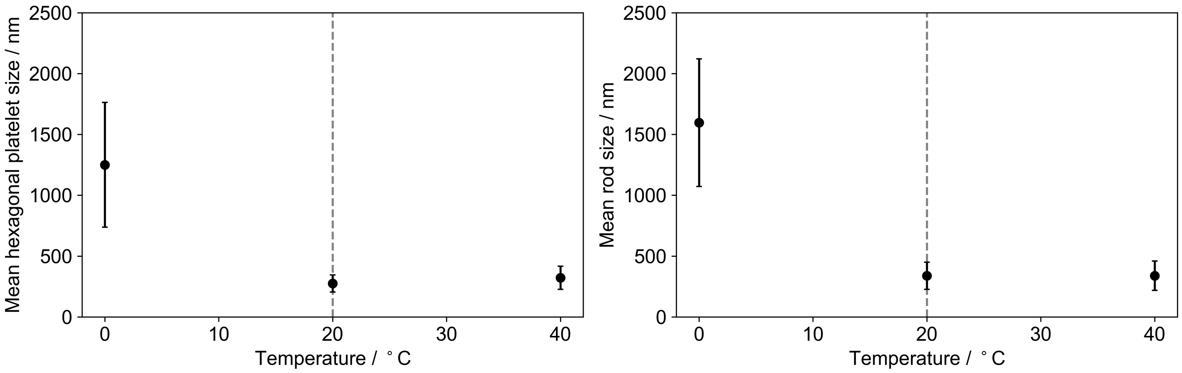

Figure S21. Comparison of hexagonal platelet and rod-shaped NP sizes for reactions at different temperatures at [$MgBu_2$] = 0.14 M as in Figures 4, S20. The dotted line marks the standard reaction temperature.

Table S15. Hexagonal platelet sizes (2nd row) for reactions at different temperatures at [$MgBu_2$] = 0.14 M (Figures 6, S20, S21) and p-values from Games-Howell pairwise testing that sizes of hexagonal platelets are the same for each pair of reactions. Values are in bold where the two mean sizes are statistically the same.

	0 ºC	20 ºC	40 ºC
	1300 ± 500 nm (41%)	280 ± 70 nm (25%)	320 ± 100 nm (30%)
0 ºC		0.00	0.00
20 ºC	0.00		0.00
40 ºC	0.00	0.00	

Table S16. Rod-shaped NP sizes (2nd row) for reactions at different temperatures at [$MgBu_2$] = 0.14 M (Figures 6, S20, S21) and p-values from Games-Howell pairwise testing that sizes of rod-shaped NPs are the same for each pair of reactions. Values are in bold where the two mean sizes are statistically the same.

	0 ºC	20 ºC	40 ºC
	1600 ± 500 nm (33%)	340 ± 110 nm (33%)	340 ± 120 nm (36%)
0 ºC		0.00	0.00
20 ºC	0.00		**1.00**
40 ºC	0.00	**1.00**	

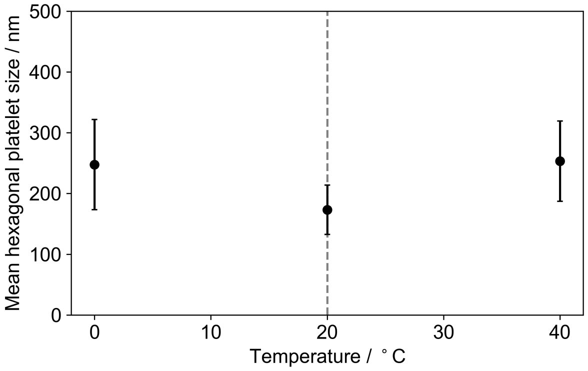

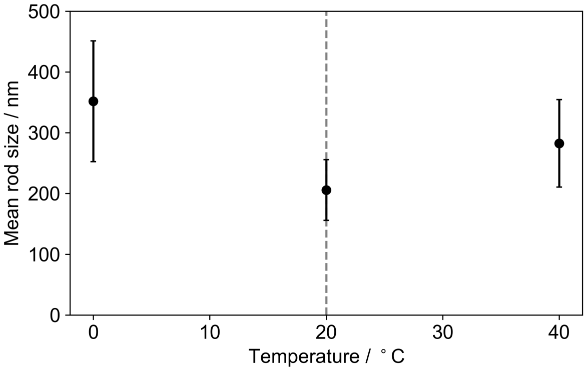

Figure S22. Comparison of hexagonal platelet and rod-shaped NP sizes for reactions at different temperatures at [$MgBu_2$] = 0.035 M as in Figures 5, S20. The dotted line marks the standard reaction.

Table S17. Hexagonal platelet sizes (2nd row) for reactions at different temperatures at [$MgBu_2$] = 0.035 M (Figures 5, S20, S22) and p-values from Games-Howell pairwise testing that sizes of hexagonal platelets are the same for each pair of reactions. Values are in bold where the two mean sizes are statistically the same.

	0 ºC	20 ºC	40 ºC
	250 ± 70 nm (30%)	170 ± 40 nm (23%)	250 ± 70 nm (26%)
0 ºC		0.00	**0.76**
20 ºC	0.00		0.00
40 ºC	**0.76**	0.00	

Table S18. Rod-shaped NP sizes (2nd row) for reactions at different temperatures at [$MgBu_2$] = 0.035 M (Figures 5, S20, S22) and p-values from Games-Howell pairwise testing that sizes of rod-shaped NPs are the same for each pair of reactions. Values are in bold where the two mean sizes are statistically the same.

	0 ºC	20 ºC	40 ºC
	350 ± 100 nm (28%)	210 ± 50 nm (24%)	280 ± 70 nm (25%)
0 ºC		0.00	0.00
20 ºC	0.00		0.00
40 ºC	0.00	0.00	

Electron Carrier

Table S19. Hexagonal platelet sizes (2nd row) for reactions with different electron carriers (Figure 7) and p-values from Games-Howell pairwise testing that sizes of hexagonal platelets are the same for each pair of reactions. Values are in bold where the two mean sizes are statistically the same.

	Biphenyl	Naphthalene	Phenanthrene
	110 ± 40 nm (39%)	280 ± 70 nm (25%)	260 ± 110 nm (43%)
Biphenyl		0.00	0.00
Naphthalene	0.00		**0.52**
Phenanthrene	0.00	**0.52**	

Table S20. Rod-shaped NP sizes (2nd row) for reactions with different electron carriers (Figure 7) and p-values from Games-Howell pairwise testing that sizes of rod-shaped NPs are the same for each pair of reactions. Values are in bold where the two mean sizes are statistically the same.

	Biphenyl	Naphthalene	Phenanthrene
	140 ± 50 nm (34%)	340 ± 110 nm (33%)	400 ±200 nm (51%)
Biphenyl		0.00	0.00
Naphthalene	0.00		0.01
Phenanthrene	0.00	0.01	

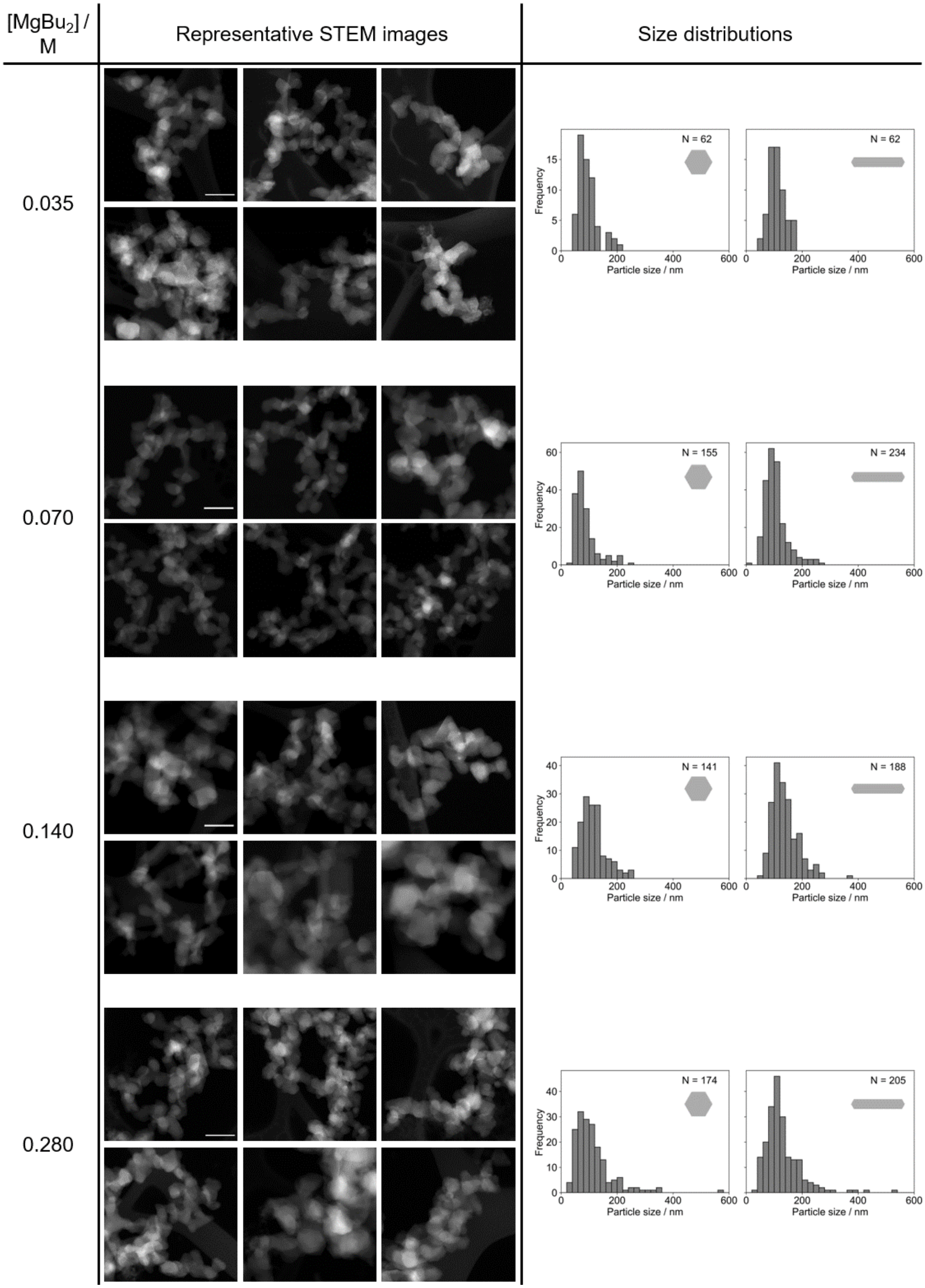

Figure S23. Representative HAADF-STEM and size distributions of reactions with biphenyl electron carrier with varying overall concentration. Scale bars, 200 nm.

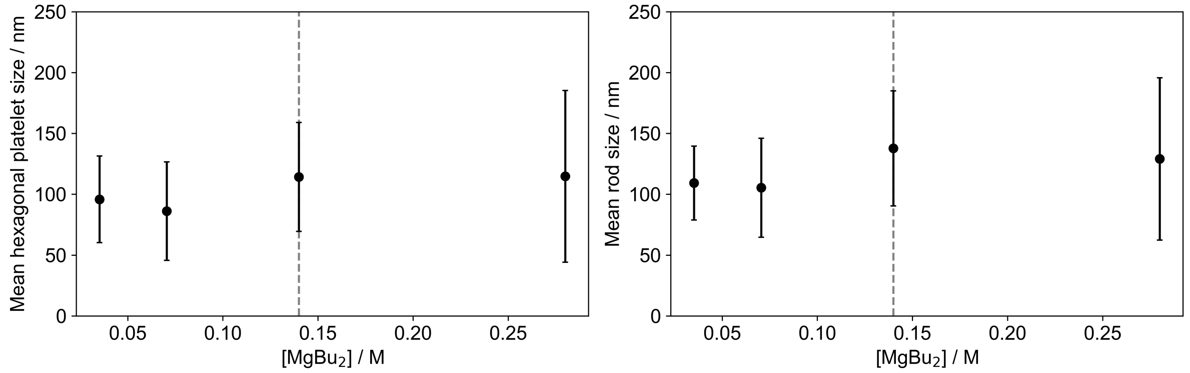

Figure S24. Comparison of hexagonal platelet and rod-shaped NP sizes for the reactions with biphenyl electron carrier with varying overall concentration as in Figure S23. The dotted line marks the standard reaction concentration.

Table S21. Hexagonal platelet sizes (2nd row) for reactions with biphenyl electron carrier with varying overall concentration (Figures S23, S24) and p-values from Games-Howell pairwise testing that sizes of hexagonal platelets are the same for each pair of reactions. Values are in bold where the two mean sizes are statistically the same.

$[MgBu_2]$/ M	0.035	0.070	0.140	0.280
	100 ± 40 nm (37%)	90 ± 40 nm (47%)	110 ± 40 (39%)	110 ± 70 nm (62%)
0.035		**0.31**	0.01	0.04
0.070	**0.31**		0.00	0.00
0.140	0.01	0.00		**1.00**
0.280	0.04	0.00	**1.00**	

Table S22. Rod-shaped NP sizes (2nd row) for reactions with biphenyl electron carrier with varying overall concentration (Figures S23, S24) and p-values from Games-Howell pairwise testing that sizes of rod-shaped NPs are the same for each pair of reactions. Values are in bold where the two mean sizes are statistically the same.

$[MgBu_2]$/ M	0.035	0.070	0.140	0.280
	110 ± 30 nm (28%)	110 ± 40 nm (39%)	140 ± 50 nm (34%)	130 ± 70 nm (52%)
0.035		**0.84**	0.00	0.01
0.070	**0.84**		0.00	0.00
0.140	0.00	0.00		**0.44**
0.280	0.01	0.00	**0.44**	

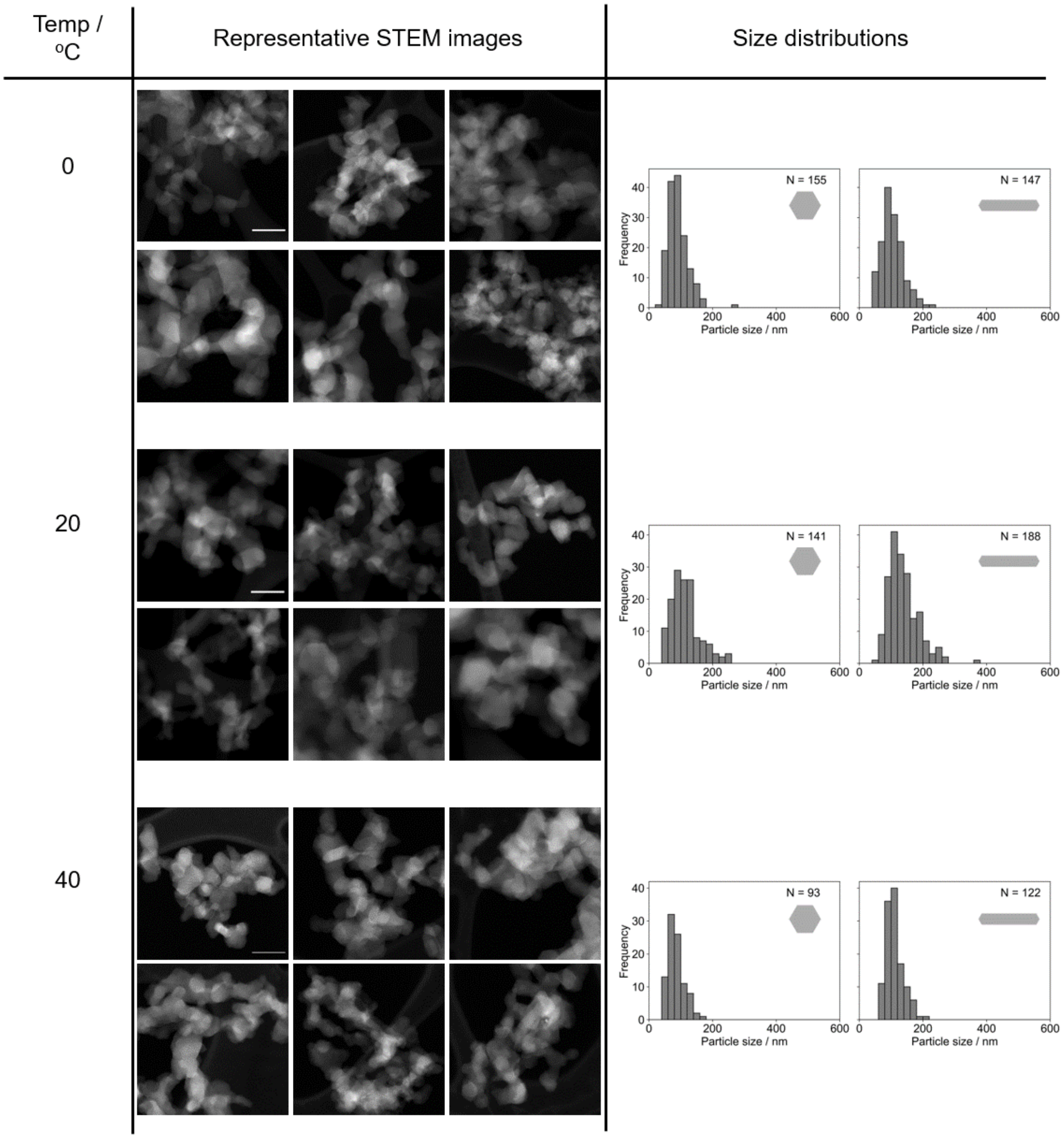

Figure S25. Representative HAADF-STEM and size distributions of reactions with biphenyl electron carrier at varying temperature. Scale bars, 200 nm.

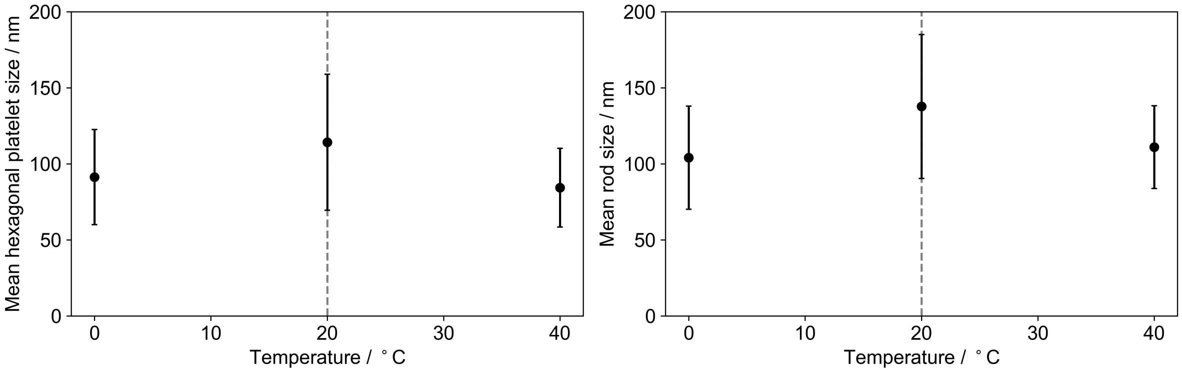

Figure S26. Comparison of hexagonal platelet and rod-shaped NP sizes for the reactions with biphenyl electron carrier at varying temperature as in Figure S25. The dotted line marks the standard reaction temperature.

Table S23. Hexagonal platelet sizes (2nd row) for reactions with biphenyl electron carrier at different temperatures (Figures S25, S26) and p-values from Games-Howell pairwise testing that sizes of hexagonal platelets are the same for each pair of reactions. Values are in bold where the two mean sizes are statistically the same.

	0 ºC	20 ºC	40 ºC
	90 ± 30 nm (34%)	110 ± 40 nm (39%)	80 ± 30 nm (31%)
0 ºC		0.00	**0.15**
20 ºC	0.00		0.00
40 ºC	**0.15**	0.00	

Table S24. Rod-shaped NP sizes (2nd row) for reactions with biphenyl electron carrier at different temperatures (Figures S25, S26) and p-values from Games-Howell pairwise testing that sizes of rod-shaped NPs are the same for each pair of reactions. Values are in bold where the two mean sizes are statistically the same.

	0 ºC	20 ºC	40 ºC
	100 ± 30 nm (33%)	140 ± 50 nm (34%)	110 ± 30 nm (24%)
0 ºC		0.00	**0.15**
20 ºC	0.00		0.00
40 ºC	**0.15**	0.00	

Additives

Table S25. Hexagonal platelet sizes (2nd row) for reactions with different additives (Figure 8) and p-values from Games-Howell pairwise testing that sizes of hexagonal platelets are the same for each pair of reactions. Values are in bold where the two mean sizes are statistically the same.

	Fe^{III}	Ni^{II}	Fe^{II}	V^{II}	Al^{III}
	100 ± 40 nm (43%)	110 ± 30 nm (24%)	170 ± 90 nm (52%)	340 ± 120 nm (34%)	370 ± 160 nm (42%)
Fe^{III}		**0.18**	0.00	0.00	0.00
Ni^{II}	**0.18**		0.00	0.00	0.00
Fe^{II}	0.00	0.00		0.00	0.00
V^{II}	0.00	0.00	0.00		**0.50**
Al^{III}	0.00	0.00	0.00	**0.50**	

Table S26. Rod-shaped NP sizes (2nd row) for reactions with different additives (Figure 8) and p-values from Games-Howell pairwise testing that sizes of rod-shaped NPs are the same for each pair of reactions. Values are in bold where the two mean sizes are statistically the same.

	Fe^{III}	Ni^{II}	Fe^{II}	V^{II}	Al^{III}
	120 ± 50 nm (37%)	160 ± 50 nm (33%)	200 ± 100 nm (50%)	380 ± 150 nm (39%)	390 ± 160 nm (42%)
Fe^{III}		0.00	0.00	0.00	0.00
Ni^{II}	0.00		0.00	0.00	0.00
Fe^{II}	0.00	0.00		0.00	0.00
V^{II}	0.00	0.00	0.00		**0.98**
Al^{III}	0.00	0.00	0.00	**0.98**	

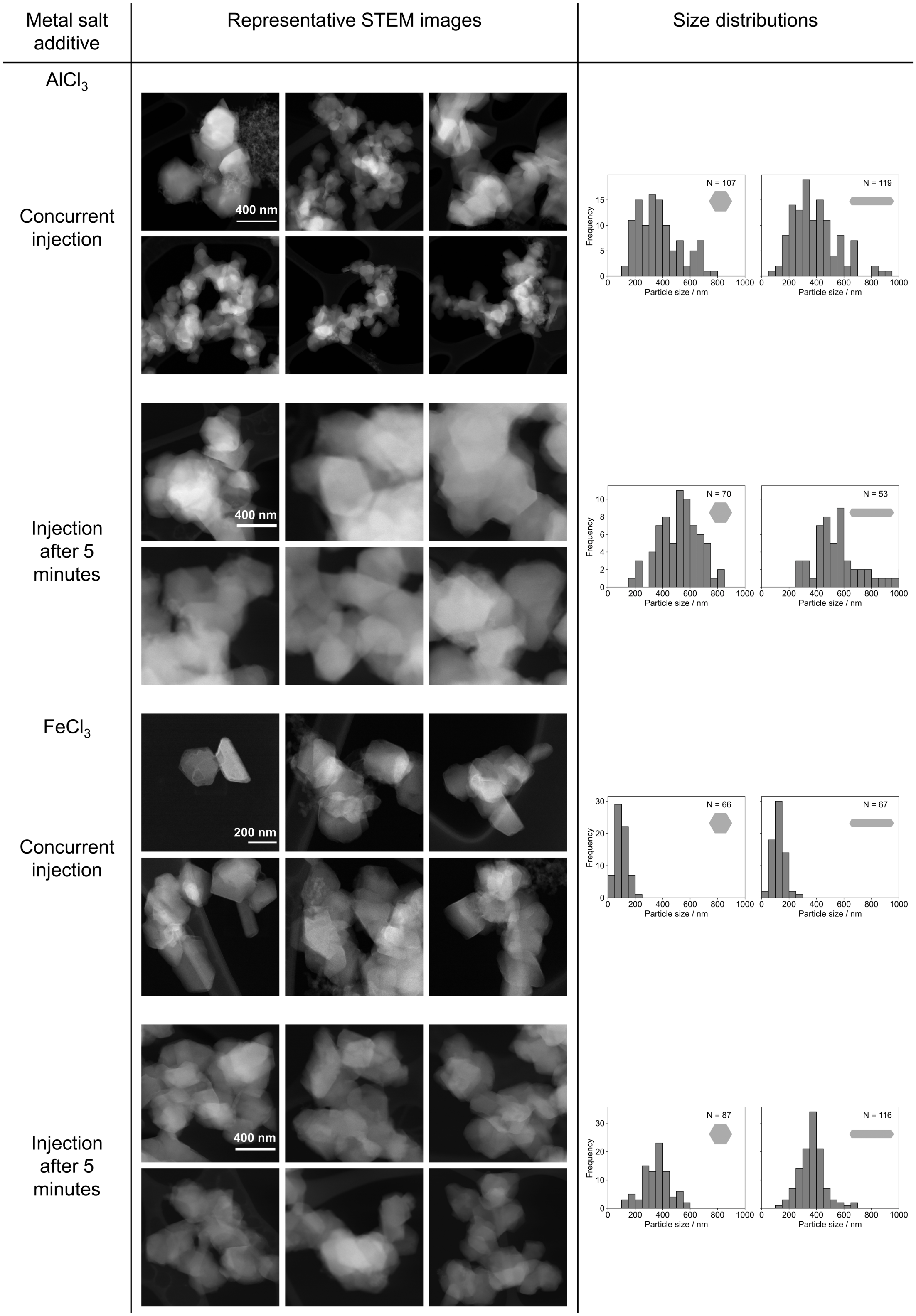

Figure S27. Representative HAADF-STEM and size distributions of reactions with $FeCl_3$ or $AlCl_3$ additives injected simultaneously with $MgBu_2$ or five minutes after.

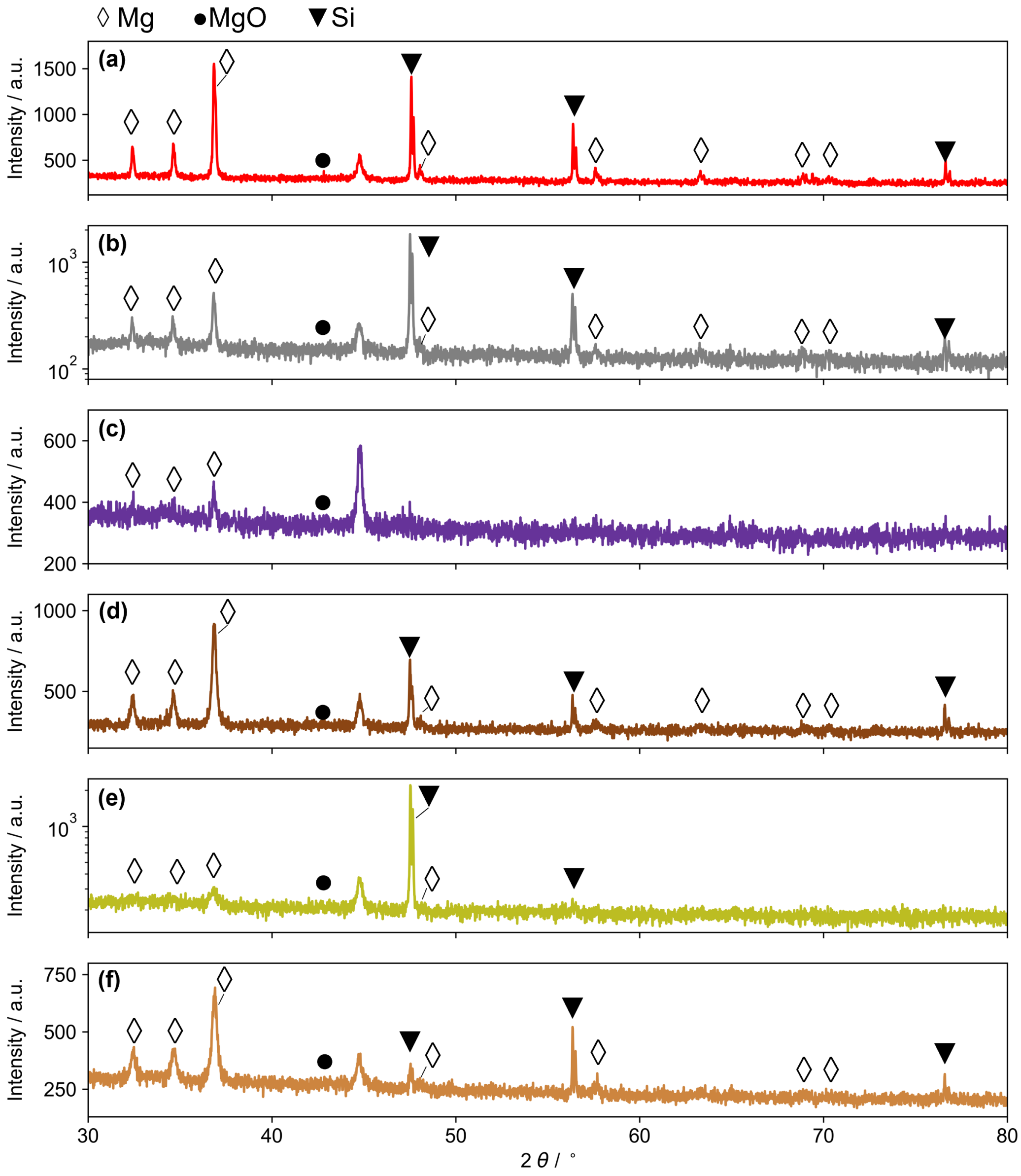

Figure S28. Composition of NPs from reactions with additives of varying reduction potentials. XRD patterns of the products of syntheses containing (a) no additives, (b) $AlCl_3$, (c) VCl_2, (d) $FeCl_2$, (e) $NiCl_2$ and (f) $FeCl_3$.

Table S27. Mg NP crystallite size from reactions with additives of varying reduction potentials, calculated via Rietveld refinement of XRD patterns in Figure S28. The quality of the fit is reported by the weighted profile *R*-factor (R_{wp}) and $\chi^2 = (R_{wp}/R_{exp})^2$ where R_{exp} is the expected R-factor (best expected fit).

Metal salt additive	E_{red} / V	Crystallite size / nm	Estimated standard deviation / nm	R_{wp}	χ^2
No additive	-	330	50	11.67	2.08
$AlCl_3$	–1.676	280	60	11.95	1.58
VCl_2	–1.13	250	130	6.07	1.08
$FeCl_2$	–0.44	60	2.3	6.85	1.16
$NiCl_2$	–0.257	30	5.0	10.2	1.54
$FeCl_3$	0.771	40	1.7	6.59	1.08

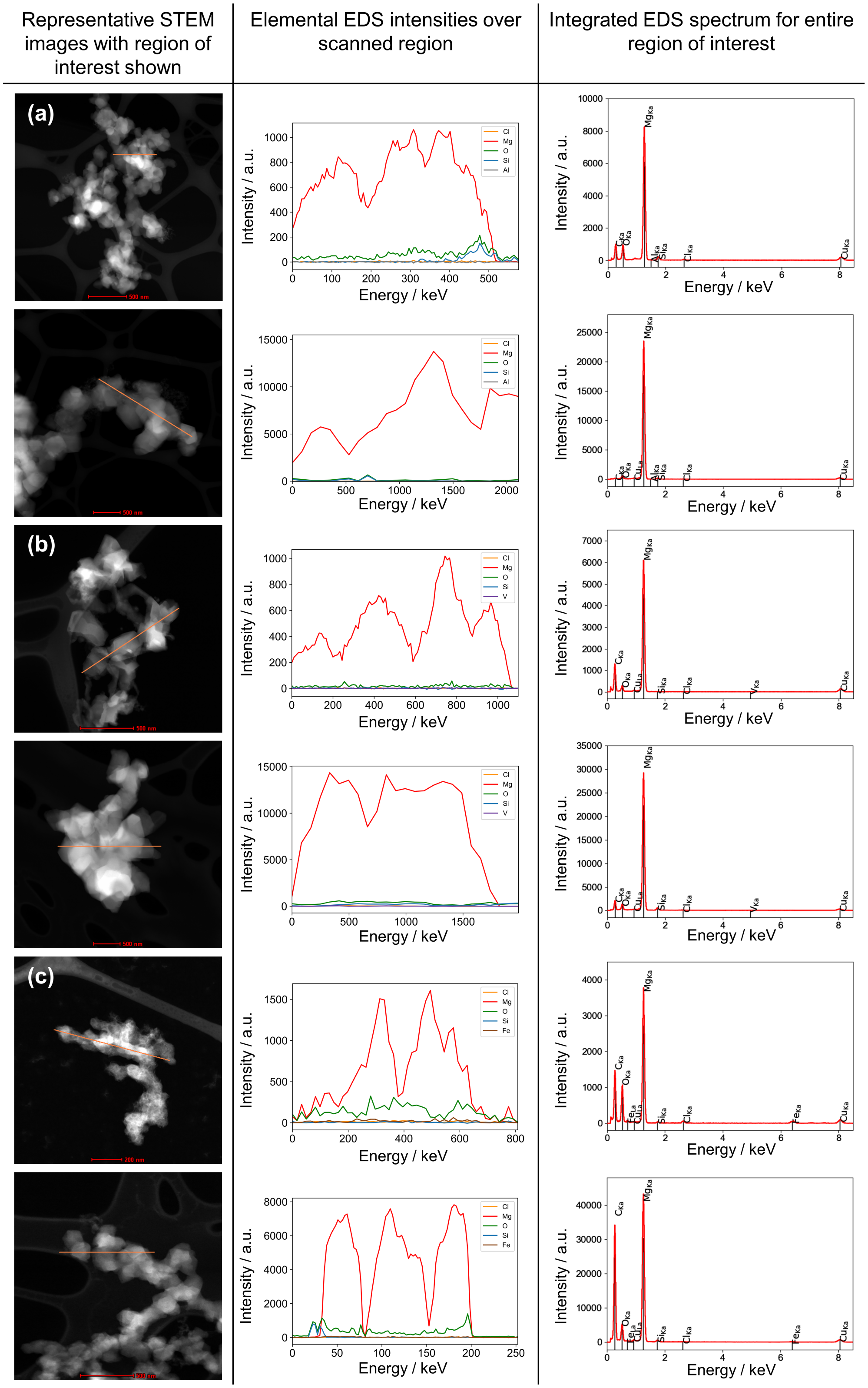

Figure S29. Composition of NPs from reactions with additives of varying reduction potentials: (a) $AlCl_3$, (b) VCl_2 and (c) $FeCl_2$. Integrated STEM-EDS spectra and elemental intensities (calculated from K_α lines) along the lines indicated, analysed and plotted using Hyperspy.[6]

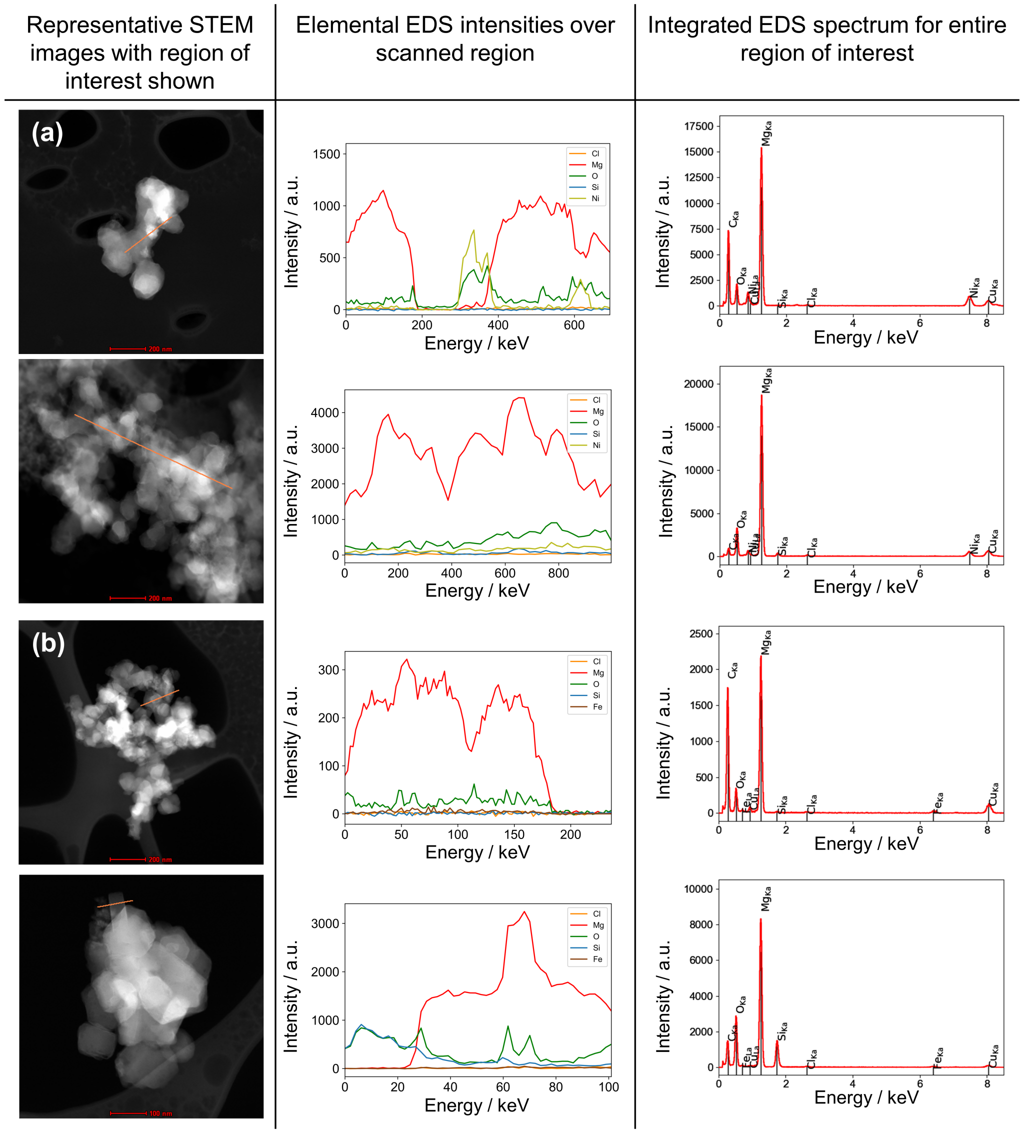

Figure S30. Composition of NPs from reactions with additives of varying reduction potentials: (a) $NiCl_2$ and (b) $FeCl_3$. Integrated STEM-EDS spectra and elemental intensities (calculated from K_α lines) along the lines indicated, analysed and plotted using Hyperspy.[6]

Metal Precursor: Reducing Agent Ratio. Amount of $MgBu_2$

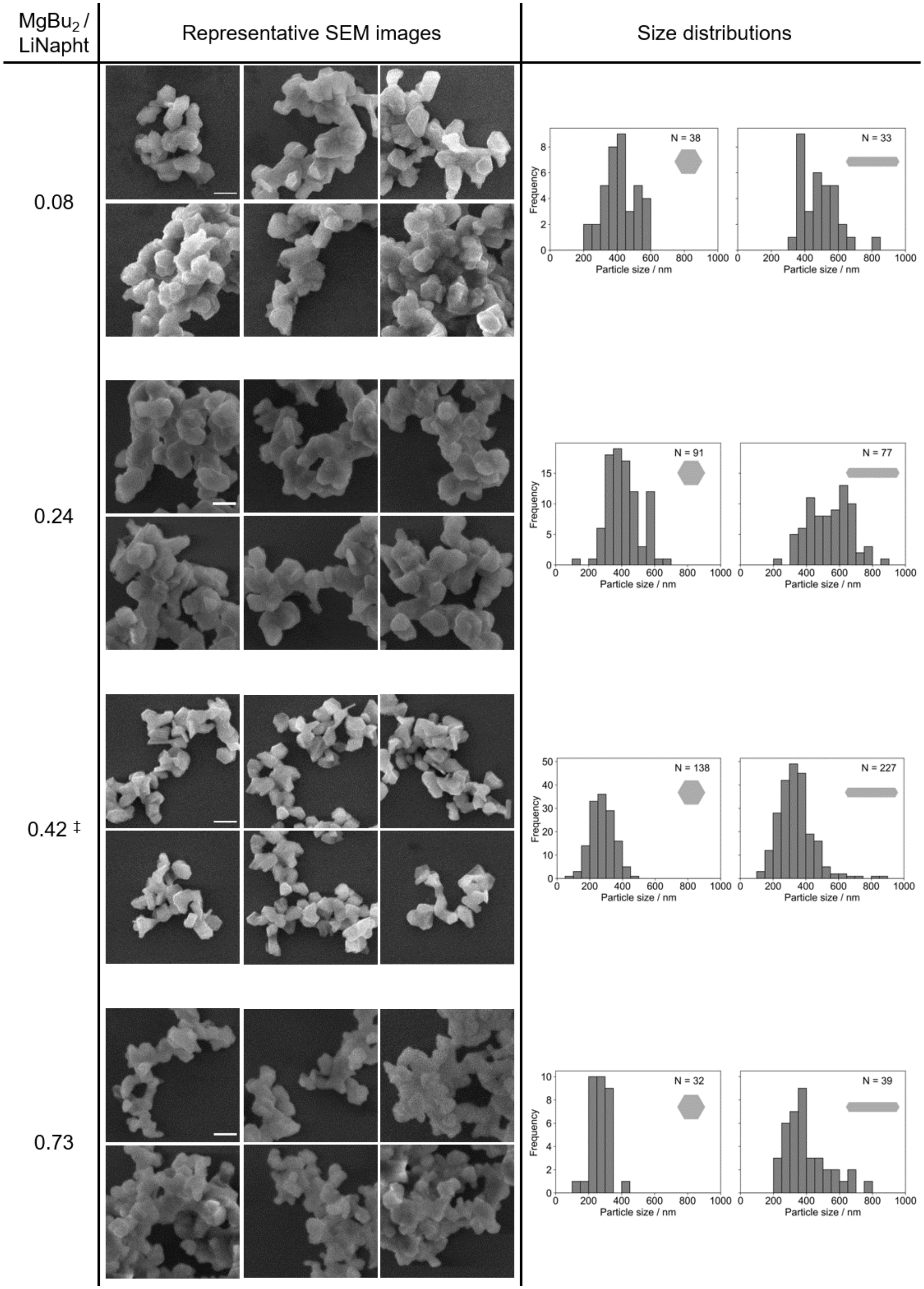

Figure S31. Representative SEM and size distributions of reactions with varying amounts of $MgBu_2$ at [LiNapht] = 0.32 M (synthesis using 0.028 g Li, 0.530 g naphthalene in 12.5 mL THF). Scale bars, 500 nm.

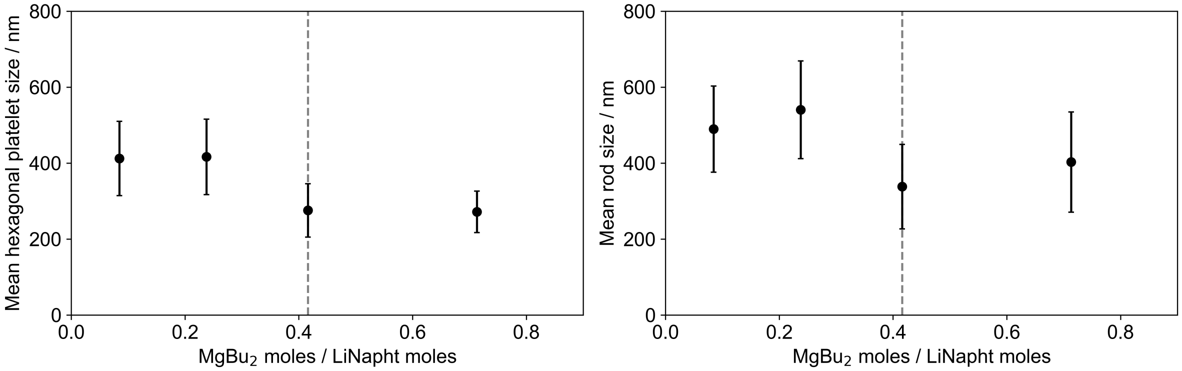

Figure S32. Comparison of hexagonal platelet and rod-shaped NP sizes for the reactions with varying amounts of $MgBu_2$ at [LiNapht] = 0.32 M as in Figure S31. The dotted line marks the standard ratio of $MgBu_2$ to LiNapht.

Table S28. Hexagonal platelet sizes (2nd row) for reactions with varying amounts of $MgBu_2$ at [LiNapht] = 0.32 M (Figures S31, S32) and p-values from Games-Howell pairwise testing that sizes of hexagonal platelets are the same for each pair of reactions. Values are in bold where the two mean sizes are statistically the same.

$MgBu_2$ / LiNapht	0.08	0.24	0.42	0.71
	410 ± 100 nm (24%)	420 ± 100 nm (24%)	280 ± 70 nm (25%)	270 ± 50 nm (20%)
0.08		**1.00**	0.00	0.00
0.24	**1.00**			0.00
0.42	0.00	0.00		**0.99**
0.71	0.00	0.00	**0.99**	

Table S29. Rod-shaped NP sizes (2nd row) for reactions with varying amounts of $MgBu_2$ at [LiNapht] = 0.32 M (Figures S31, S32) and p-values from Games-Howell pairwise testing that sizes of rod-shaped NPs are the same for each pair of reactions. Values are in bold where the two mean sizes are statistically the same.

$MgBu_2$ / LiNapht	0.08	0.24	0.42	0.71
	490 ± 110 nm (23%)	540 ± 130 nm (24%)	340 ± 110 nm (33%)	400 ± 130 nm (33%)
0.08		**0.17**	0.00	0.02
0.24	**0.17**			0.00
0.42	0.00	0.00		0.03
0.71	0.02	0.00	0.03	

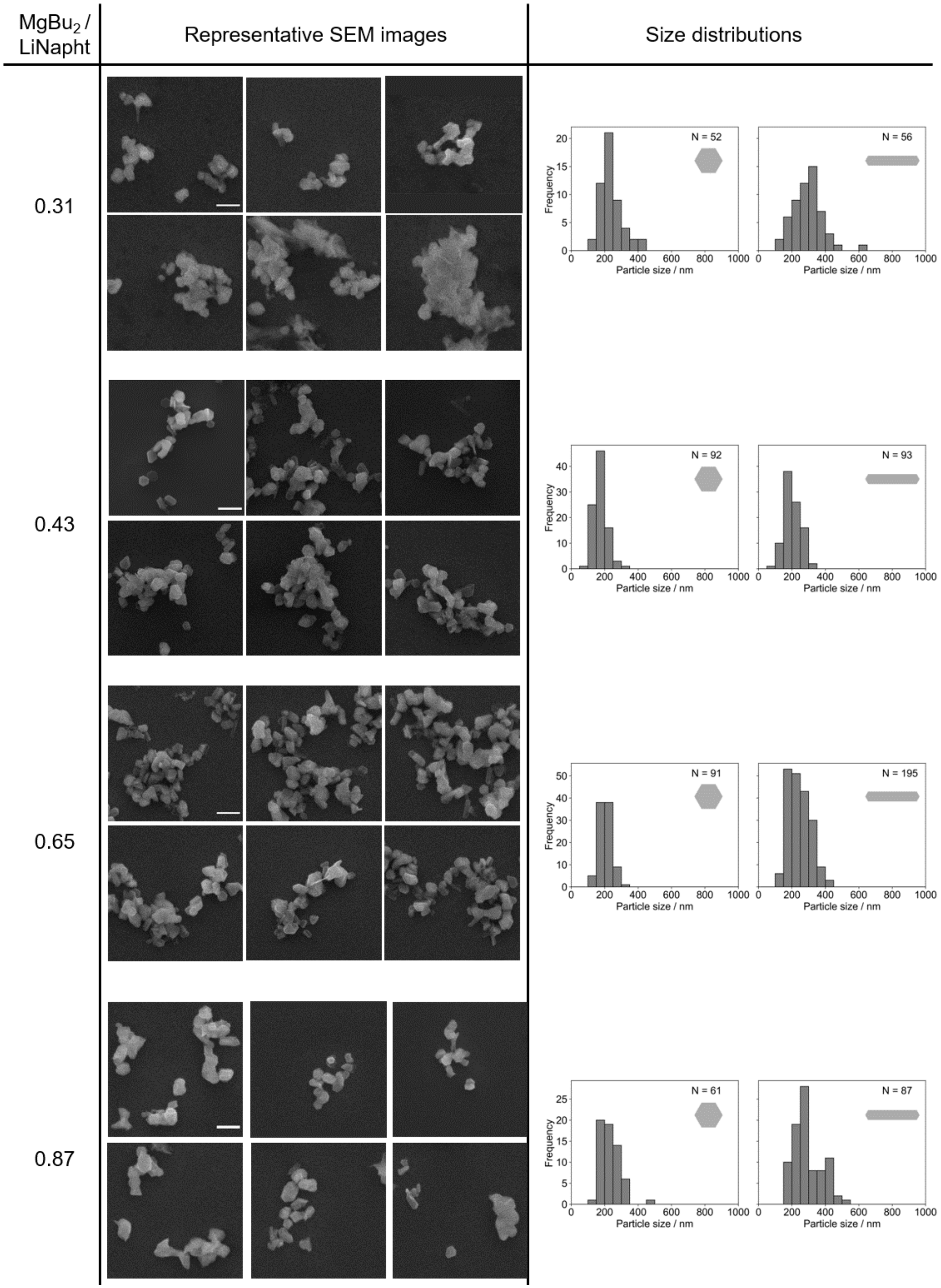

Figure S33. Representative SEM and size distributions of reactions with varying amounts of $MgBu_2$ at [LiNapht] = 0.08 M (synthesis using 0.007 g Li, 0.130 g naphthalene in 12.5 mL THF). Scale bar, 500 nm.

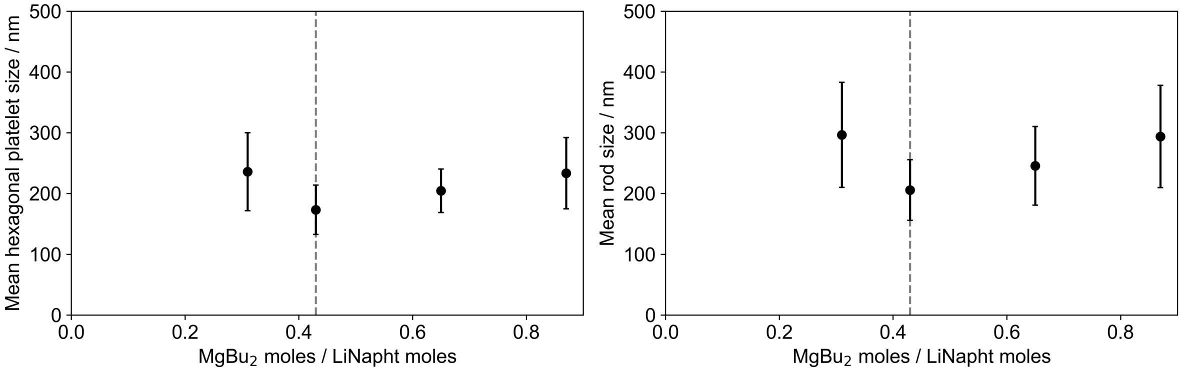

Figure S34. Comparison of hexagonal platelet and rod-shaped NP sizes for the reactions with varying amounts of $MgBu_2$ at [LiNapht] = 0.08 M as in Figure S33. The dotted line marks the standard ratio of $MgBu_2$ to LiNapht.

Table S30. Hexagonal platelet sizes (2nd row) for reactions with varying amounts of $MgBu_2$ at [LiNapht] = 0.08 M (Figures S33, S34) and p-values from Games-Howell pairwise testing that sizes of hexagonal platelets are the same for each pair of reactions. Values are in bold where the two mean sizes are statistically the same.

$MgBu_2$ / LiNapht	0.31	0.43	0.65	0.87
	240 ± 60 nm (27%)	170 ± 40 nm (23%)	210 ± 40 nm (18%)	230 ± 60 nm (25%)
0.31		0.00	0.01	**1.00**
0.43	0.00		0.00	0.00
0.65	0.01	0.00		0.00
0.87	**1.00**	0.00	0.00	

Table S31. Rod-shaped NP sizes (2nd row) for reactions with varying amounts of $MgBu_2$ at [LiNapht] = 0.08 M (Figures S33, S34) and p-values from Games-Howell pairwise testing that sizes of rod-shaped NPs are the same for each pair of reactions. Values are in bold where the two mean sizes are statistically the same.

$MgBu_2$ / LiNapht	0.31	0.43	0.65	0.87
	300 ± 90 nm (29%)	210 ± 50 nm (24%)	250 ± 60 nm (26%)	290 ±80 nm (29%)
0.31		0.00	0.00	**1.00**
0.43	0.00		0.00	0.00
0.65	0.00	0.00		0.00
0.87	**1.00**	0.00	0.00	

Metal Precursor:Reducing Agent Ratio. Amount of Naphthalene

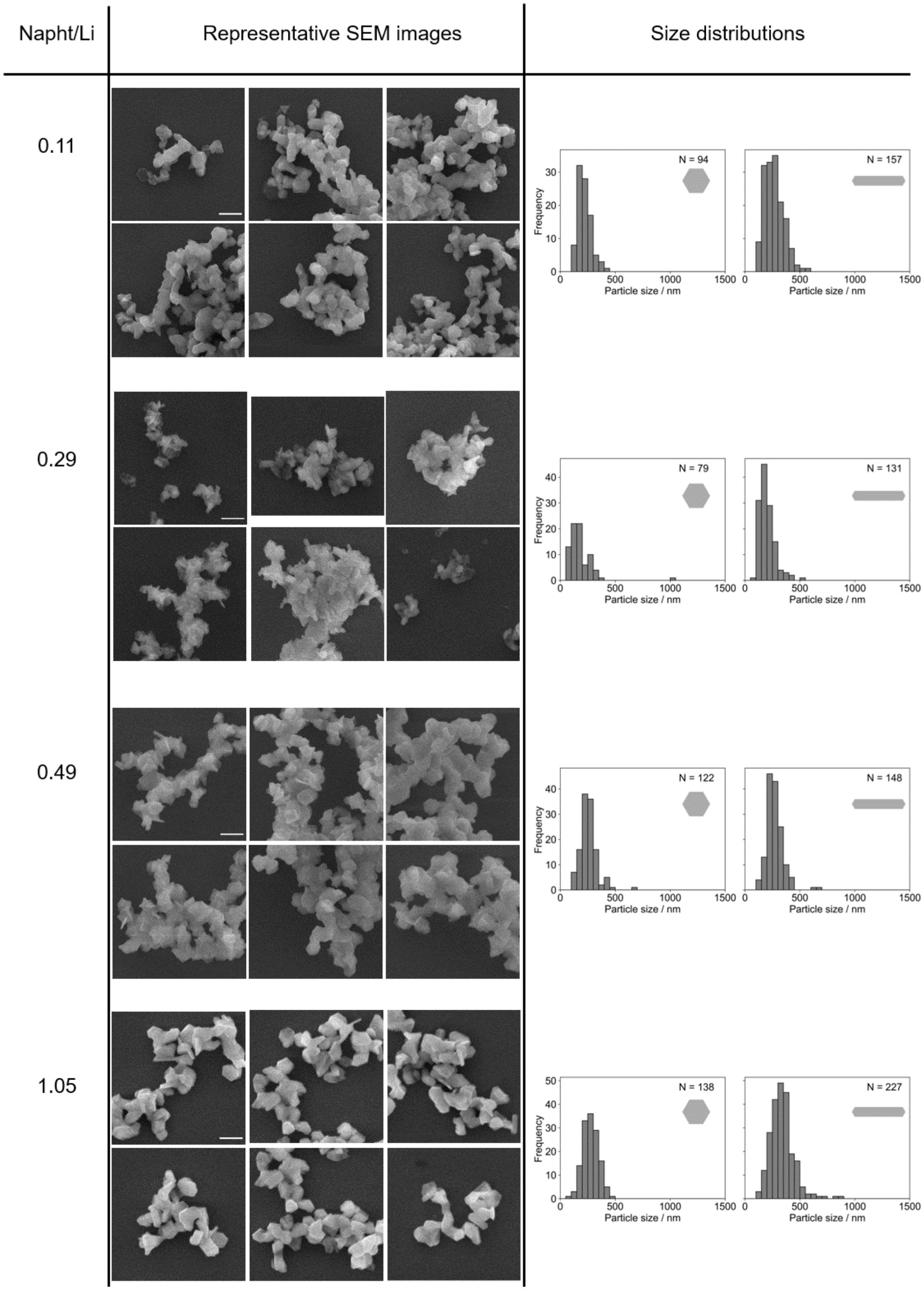

Figure S35. Representative SEM and size distributions of reactions with varying amounts of naphthalene at [$MgBu_2$] = 0.14 M (synthesis using 0.028 g Li, 1.75 mL $MgBu_2$ in 12.5 mL THF). Scale bars, 500 nm.

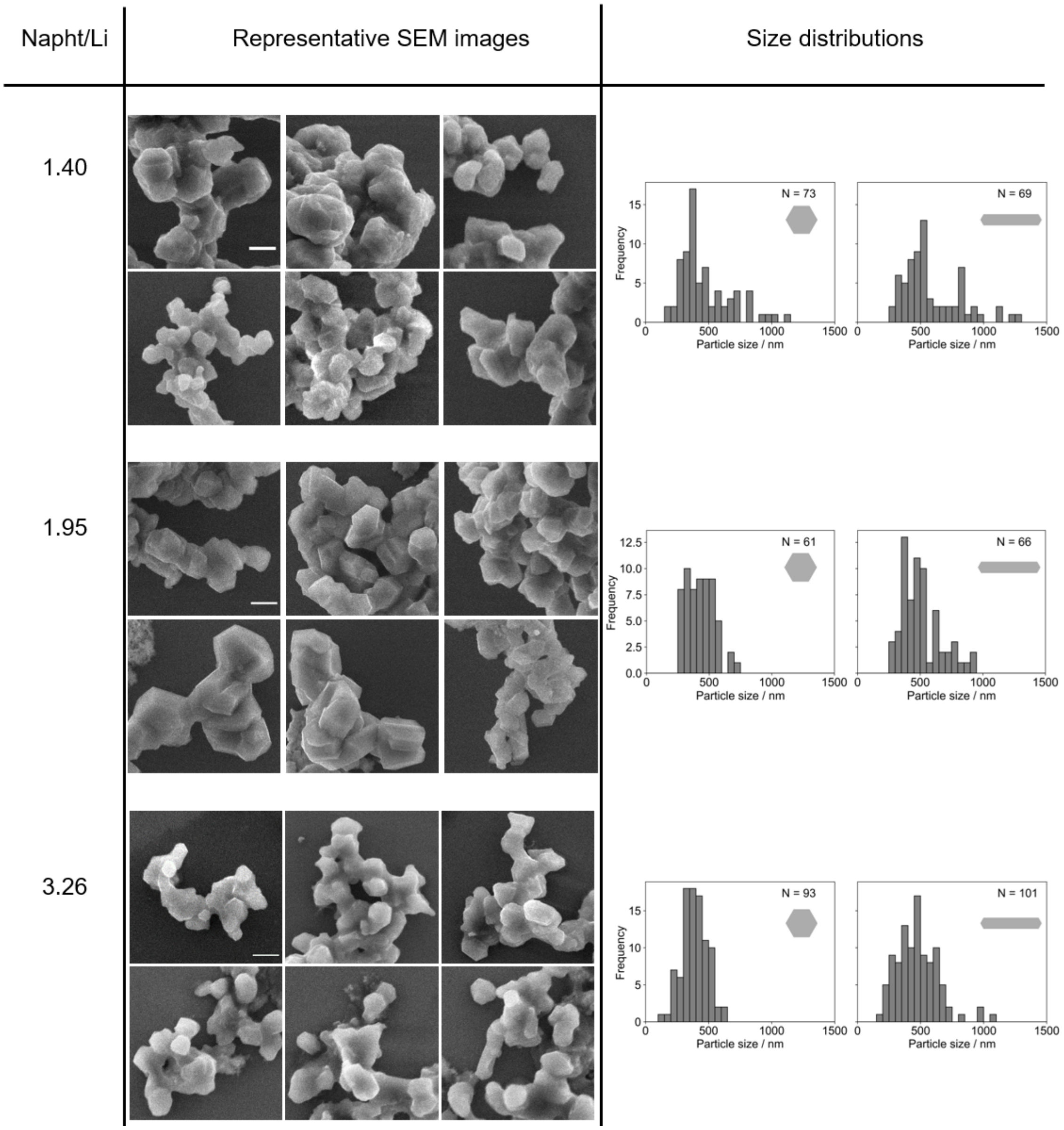

Figure S36. Representative SEM and size distributions of reactions with varying amounts of naphthalene at [$MgBu_2$] = 0.14 M (synthesis using 0.028 g Li, 1.75 mL $MgBu_2$ in 12.5 mL THF). Scale bars, 500 nm.

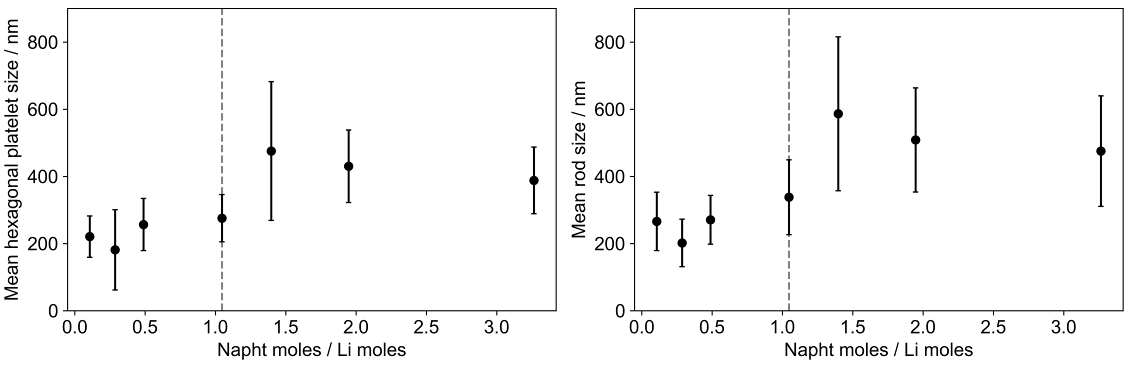

Figure S37. Comparison of hexagon platelet and rod-shaped NP sizes for the reactions with varying amounts of naphthalene at [$MgBu_2$] = 0.14 M as in Figures S35 and S36. The dotted line marks the standard ratio of naphthalene to Li.

Table S32. Hexagonal platelet sizes (2nd row) for reactions with varying amounts of naphthalene at [$MgBu_2$] = 0.14 M (Figures S35–S37) and p-values from Games-Howell pairwise testing that sizes of hexagonal platelets are the same for each pair of reactions. Values are in bold where the two mean sizes are statistically the same.

Napht/Li	0.11	0.29	0.49	1.05	1.40	1.95	3.26
	220 ± 60 nm (28%)	180 ± 120 nm (66%)	260 ± 80 nm (30%)	180 ± 70 nm (25%)	480 ± 200 nm (43%)	430 ± 110 nm (25%)	390 ± 100 nm (26%)
0.11		**0.12**	0.00	0.00	0.00	0.00	0.00
0.29	**0.12**		0.00	**0.12**	0.00	0.00	0.00
0.49	0.00	0.00		0.00	0.00	0.00	0.00
1.05	0.00	**0.12**	0.00		0.00	0.00	0.00
1.40	0.00	0.00	0.00	0.00		**0.67**	0.02
1.95	0.00	0.00	0.00	0.00	**0.67**		**0.19**
3.26	0.00	0.00	0.00	0.00	0.02	**0.19**	

Table S33. Rod-shaped NP sizes (2nd row) for reactions with varying amounts of naphthalene at [$MgBu_2$] = 0.14 M (Figures S35–S37) and p-values from Games-Howell pairwise testing that sizes of rod-shaped NPs are the same for each pair of reactions. Values are in bold where the two mean sizes are statistically the same.

Napht/Li	0.11	0.29	0.49	1.05	1.40	1.95	3.26
	270 ± 90 nm (33%)	200 ± 70 nm (35%)	270 ± 70 nm (27%)	340 ± 110 nm (33%)	600 ± 200 nm (39%)	510 ± 150 nm (31%)	480 ± 160 nm (35%)
0.11		**1.00**	0.00	0.00	0.00	0.00	0.00
0.29	**1.00**		0.00	**0.00**	0.00	0.00	1.00
0.49	0.00	0.00		0.00	1.00	0.00	0.00
1.05	0.00	**0.00**	0.00		0.00	0.00	0.00
1.40	0.00	0.00	1.00	0.00		**0.01**	0.00
1.95	0.00	0.00	0.00	0.00	**0.01**		**0.00**
3.26	0.00	1.00	0.00	0.00	0.00	**0.00**	

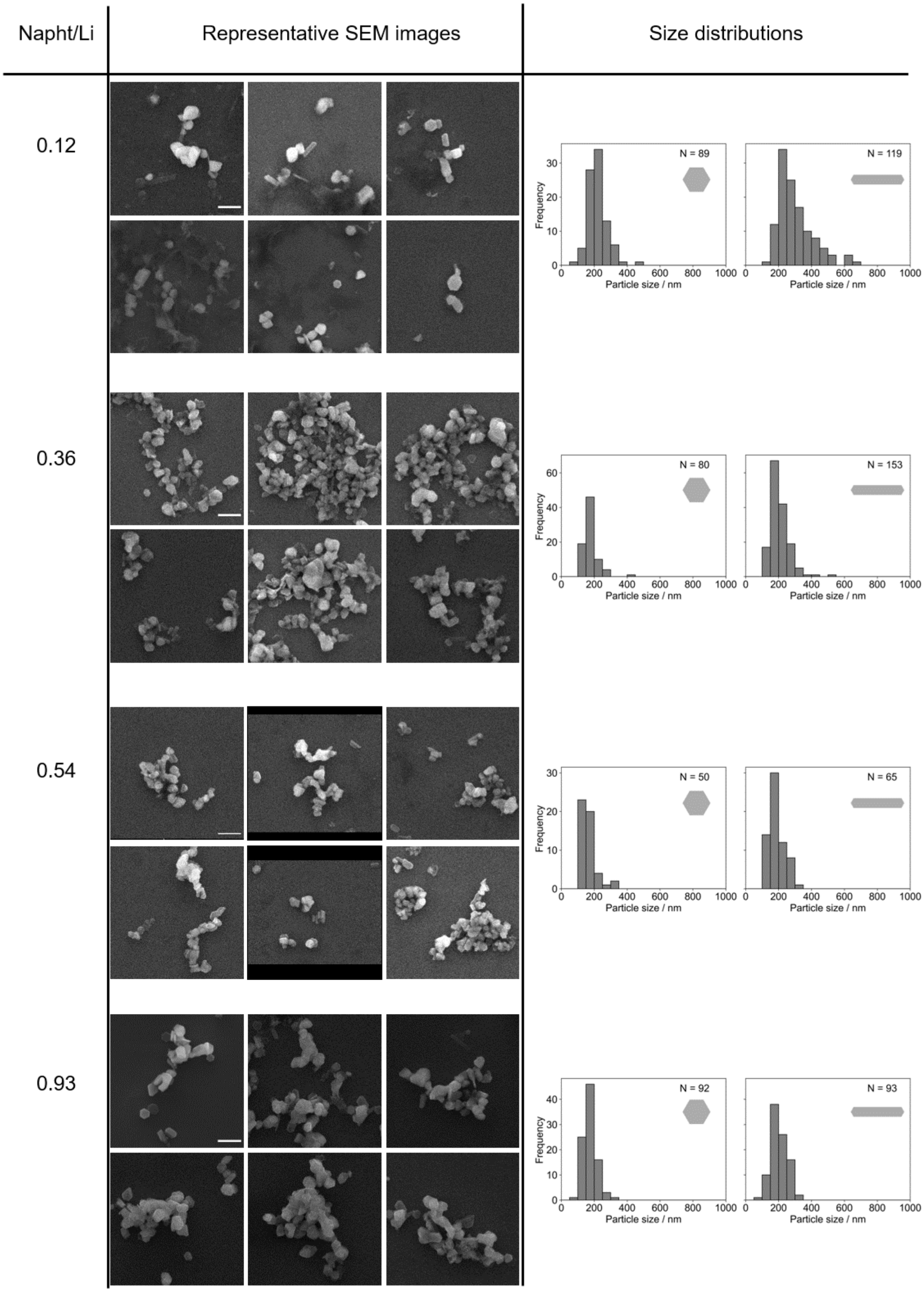

Figure S38. Representative SEM and size distributions of reactions with varying amounts of naphthalene at [$MgBu_2$] = 0.035 M (synthesis using 0.007 g Li, 0.44 mL $MgBu_2$ in 12.5 mL THF). Scale bars, 500 nm.

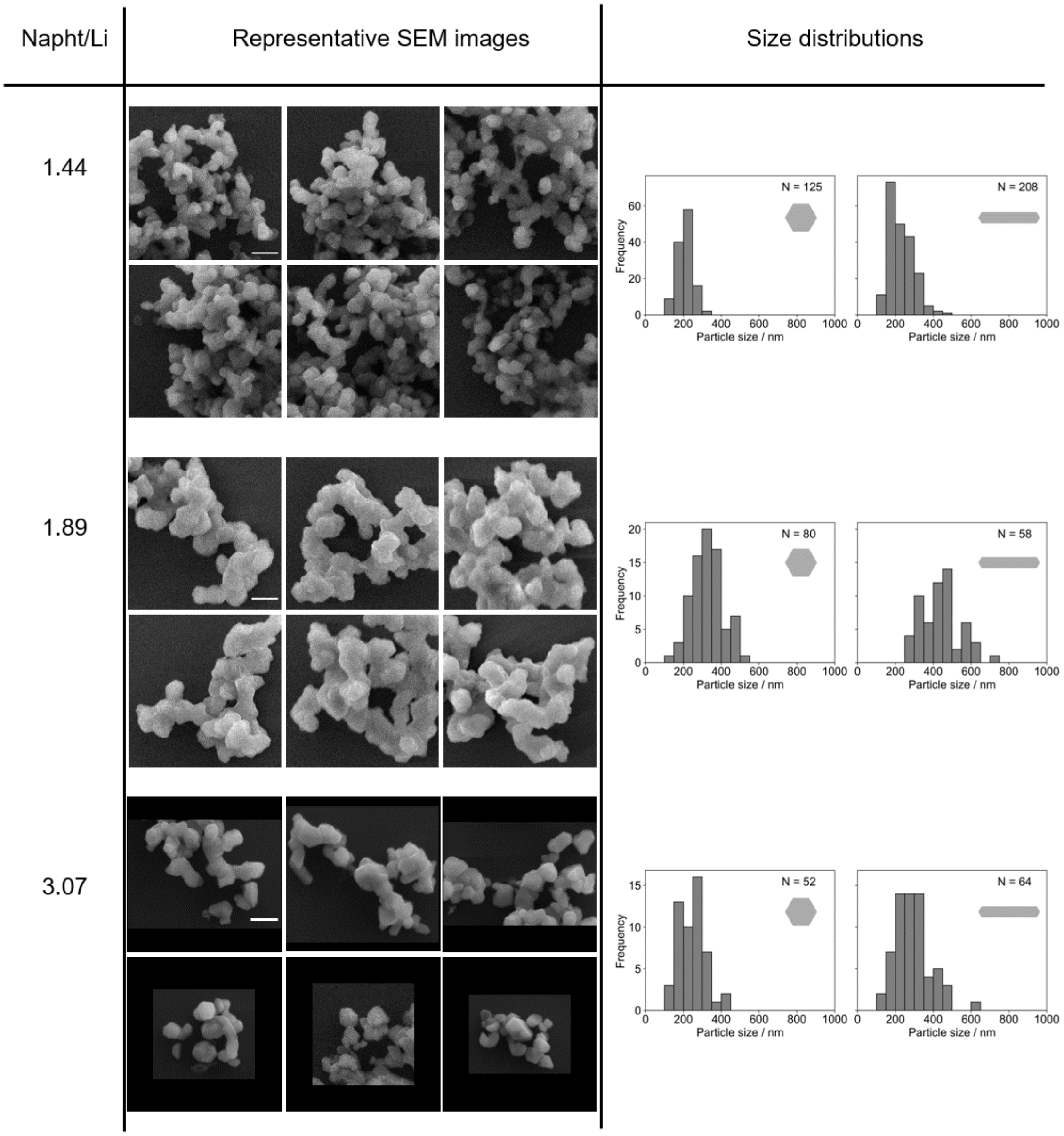

Figure S39. Representative SEM and size distributions of reactions with varying amounts of naphthalene at $[MgBu_2]$ = 0.035 M (synthesis using 0.007 g Li, 0.44 mL $MgBu_2$ in 12.5 mL THF). Scale bars, 500 nm.

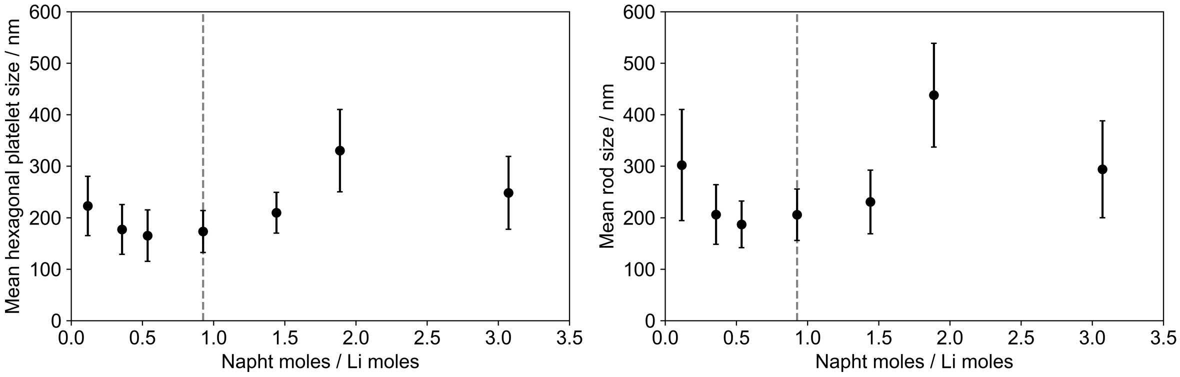

Figure S40. Comparison of hexagonal platelet and rod-shaped NP sizes for the reactions with varying amounts of naphthalene at $[MgBu_2]$ = 0.035 M as in Figures S38 and S39. The dotted line marks the standard ratio of naphthalene to Li.

Table S34. Hexagonal platelet sizes (2nd row) for reactions with varying amounts of naphthalene at $[MgBu_2]$ = 0.035 M (Figures S38–S40) and p-values from Games-Howell pairwise testing that sizes of hexagonal platelets are the same for each pair of reactions. Values are in bold where the two mean sizes are statistically the same.

Napht/Li	0.12	0.36	0.54	0.93	1.49	1.89	2.88
	220 ± 60 nm (26%)	180 ± 50 nm (27%)	170 ± 50 nm (30%)	170 ± 40 nm (23%)	210 ± 40 nm (19%)	330 ± 80 nm (24%)	250 ± 70 nm (28%)
0.12		0.00	0.00	0.00	**0.51**	0.00	0.31
0.36	0.00		**0.31**	0.00	**0.83**	0.00	0.00
0.54	0.00	**0.31**		0.00	0.00	0.00	0.00
0.93	0.00	0.00	0.00		0.00	0.00	0.00
1.49	**0.51**	**0.83**	0.00	0.00		0.00	0.01
1.89	0.00	0.00	0.00	0.00	0.00		**0.00**
2.88	**0.31**	0.00	0.00	0.00	0.01	0.00	

Table S35. Rod-shaped NP sizes (2nd row) for reactions with varying amounts of naphthalene at $[MgBu_2]$ = 0.035 M (Figures S38–S40) and p-values from Games-Howell pairwise testing that sizes of rod-shaped NPs are the same for each pair of reactions. Values are in bold where the two mean sizes are statistically the same.

Napht/Li	0.12	0.36	0.54	0.93	1.49	1.89	2.88
	300 ± 110 nm (36%)	210 ± 60 nm (28%)	190 ± 50 nm (24%)	210 ± 50 nm (24%)	230 ± 60 nm (27%)	440 ± 100 nm (23%)	300 ± 90 nm (32%)
0.12		0.00	0.00	0.00	0.00	0.00	1.00
0.36	0.00		**1.00**	0.00	**0.13**	0.00	0.00
0.54	0.00	**1.00**		0.00	0.00	0.00	0.00
0.93	0.00	0.00	0.00		0.00	0.00	0.00
1.49	0.00	**0.13**	0.00	0.00		0.00	0.00
1.89	0.00	0.00	0.00	0.00	0.00		0.00
2.88	**1.00**	0.00	0.00	0.00	0.00	0.00	

Solvent

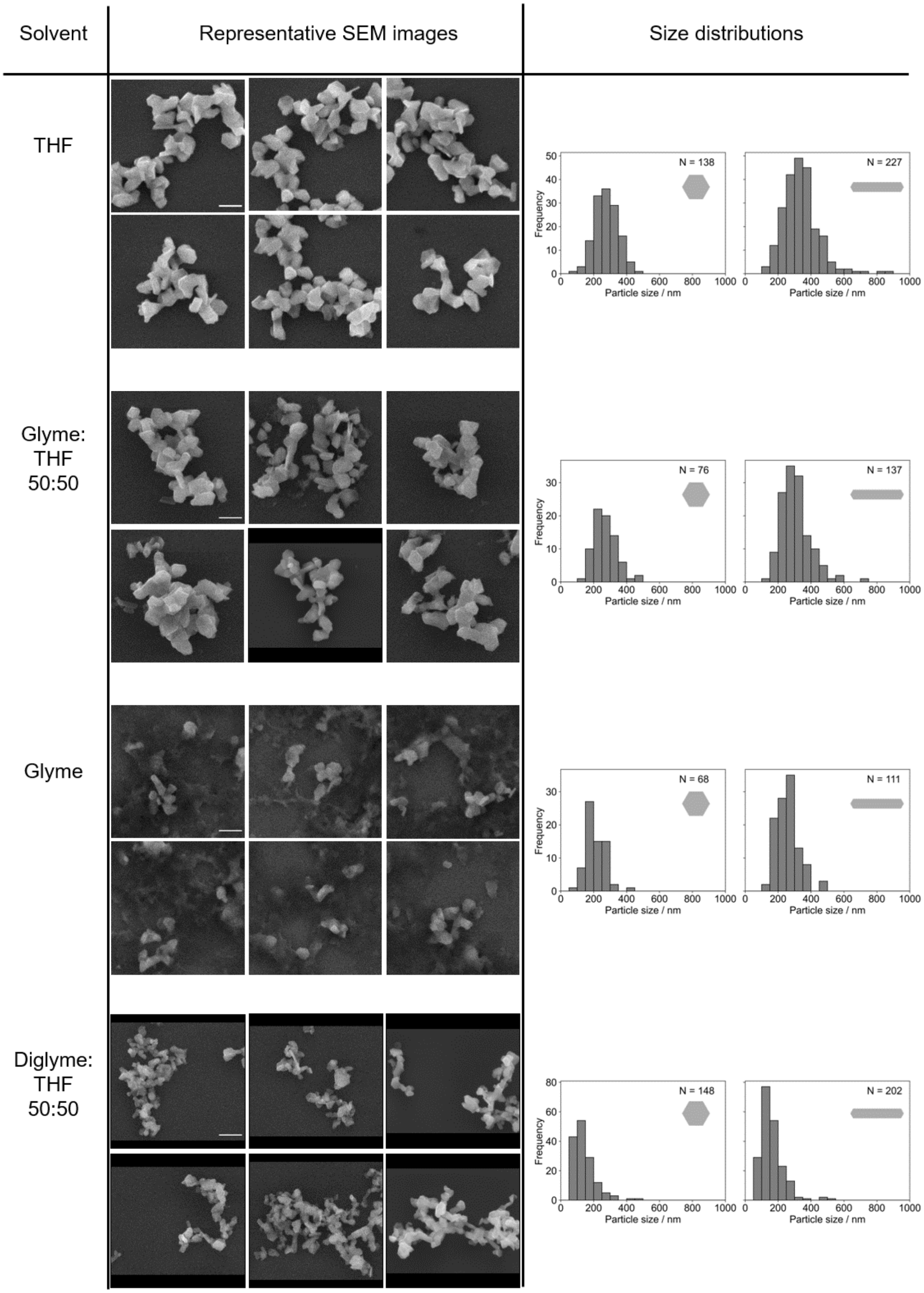

Figure S41. Representative SEM and size distributions of reactions with different solvents or solvent mixtures (50:50). Scale bars, 500 nm.

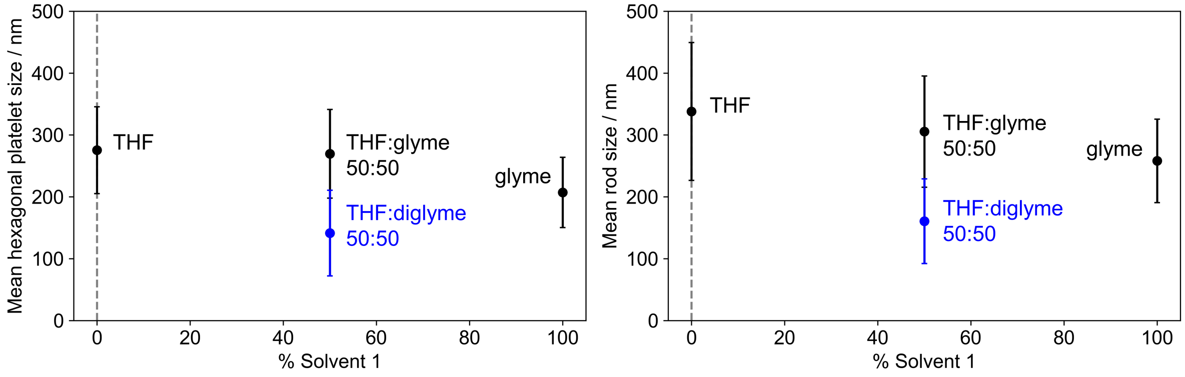

Figure S42. Comparison of hexagonal platelet and rod-shaped NP sizes for the reactions with different solvents as in Figure S41. In black, solvent 1 is glyme; in blue, solvent 1 is diglyme. Solvent 2 is THF. The dotted line marks the standard reaction (in THF).

Table S36. Hexagonal platelet sizes (2nd row) for reactions with different solvents or solvent mixtures (Figures S41, S42) and p-values from Games-Howell pairwise testing that sizes of hexagonal platelets are the same for each pair of reactions. Values are in bold where the two mean sizes are statistically the same.

	THF	Glyme:THF 50:50	Glyme	Diglyme:THF 50:50
	280 ± 70 nm (25%)	270 ± 70 nm (27%)	210 ± 60 nm (27%)	140 ± 70 nm (49%)
THF		**0.94**	0.00	0.00
Glyme:THF 50:50	**0.94**		0.00	0.00
Glyme	0.00	0.00		0.00
Diglyme:THF 50:50	0.00	0.00	0.00	

Table S37. Rod-shaped NP sizes (2nd row) for reactions with different solvents or solvent mixtures (Figures S41, S42) and p-values from Games-Howell pairwise testing that sizes of rod-shaped NPs are the same for each pair of reactions. No two mean sizes were statistically the same.

	THF	Glyme:THF 50:50	Glyme	Diglyme:THF 50:50
	340 ± 110 nm (33%)	310 ± 90 nm (29%)	260 ± 70 nm (26%)	160 ± 70 nm (43%)
THF		0.01	0.00	0.00
Glyme:THF 50:50	0.01		0.00	0.00
Glyme	0.00	0.00		0.00
Diglyme:THF 50:50	0.00	0.00	0.00	

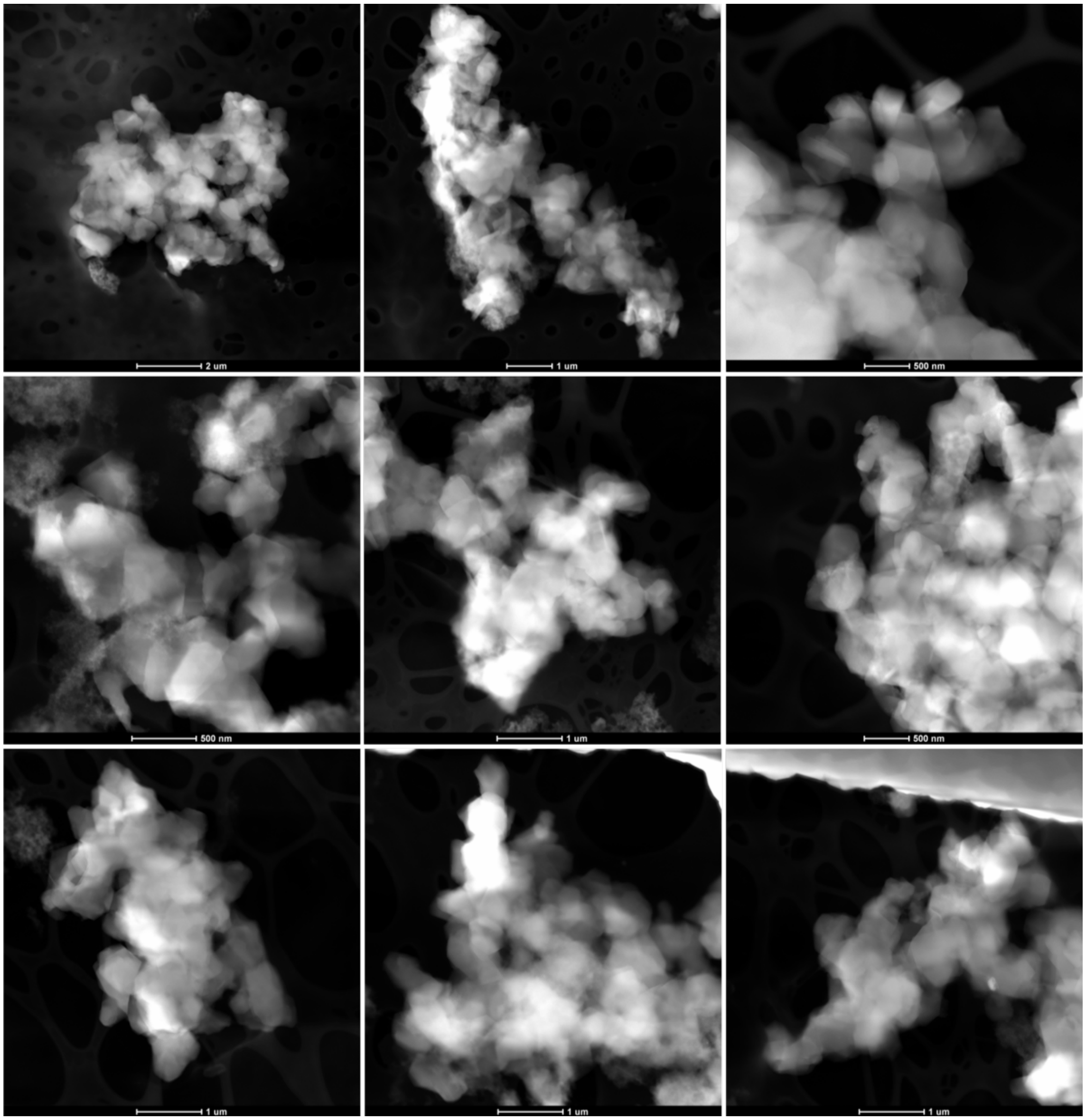

Figure S43. Representative HAADF-STEM images of aggregates from a reaction with 2.3:2.3:1 Li:naphthalene:$MgBu_2$, [$MgBu_2$] = 0.14 M and a reaction volume of 12.5 mL in a 25 mL flask at room temperature. The solid bar above the last two micrographs is the Cu grid bar from the support grid.

References

(1) Draine, B. T.; Flatau, P. J. Discrete-Dipole Approximation for Scattering Calculations. *J. Opt. Soc. Am. A* **1994**, *11*, 1491.

(2) Flatau, P. J.; Draine, B. T. Fast near Field Calculations in the Discrete Dipole Approximation for Regular Rectilinear Grids. *Opt. Express* **2012**, *20*, 1247.

(3) Boukouvala, C.; Ringe, E. Wulff-Based Approach to Modeling the Plasmonic Response of Single Crystal, Twinned, and Core-Shell Nanoparticles. *J. Phys. Chem. C* **2019**, *123*, 25501–25508.

(4) Palik, E. D. Handbook of Optical Constants of Solids III; Academic Press: New York, 1998.

(5) Lide, D. R. CRC Handbook of Chemistry and Physics, 80th ed.; CRC Press: New York, 2000.

(6) Peña, F. de la; Prestat, E.; Fauske, V. T.; Burdet, P.; Furnival, T.; Jokubauskas, P.; Nord, M.; Ostasevicius, T.; Lähnemann, J.; MacArthur, K. E.; et al. Hyperspy/Hyperspy: Release v1.6.2. **2021**.
